# Supplementary material for: Poly(ADP-Ribose) Links the DNA Damage Response and Biomineralization
Source: Cell Rep. 2019 Jun 11;27(11):3124–3138.e13. doi: 10.1016/j.celrep.2019.05.038 (PMC6581741; doi:10.1016/j.celrep.2019.05.038)
Supplement: Document S1. Figures S1–S7 and Tables S1 and S2 [file mmc1.pdf]

Cell Reports, Volume *27*

Supplemental Information

Poly(ADP-Ribose) Links the DNA Damage Response and Biomineralization

Karin H. Müller, Robert Hayward, Rakesh Rajan, Meredith Whitehead, Andrew M. Cobb, Sadia Ahmad, Mengxi Sun, Ieva Goldberga, Rui Li, Uliana Bashtanova, Anna M. Puszkarska, David G. Reid, Roger A. Brooks, Jeremy N. Skepper, Jayanta Bordoloi, Wing Ying Chow, Hartmut Oschkinat, Alex Groombridge, Oren A. Scherman, James A. Harrison, Anja Verhulst, Patrick C. D'Haese, Ellen Neven, Lisa-Maria Needham, Steven F. Lee, Catherine M. Shanahan, and Melinda J. Duer

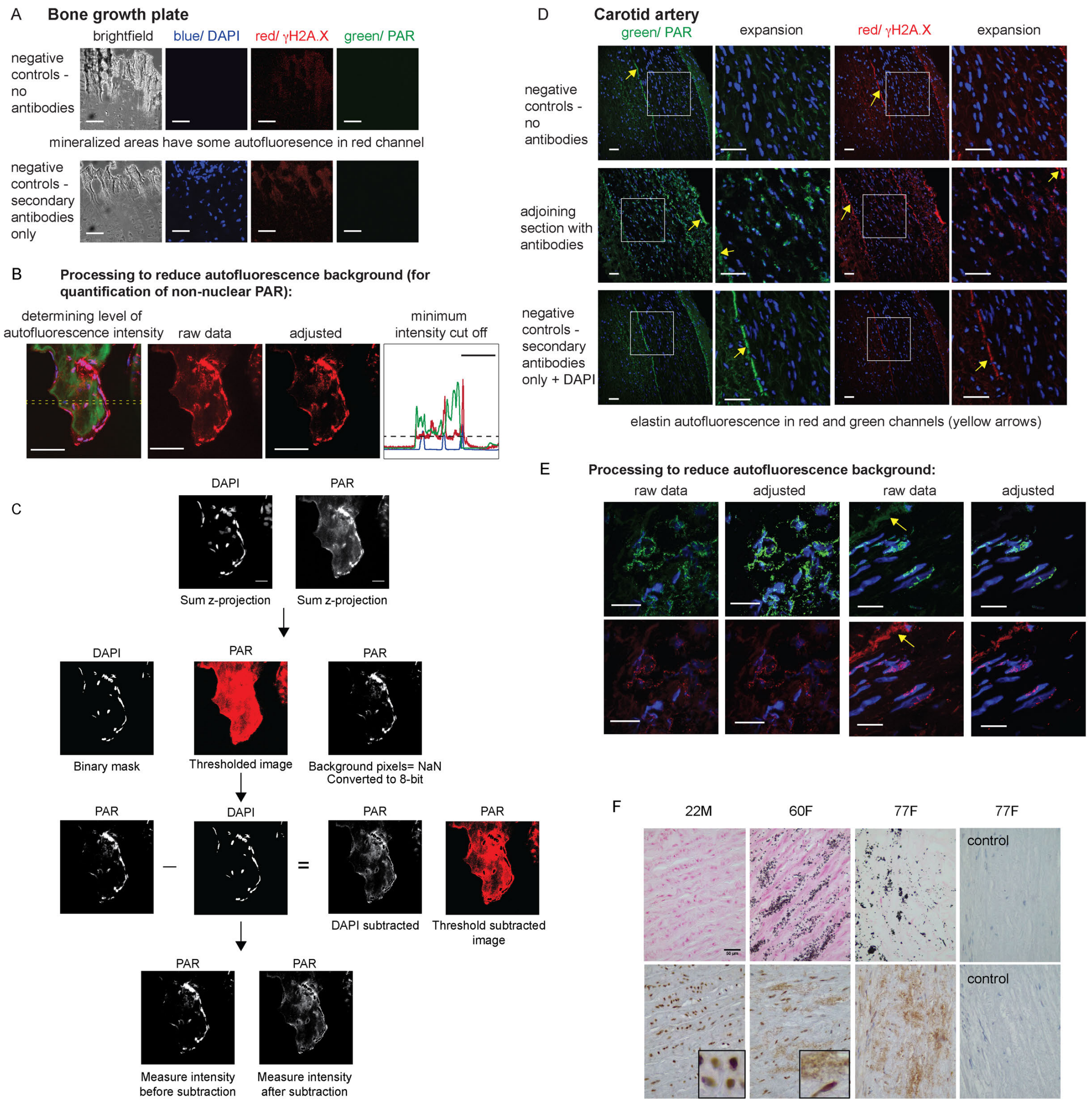

Figure S1: Controls and image processing for Figure 1

(A) Negative controls for confocal imaging of bone growth plate in Fig 1.

(B) Image processing for bone growth plate images in Fig 1A, B.

(C) Details of image processing and quantification method for determining fraction of extranuclear PAR from confocal images of mineralized and non-mineralized areas of bone growth plate for results shown in Fig 1A.

(D) Negative controls for carotid artery confocal imaging in Fig 1 (maximum intensity projections of z-stacks). Yellow arrows indicate autofluorescence from elastin.

(E) Image processing for vessel images in Fig 1C. Images in (A) – (E) are maximum intensity projections of z-stacks; scale bars, 50 μm.

(F) Examples of the immunohistochemistry images used to quantify ratio of extranuclear/ nuclear PAR and extent of vessel calcification in Fig 1D.

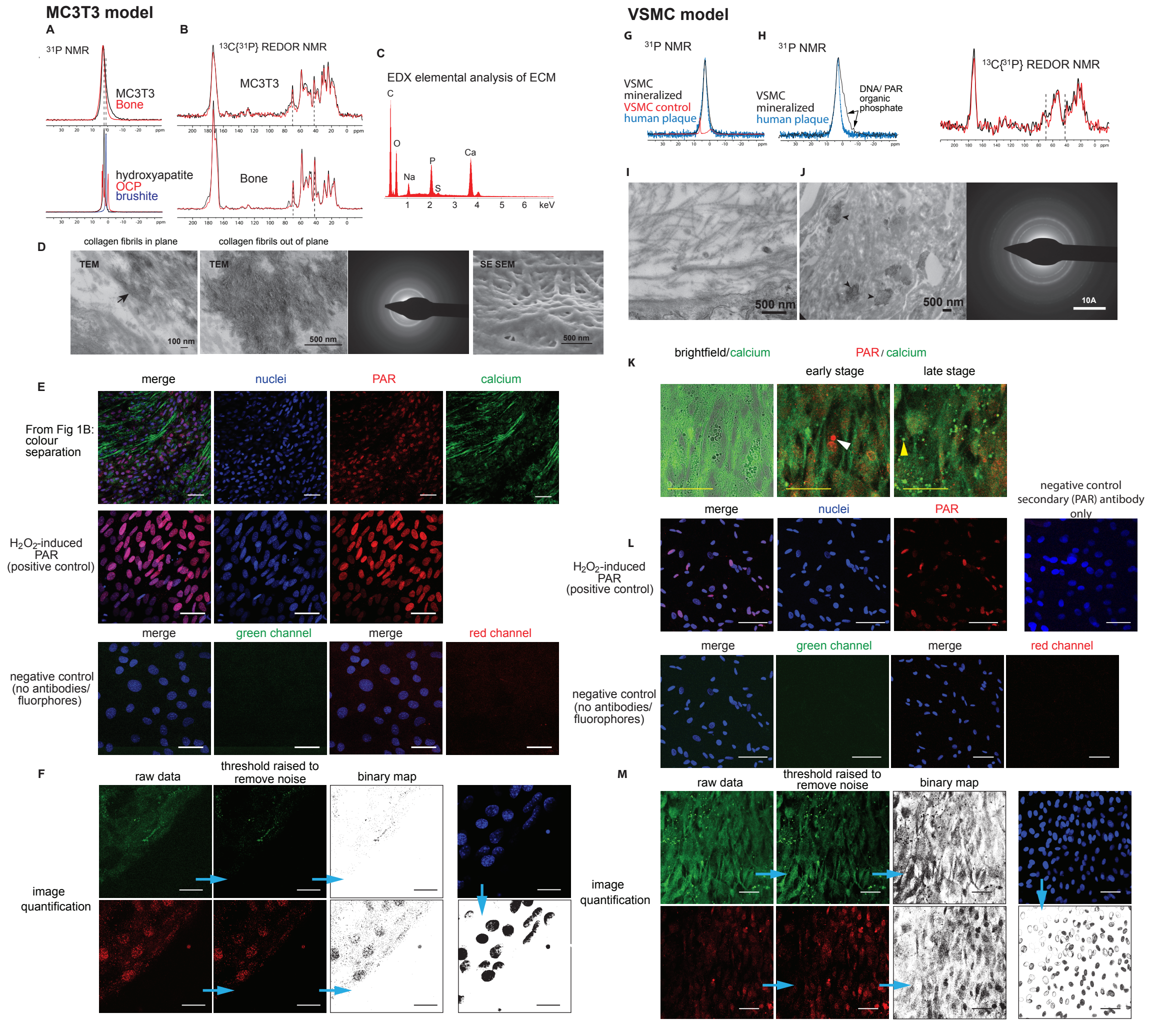

Figure S2: Supporting data for MC3T3 and b/h VSMC in vitro models of extracellular calcification used in Figure 2.

MC3T3 model

(A) ^{31}P direct polarization, magic-angle spinning (DP-MAS) NMR spectra of MC3T3 mineralized matrix compared with native mouse bone and pure calcium phosphate phases, showing that the mineral in the MC3T3 model is bone-like and apatitic in form.

(B) ^{13}C{^{31}P}Rotational Echo Double Resonance (REDOR) NMR spectra of MC3T3 mineralized matrix and native mouse bone. Black – reference spectrum, red = REDOR-dephased spectrum. Signals from collagen triple helix hydroxyproline (Cγ, 70 ppm) and glycine (Cα, 42.5 ppm) and indicated with dotted lines. Decreased intensity in the REDOR (red) spectrum compared to reference (black) are from ^{13}C sites within 1 nm of ^{31}P, i.e. close to phosphatic mineral. Similarities in dephasing pattern between MC3T3 and bone REDOR spectra indicate similarity in mineral-organic matrix interactions.

(C) EDX element composition analysis of MC3T3 mineralised matrix from TEM in (D).

(D) Electron microscopy assessment of mineral morphology and spatial distribution in MC3T3 mineralized matrix, showing mineral aligning with collagen fibrils (fibrils in plane) and curving around fibrils (fibrils out of plane) similarly to native bone (*47*).

(E) Controls for confocal imaging in Fig 2B – E. Scale bars, 50 μm.

(F) Details of image processing for quantification of fraction of extracellular PAR. Scale bar, 20 μm.

VSMC models.

(G), (H) ^{31}P direct polarization, magic-angle spinning (DP-MAS) NMR spectra of (G) bVSMC and (H) hVSMC mineralized matrix, compared with calcified human carotid calcification. (H) also shows a REDOR $^{13}C\{^{31}P\}$ NMR spectrum for hVSMC calcified matrix. Signals from collagen hydroxyproline and glycine (dotted lines, see (B) for details and comparison) are typically weak in these samples.

(I), **(J)** TEM images of bVSMC extracellular matrix under (I) non-calcifying and (J) calcifying conditions. Mineralized matrix shows patches of mineral (arrowheads) a few microns in dimensions. SAED of these patches (right) indicates crystalline material with multiple crystallite orientations.

(K) Expansions of areas in the confocal images in Fig 2G, H, showing PAR containing vesicles (red/ orange, white arrowhead) and mineralized patches (green, yellow arrowhead) typically a few microns in diameter. Controls for confocal imaging in Fig 2G – J.

(M) Details of image processing for quantification of fraction of extracellular PAR.

Scale bars, 50 μm for (K) – (M).

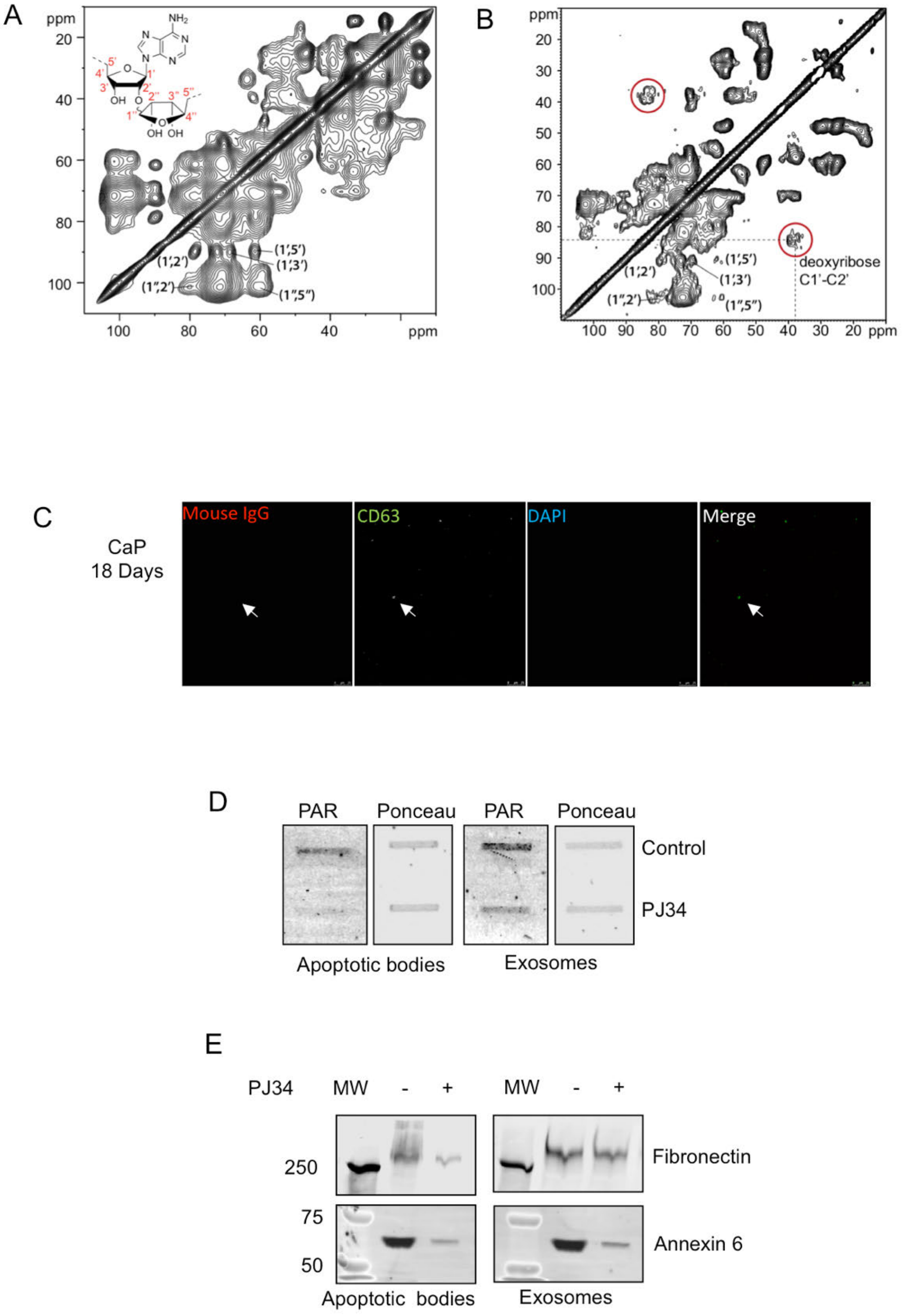

Figure S3: Supplemental data for Figure 3

(A-B) 2D ^{13}C-^{13}C proton-driven spin-diffusion correlation NMR spectra of in vitro ECM from cell cultures supplemented with U-^{13}C-glucose for **(A)** bVSMC and **(B)** foetal sheep osteoblasts. The spectra show signals only from parts of molecules that have been ^{13}C-labelled from the U-^{13}C-glucose added to the cell culture medium during growth of the ECM. Ribose rings and collagen native glycosylation are the main components labelled in these spectra. The signal frequencies (denoted as chemical shifts) depend on the molecule the ribose ring is in. Thus, signals from ribose rings in PAR or in DNA appear at distinctive and different frequencies. The signal frequencies for PAR and DNA are established from NMR spectra of the pure materials (Chow *et al.*, 2014).
(C) Absence of DAPI staining confirming complete cell lysis and removal of nuclear material. IgG antibody controls for PAR are negative.
(D) Protein slot blot analysis of PAR content in VSMC-derived EV populations isolated by differential ultracentrifugation (n = 3). Note inhibition with PJ34.
(E) Boronate bead enrichment for PAR and western blot of EVs shows decreased binding of fibronectin and annexin 6 to the beads after PJ34 treatment (n = 2).

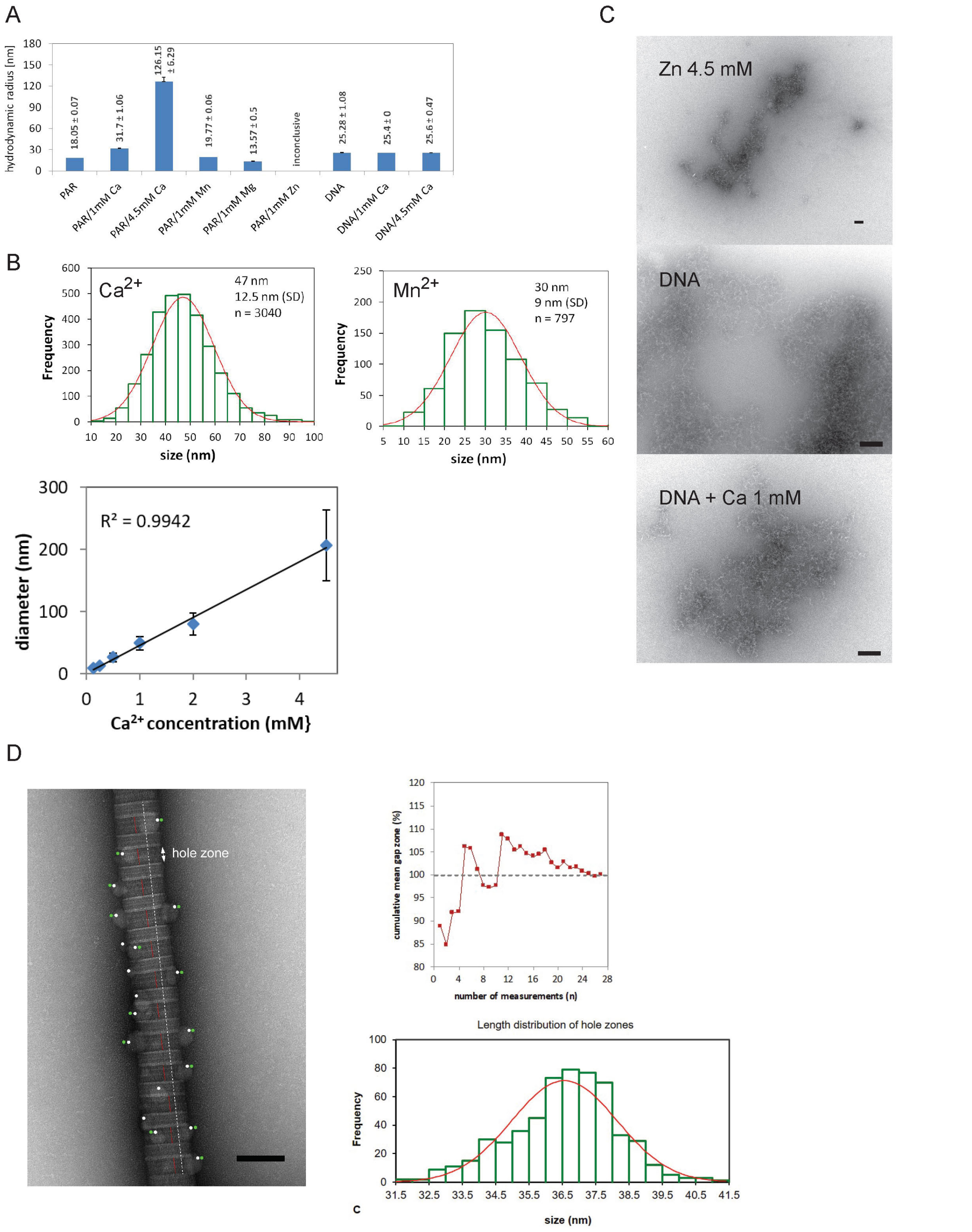

Figure S4: Data analysis and additional data/ controls for Figure 4.

(A) Hydrodynamic radii of PAR in the absence and presence of various divalent cations as measured by DLS; DNA was used as a control. Values given are the mean ± SD.

(B) Graphs showing the size-frequency distributions of PAR spheres after addition of 1 mM calcium and 1 mM manganese, respectively. The graph below indicates a linear trend in the tested concentration range with a highly significant R^2.

(C) TEM images of PAR + 4.5 mM Zn^{2+} (showing absence of spheres), and as controls, DNA alone and DNA + 1 mM Ca^{2+}.

(D) Details of how PAR-Ca sphere localization on hole/ overlap zones of collagen fibrils was determined. Left: Example TEM image: all PAR-Ca spheres bound to the collagen fibril were labeled with a white dot (irrespective of their location); the PAR-Ca spheres bound to the fibril hole zones were labeled with a green dot. The length of the fibril was measured along a certain number of D-periods (white line), as well as the length of the individual hole zones (red lines). Graph (right, top) plotting the cumulative mean gap zone in % with increasing number of measurements (n = 27 images). After assessing about 20 images, the cumulative mean gap zone stabilizes towards 100 %, indicating that a statistically sufficient number of measurements have been performed. Right, bottom: Length-frequency distribution of the measured hole zone lengths (mean ± SD). Scale bars 100 nm for (B) – (D).

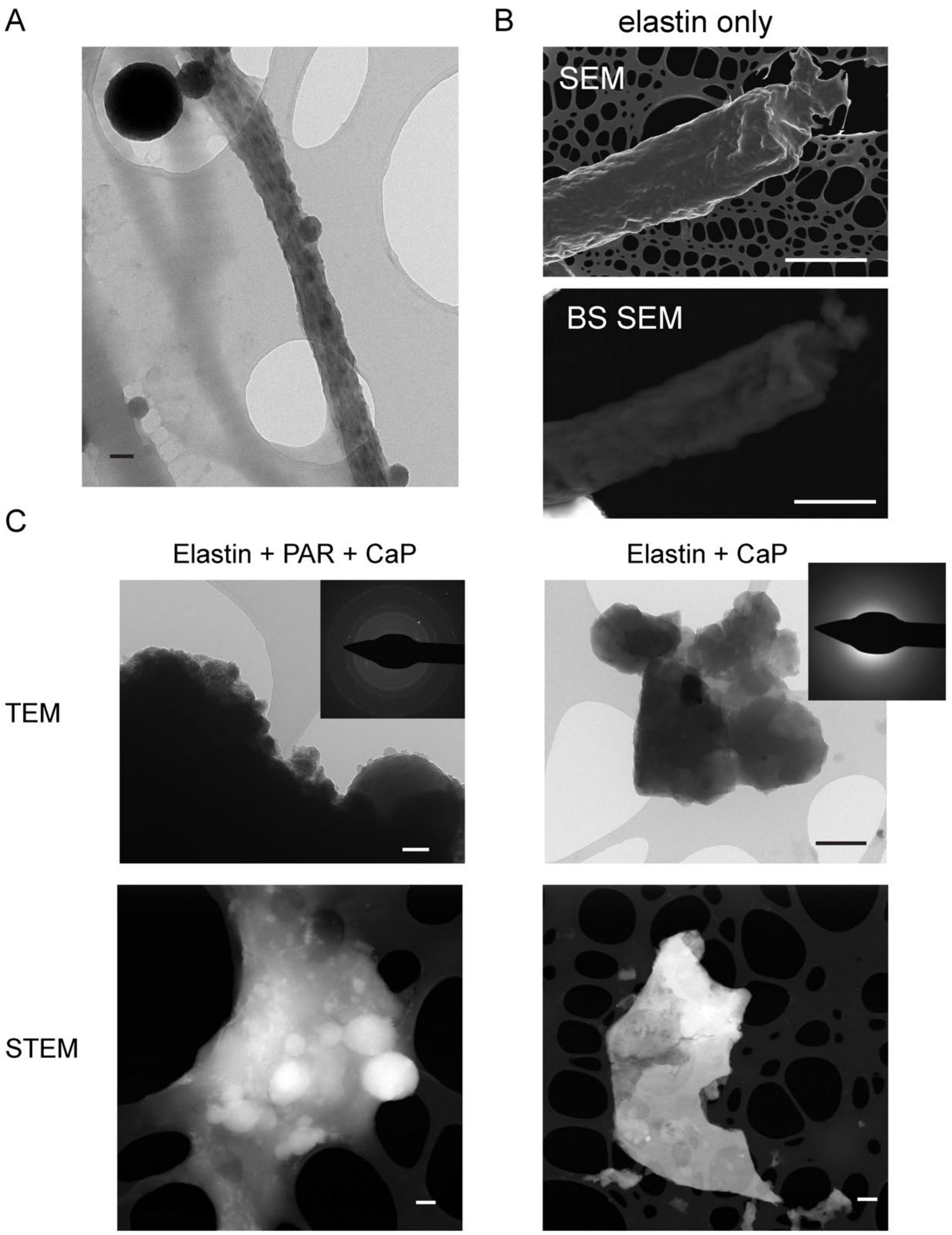

Figure S5: Additional data and controls for Figure 5

(A) TEM image of collagen fibril after 10 days of incubation with PAR and 4.5 mM Ca^{2+} ($CaCl_2$), 2.1 mM PO_4^{3-} (K_2HPO_4) TRIS buffered solution showing mineral formation on the fibril and spheres binding/ adjacent to the calcified fibril. Scale bar, 100 nm.

(B) SEM images of elastin only as controls for the SEM image in Fig 6C. Scale bars, 5 μm.

(C) Additional TEM and STEM images for elastin + PAR (left, scale bars 100 nm) and elastin alone (right, scale bars, 500 nm) after incubation with 4.5 mM Ca^{2+} ($CaCl_2$), 2.1 mM PO_4^{3-} (K_2HPO_4) TRIS buffered solution.

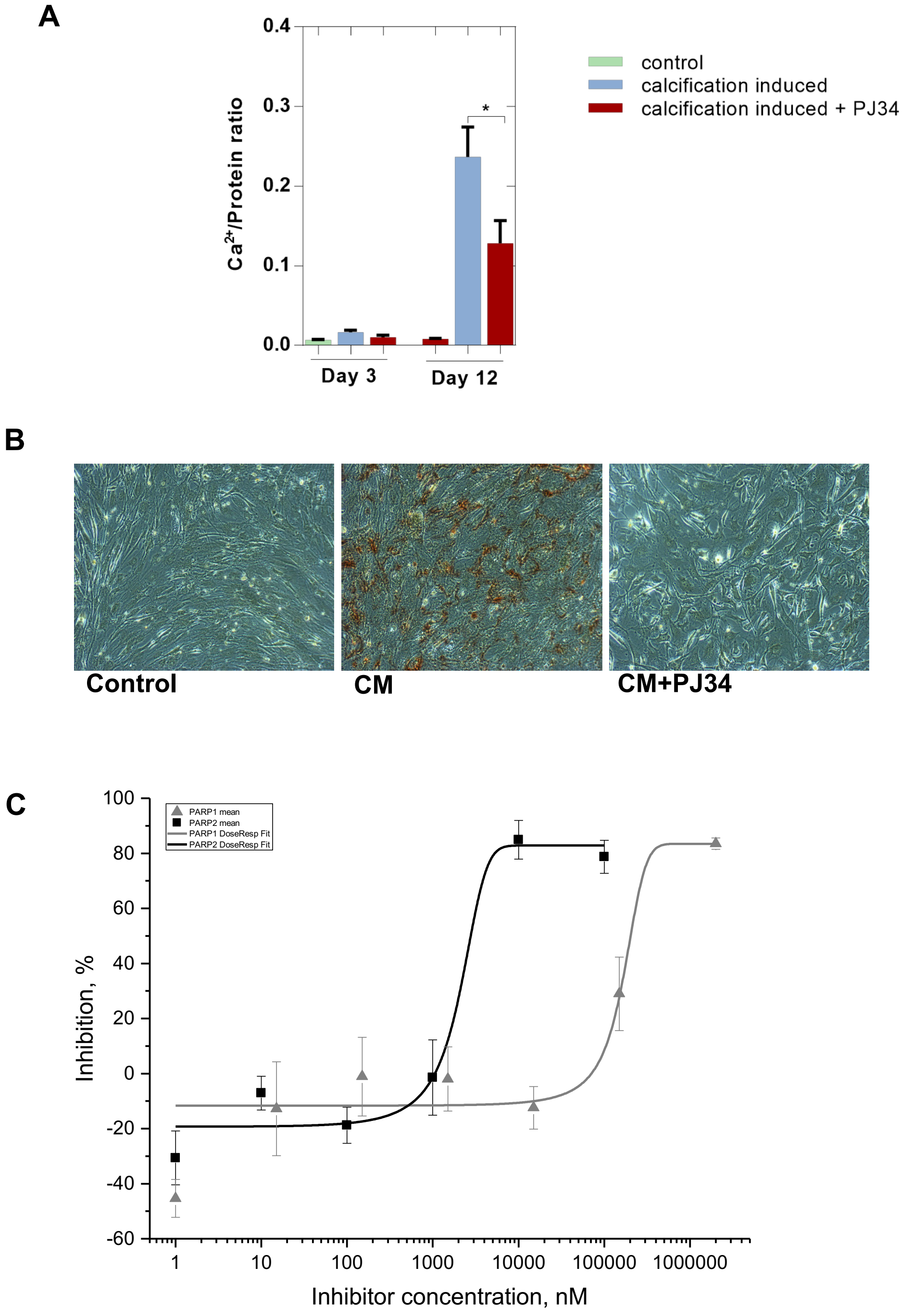
A
Ca2+/Protein ratio
0.4
0.3
0.2
0.1
0.0
*
Day 3
Day 12
control
calcification induced
calcification induced + PJ34
B
Control
CM
CM+PJ34
C
PARP1 mean
PARP2 mean
PARP1 DoseResp Fit
PARP2 DoseResp Fit
Inhibition, %
100
80
60
40
20
0
-20
-40
-60
1
10
100
1000
10000
100000
1000000
Inhibitor concentration, nM

Figure S6: The effect of PARP inhibitor PJ34 in the bovine VSMC (bVSMC) model as additional data for Figure 6 and the inhibitor effect of minocycline on PARP1/2 activity to support the data in Figures 6F – H.

(A) The PARP inhibitor PJ-34 (10 mM) inhibited calcification in bVSMCs induced to mineralize by treatment with 10 mM ß-glycerophosphate (ßGP) + 0.1 mM ascorbic acid 2-phosphate + 10 nM dexamethasone (CM) (n = 3).

(B) Mineralization in the bVSMC cultures was visualized with Alizarin Red S staining

(C) Dose-response curve for minocycline against human PARP1 and 2. Minocycline is a selective inhibitor of PARP2. In *in vitro* PARP activity assay minocycline inhibits PARP2 and PARP1 activity with an IC50 of 2.8 μM and 204.5 μM correspondingly.

A

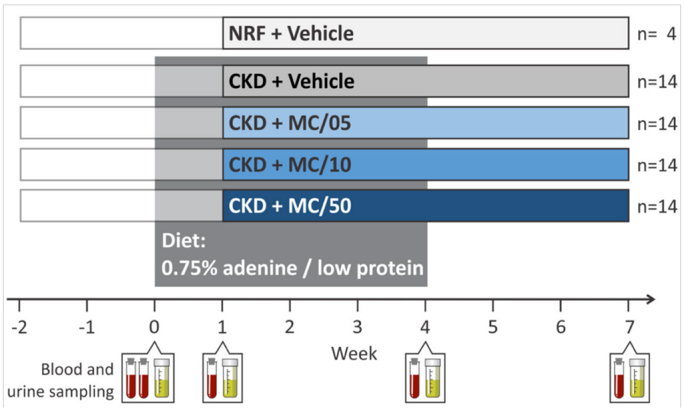

B

Biochemical Parameters		NRF + vehicle	CKD + vehicle	CKD + MC/50
**Creatinine Clearance** ml/min/100g body weight	**Baseline**	0.425 ± 0.054	0.523 ± 0.020	0.545 ± 0.021
	**Week 1**	0.301 ± 0.020	0.189 ± 0.017[a]	0.199 ± 0.019[a]
	**Week 4**	0.330 ± 0.005	0.039 ± 0.003[a]	0.034 ± 0.003[a]
	**Week 7**	0.330 ± 0.016	0.083 ± 0.008[a]	0.056 ± 0.005[a,b]
**Serum Phosphorus** mg/dl	**Baseline**	9.23 ± 0.80	9.83 ± 0.44	11.49 ± 0.29[a]
	**Week 1**	8.27 ± 0.73	10.07 ± 0.50	9.60 ± 0.84
	**Week 4**	10.61 ± 1.36	25.08 ± 0.86[a]	25.18 ± 0.91[a]
	**Week 7**	7.32 ± 0.64	11.05 ± 0.51[a]	12.35 ± 0.58[a]
**Serum Calcium** mg/dl	**Baseline**	11.61 ± 0.42	11.02 ± 0.22	9.07 ± 0.17[a,b]
	**Week 1**	8.46 ± 0.19	7.77 ± 0.11[a]	6.94 ± 0.07[a,b]
	**Week 4**	8.30 ± 0.17	6.82 ± 0.19[a]	6.28 ± 0.18[a]
	**Week 7**	7.02 ± 0.16	5.84 ± 0.35	4.84 ± 0.33[a]

Renal function and mineral metabolism. Creatinine clearance, serum phosphorus and calcium values (mean ± SEM) throughout the study course. [a]p<0.05 versus NRF + vehicle group. [b]p<0.05 versus CKD + vehicle. NRF: normal renal function. CKD: chronic kidney disease. MC/50: daily treatment with 50 mg/kg minocycline.

C

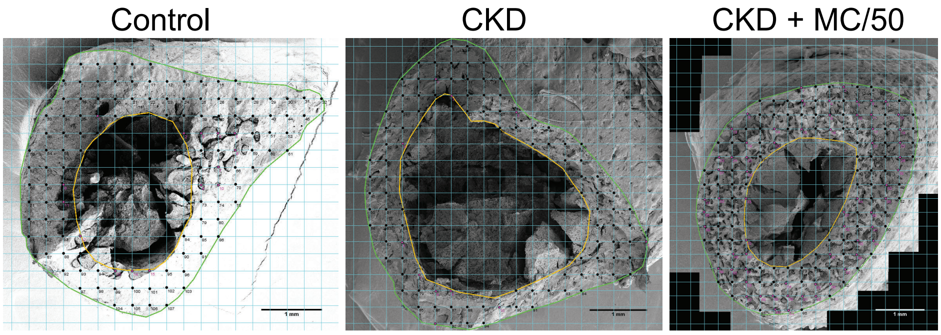

Figure S7: Further information on the CKD in vivo model used in Figure 7.

(A) Cartoon showing the study protocol for the rat CKD model.

(B) Table showing the biochemical parameters for renal function and mineral metabolism in the rat CKD model during the 6 week induction period.

(C) Details of the grids used to estimate the degree of calcification in each bone sample. Scale bars, 500 μm.

Table S1: Details of the blood vessel samples used in extranuclear PAR and calcification content analysis in Figure 1.

n	Age/Sex/Vessel	Calcification	PAR Localization		Ratio
		(%)	Nuclear	Cytoplasmic/Extracellular	ExtraC/Nuclear
1	16F,26A	0.2	100	0	0.0
2	18M,9A	0	100	0	0.0
3	22M,2A	0	100	0	0.0
4	52M,3A	0.4	75	25	0.3
5	53F,4A	1.8	75	25	0.3
6	53F,8A	0.8	100	0	0.0
7	55F,10A	1.1	75	25	0.3
8	59M,25C	3.3	75	25	0.3
9	60F,11A	10.1	25	75	3.0
10	63F,3A	2	75	25	0.3
11	64F,10A	3	70	30	0.4
12	65M,22C	2.4	50	50	1.0
13	66F,26C	0	75	5	0.1
14	66M,17A	12.1	25	75	3.0
15	67M,7A	5.6	75	25	0.3
16	71F,2A	8.4	50	50	1.0
17	71M,12C	8.9	25	75	3.0
18	72M,8C	4.6	75	25	0.3
19	74F,17C	9.6	50	50	1.0
20	74F,62C	3	25	75	3.0
21	75M,20C	3.1	75	25	0.3
22	77F,53C	9.2	25	75	3.0
23	78M,19C	8	25	75	3.0

Table S2: Human PARP1 and 2 enzyme inhibition, IC50 values determined for inhibitors used in Fig 6.

Inhibitor	IC50	
	PARP1	PARP2
PJ-34	86.5 nM (R^2 = 0.94)	282 nM (R^2 = 0.99)
Niraparib	205.3 nM (R^2 = 0.99)	>100 µM
Olaparib	9.5 nM (R^2 = 0.97)	>100 µM
Rucaparib	8.8 nM (R^2 = 0.99)	>10 µM
Veliparib	9.3 nM (R^2 = 1)	11.1 µM (R^2 = 0.84)
Minocycline	204.5 µM (R^2 = 0.69)	2.8 µM (R^2 = 0.82)
