# Supplementary material for: Poly(ADP-Ribose) Links the DNA Damage Response and Biomineralization
Source: Cell Rep. 2019 Jun 11;27(11):3124–3138.e13. doi: 10.1016/j.celrep.2019.05.038 (PMC6581741; doi:10.1016/j.celrep.2019.05.038)
Supplement: Document S2. Article plus Supplemental Information [file mmc2.pdf]

Cell Reports

Article

Poly(ADP-Ribose) Links the DNA Damage Response and Biomineralization

Graphical Abstract

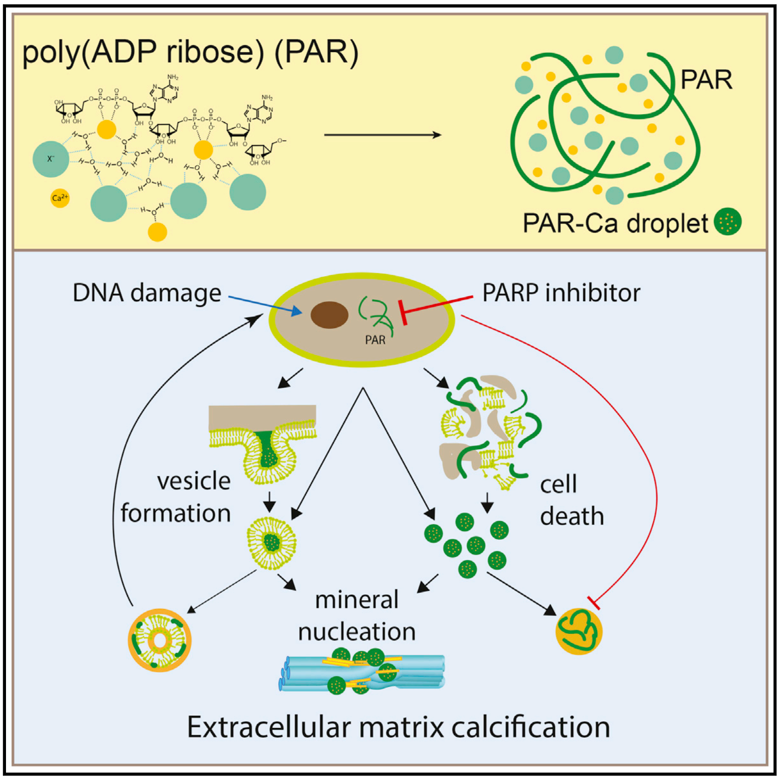

Highlights

- Poly(ADP-ribose) is found close to ECM calcification in developing bone and arteries
- Poly(ADP-ribose) is produced in response to oxidative stress and delivered to the ECM
- Poly(ADP-ribose) forms dense liquid droplets with calcium ions
- Inhibiting PARP enzyme activity blocks calcification *in vitro* and *in vivo*

Authors

Karin H. Müller, Robert Hayward, Rakesh Rajan, ..., Steven F. Lee, Catherine M. Shanahan, Melinda J. Duer

Correspondence

cathy.shanahan@kcl.ac.uk (C.M.S.), mjd13@cam.ac.uk (M.J.D.)

In Brief

Müller et al. investigate the physicochemical process of extracellular matrix calcification in both physiological (bone) and pathological (vascular calcification) contexts. They find that oxidative stress-induced poly(ADP-ribose) nucleates calcium phosphate mineral crystals on extracellular matrix substrates and that calcification is inhibited by poly(ADP-ribose) polymerase (PARP) enzyme inhibitors.

Müller et al., 2019, Cell Reports *27*, 3124–3138
June 11, 2019 © 2019 The Author(s).
https://doi.org/10.1016/j.celrep.2019.05.038

CellPress

OPEN ACCESS
CellPress

Cell Reports
Article

Poly(ADP-Ribose) Links the DNA Damage Response and Biomineralization

Karin H. Müller,[1] Robert Hayward,[2] Rakesh Rajan,[1] Meredith Whitehead,[2] Andrew M. Cobb,[2] Sadia Ahmad,[2] Mengxi Sun,[2] Ieva Goldberga,[1] Rui Li,[1] Uliana Bashtanova,[1] Anna M. Puszkarska,[1] David G. Reid,[1] Roger A. Brooks,[3] Jeremy N. Skepper,[4] Jayanta Bordoloi,[2] Wing Ying Chow,[5] Hartmut Oschkinat,[5] Alex Groombridge,[6] Oren A. Scherman,[6] James A. Harrison,[7] Anja Verhulst,[8] Patrick C. D'Haese,[8] Ellen Neven,[8] Lisa-Maria Needham,[1] Steven F. Lee,[1] Catherine M. Shanahan,[2,*] and Melinda J. Duer[1,9,*]

[1]Department of Chemistry, University of Cambridge, Lensfield Road, Cambridge CB2 1EW, UK
[2]BHF Centre of Research Excellence, Cardiovascular Division, James Black Centre, King's College London, 125 Coldharbour Lane, London SE5 9NU, UK
[3]Division of Trauma and Orthopaedic Surgery, University of Cambridge, Box 180, Addenbrooke's Hospital, Hills Road, Cambridge CB2 2QQ, UK
[4]Cambridge Advanced Imaging Centre, Department of Physiology, Development and Neurobiology, Downing Site, Tennis Court Road, Cambridge CB2 3DY, UK
[5]Leibniz Forschungsinstitut für Molekulare Pharmakologie (FMP) im Forschungsverbund Berlin e.V., Campus Berlin-Buch, Robert-Roessle-Str 10, 13125 Berlin, Germany
[6]Melville Laboratory for Polymer Synthesis, Department of Chemistry, University of Cambridge, Lensfield Road, Cambridge CB2 1EW, UK
[7]Cycle Pharmaceuticals Ltd, Bailey Grundy Barrett Building, Little St. Mary's Lane, Cambridge CB2 1RR, UK
[8]Laboratory of Pathophysiology, Department of Biomedical Sciences, University of Antwerp, Universiteitsplein 1, 2610 Wilrijk, Belgium
[9]Lead Contact
*Correspondence: cathy.shanahan@kcl.ac.uk (C.M.S.), mjd13@cam.ac.uk (M.J.D.)
https://doi.org/10.1016/j.celrep.2019.05.038

SUMMARY

Biomineralization of the extracellular matrix is an essential, regulated process. Inappropriate mineralization of bone and the vasculature has devastating effects on patient health, yet an integrated understanding of the chemical and cell biological processes that lead to mineral nucleation remains elusive. Here, we report that biomineralization of bone and the vasculature is associated with extracellular poly(ADP-ribose) synthesized by poly(ADP-ribose) polymerases in response to oxidative and/or DNA damage. We use ultrastructural methods to show poly(ADP-ribose) can form both calcified spherical particles, reminiscent of those found in vascular calcification, and biomimetically calcified collagen fibrils similar to bone. Importantly, inhibition of poly(ADP-ribose) biosynthesis *in vitro* and *in vivo* inhibits biomineralization, suggesting a therapeutic route for the treatment of vascular calcifications. We conclude that poly(ADP-ribose) plays a central chemical role in both pathological and physiological extracellular matrix calcification.

INTRODUCTION

Biomineralization is the deposition of mineral particles within a proteinaceous organic matrix. In bone, this is an essential physiological process (Rey et al., 2009), but extensive pathological calcification of soft tissues, in particular the vasculature (Abedin et al., 2004; Johnson et al., 2006; Shao et al., 2006; Demer and Tintut, 2008; Kapustin and Shanahan, 2009), commonly occurs in association with disease. Determining how this complex chemical process is controlled is relevant to both bone development and the treatment of detrimental conditions such as "hardening of the arteries." Despite increased understanding of the cell biological processes involved in biomineralization, the chemical mechanism of mineral nucleation remains elusive. Any proposed mechanism must explain the onset of mineralization, the spatial distribution of the mineral, its particle morphology, and why calcium is the dominant metal ion in the mineral. In turn, these must be consistent with cellular biomolecular synthesis (Combes et al., 2016).

In both bone and soft tissues, collagen is the predominant extracellular matrix (ECM) substrate mineralized, and apatitic calcium phosphate, with various ionic substitutions (Combes et al., 2016; You et al., 2017), is the dominant mineral phase. In both cortical and trabecular bone, mineral platelets are highly organized around ordered collagen fibrils. In the vasculature, where calcification occurs at two anatomical sites—the vessel intima during atherosclerosis and the vessel media in aging, diabetes, and chronic kidney disease (CKD)—the mineral deposits are typically dystrophic, and a range of substrates can be calcified. These include collagen fibrils, though organized mineral akin to that in bone is rarely observed; striated elastin (Shimamura, 1970); and spherical nanostructures (Shimamura, 1970; Bertazzo et al., 2013; Hutcheson et al., 2016) that may originate from cell-derived extracellular vesicles (EVs) (Reynolds et al., 2004; Kapustin et al., 2015). Despite intensive study, it remains unclear how calcium ions are selectively and

This is an open access article under the CC BY license (http://creativecommons.org/licenses/by/4.0/).

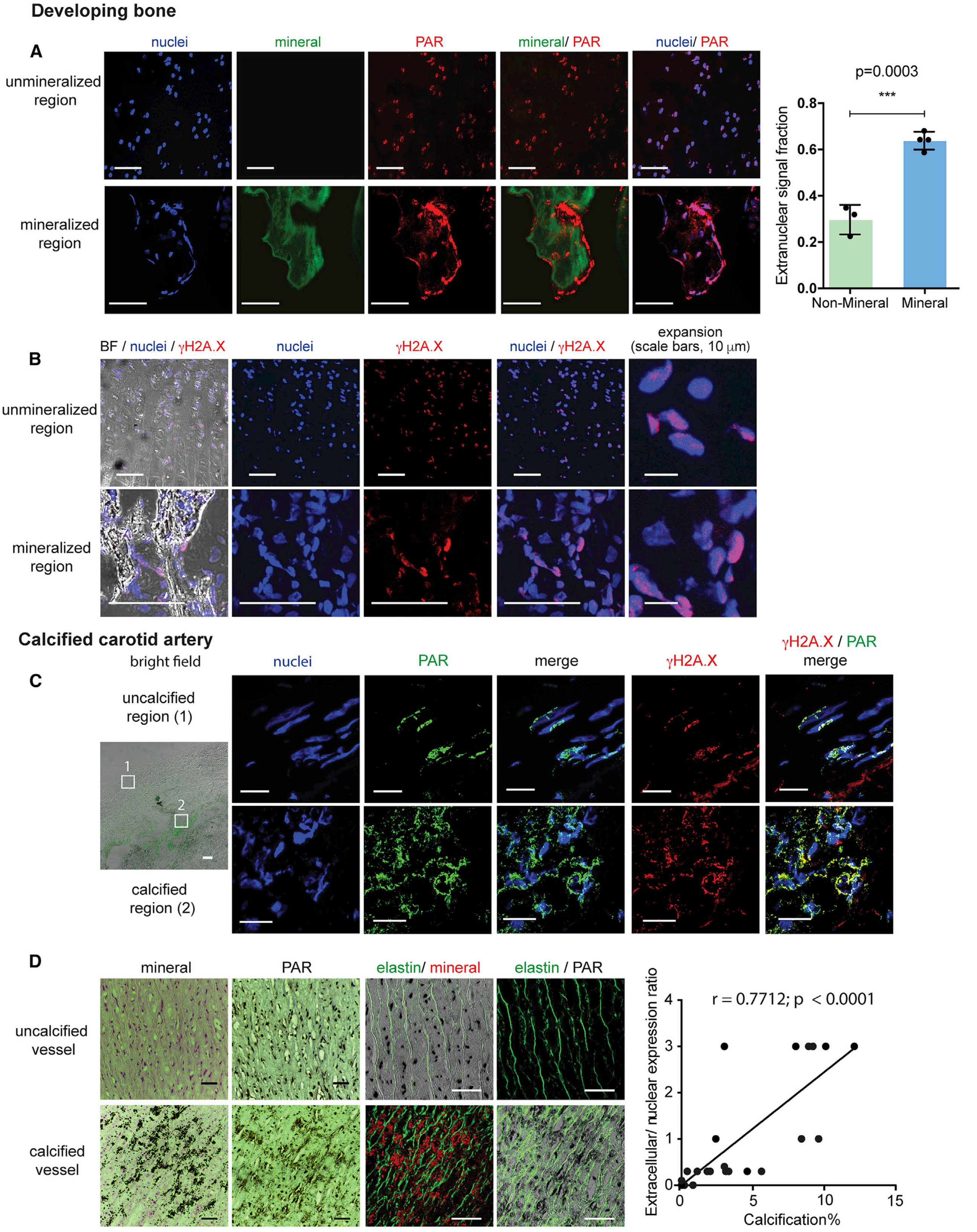

(legend on next page)

locally concentrated over other metal ions around these extracellular structures and why collagen fibrils are preferentially selected as nucleation sites.

Studies *in vitro* have shown that the formation of bone-like ordered mineral deposits around collagen fibrils requires other factors such as additional or substituting mineral ions or noncollagenous biomolecules (Nudelman et al., 2010; Wang et al., 2012, 2014). This implies that there is cellular control of ECM calcification through the secretion of specific factors, but the identification of these factors remains elusive. In both bone and the vasculature, biomineralization is accompanied by osteogenic differentiation of resident osteoblasts and vascular smooth muscle cells (VSMCs), respectively. Osteogenic differentiation results in increased expression of multifunctional acidic proteins, including the small integrin-binding ligand, N-linked glycoprotein (SIBLING) proteins, and speculation has focused on these "osteogenic" proteins as specialist molecules that may selectively bind calcium ions and provide specificity of interaction with collagen fibrils (Donley and Fitzpatrick, 1998; Bini et al., 1999; Wada et al., 1999; Iyemere et al., 2006; Jahnen-Dechent et al., 2008; Liu et al., 2013; Veis and Dorvee, 2013; Al-Qtaitat, 2014). However, although diverse signaling and inhibitory roles have been identified (Bini et al., 1999; Wada et al., 1999; Abedin et al., 2004; Vattikuti and Towler, 2004), these proteins do not have the calcium concentration capacity to induce collagen calcification. Hence, no physicochemical structural role in mineral formation has been conclusively demonstrated (Proudfoot et al., 2002; Boskey, 1989, 2007; Severson et al., 1995; Bini et al., 1999; Proudfoot and Shanahan, 2001; Moe et al., 2002; Veis and Dorvee, 2013).

Previously, using nuclear magnetic resonance (NMR) spectroscopy, we discovered that poly(ADP-ribose) (PAR) is abundant in the calcifying growth plate of developing fetal bone, which led us to hypothesize that PAR may play a role in biomineralization (Chow et al., 2014). PAR is a post-translational modification moiety composed of sugar phosphates that is produced by PAR polymerase (PARP) enzymes and adducted to numerous cellular proteins in a process known as PARylation. Several characteristics of PAR lend support to its possible extracellular role in biomineralization: first, the pyrophosphate groups of PAR are predicted to locally bind calcium ions, potentially to the levels needed for mineral nucleation. Second, PARP1 and PARP2, the dominant PAR-producing enzymes, are expressed in response to DNA damage and oxidative stress (Schreiber et al., 2006; Ba and Garg, 2011; Bai et al., 2015; Brunyanszki et al., 2016), both etiologies associated with vascular calcification. Third, emerging evidence suggests that osteogenic differentiation in calcifying osteoblasts is regulated by PARP activity induced by hydrogen peroxide release from cells (Robaszkiewicz et al., 2012). Therefore, we explored whether PAR could control the physicochemical process of mineral formation in the ECM and provide evidence that PAR biosynthesis, induced in part by the cellular DNA damage response (DDR), is a unifying factor in physiological bone and pathological artery calcification.

RESULTS

PAR Is Deposited at Sites of ECM Calcification in Close Apposition to Cells Exhibiting DNA Damage

Immunofluorescence (IF) was used to examine the juxtaposition of PAR, DNA damage, and mineralization in the fetal sheep growth plate and calcified human arteries. PAR deposition was observed in the acellular ECM abutting the calcification zone of bone trabeculae. In contrast, in non-calcified cellular regions (proliferation and/or hypertrophic pre-calcification zone), PAR was confined to cell nuclei (Figure 1A). Quantification of nuclear: non-nuclear PAR (Figures 1A and S1) gave 64% ± 3% as non-nuclear in the calcified trabeculae as compared with 30% ± 5% in the non-calcified regions. Nuclei of cells lining the bone trabeculae and in the proliferation and/or hypertrophic regions stained positive for histone H2AX phosphorylation, γH2AX (Figure 1B), a marker of DNA damage, consistent with nuclear PAR being synthesized as part of the DDR.

Similar deposition of extranuclear PAR was observed at sites of calcification in arteries within atherosclerotic plaques and the media, in contrast to non-calcified areas in diseased or normal arteries, where PAR was exclusively in cell nuclei. Cells in close proximity to calcification were positive for γH2AX, and spillage of chromatin into the ECM was indicative of cell necrosis at these sites (Figure 1C). Immunohistochemistry (IH) and quantification of PAR performed on a large series of non-calcified and calcified arteries, exhibiting either intimal atherosclerotic calcification (carotid arteries) or medial calcification (aorta), verified the pattern (Figure S1) of nuclear PAR in non-calcified vessels and/or areas and abundant extracellular deposition in areas with mineral. Reflectance mode microscopy demonstrated that the PAR in calcified aorta was located mostly between elastic lamellae of the vessel media (Figures 1B, 1D, and S1). Quantification of the ratio of nuclear versus extracellular PAR in human arteries showed a clear correlation between extracellular PAR deposition and calcification (Figure 1D).

Figure 1. Extranuclear PAR Correlates with Extracellular Matrix Calcification *In Vivo*

(A) Confocal images of unmineralized (proliferation zone) and mineralized fetal sheep growth plate taken from the same sample, showing the distribution of PAR with respect to mineral, with quantification of the fraction of PAR signal that is non-nuclear in a series of images. Graph shows mean ± SD. (See Figures S1A–S1C for image processing.)

(B) Confocal images from the same sample as in (A) showing the distribution of γH2A.X.

(C) Confocal images of human carotid artery taken from calcified and noncalcified regions of the same vessel, showing the distribution of PAR and γH2A.X. Images in (A)–(C) are maximum intensity projections of z stacks (see also Figures S1D and S1E).

(D) Immunohistochemistry of young uncalcified and aged calcified human vessels showing mineral and PAR staining. Quantification of extranuclear PAR and mineral in n = 23 vessels shows a correlation between the extent of extranuclear PAR and calcification in the vessel (see Table S1 for details). Correlation was performed using a Pearson Test (see also Figure S1F).

All scale bars 50 μm, unless otherwise indicated (i.e., in B).

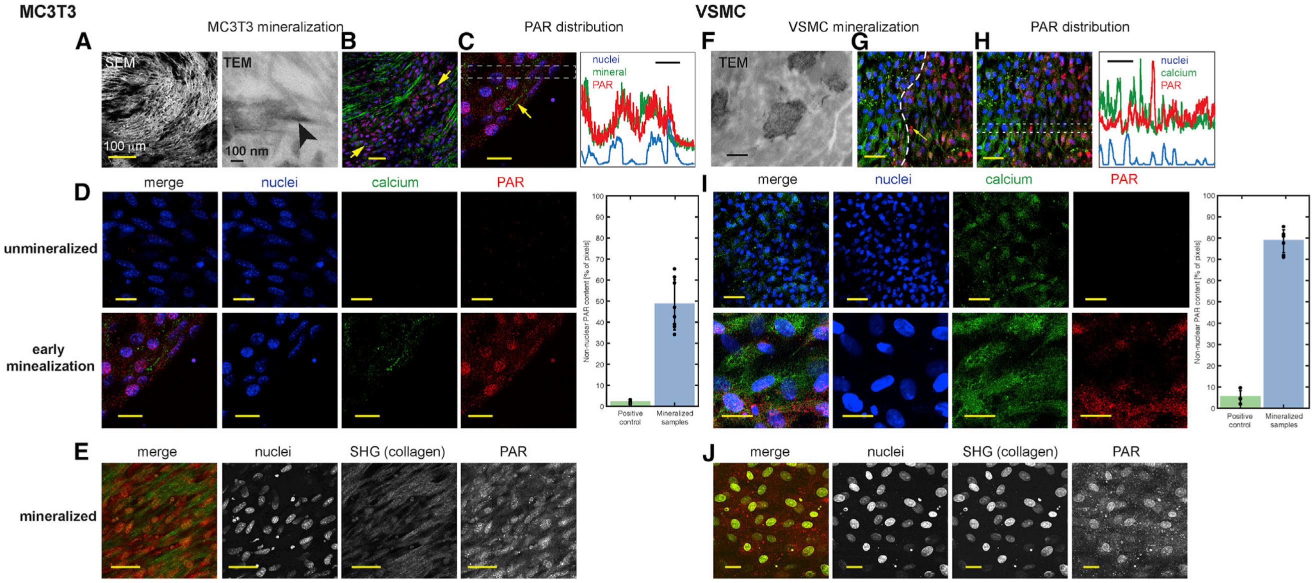

Figure 2. Calcification Correlates with PAR Synthesis *In Vitro*

(A–E) MC3T3 cell analysis.

(A) Mineral morphology and spatial distribution with respect to collagen fibrils observed by SEM (backscattered) and TEM. See also Figures S2A–S2D.

(B) Confocal image showing calcified collagen fibrils (green) and early-stage calcification area (yellow arrows).

(C) Higher magnification confocal image of an area of early-stage calcification and plot profile. PAR, red; nuclei, blue; calcium, green in (B) and (C).

(D) Color separations of confocal images for unmineralized MC3T3 matrix and the early-stage calcification area in (C) with quantification of extranuclear PAR in mineralized and positive control (H_2O_2-treated) samples. Graph shows mean ± SD. See Figure S2E for controls and Figure S2F for details of image quantification.

(E) Multiphoton images of an area of heavy matrix calcification, such as that in (B).

(F–J) VSMC cell analysis.

(F) Mineral morphology from TEM. Scale bar 500 nm. See also Figures S2G–S2J.

(G) Confocal image showing heavily calcified area (left, green) and earlier-stage calcification (right) containing PAR (red). See also Figure S2K.

(H) Plot profiles showing the distribution of PAR (red), nuclei (blue), and calcium (green).

(I) Color separations of confocal images for non-induced VSMC and mineralizing VSMC cultures with quantification of extranuclear PAR in mineralized and positive control (H_2O_2-treated) samples. Graph shows mean ± SD. See Figure S2L for controls and Figure S2M for details of image quantification.

(J) Multiphoton images of an area of heavy matrix calcification such as that in (G).

Scale bars 20 μm for (C)–(E), (I), and (J) and 50 μm for (B), (G), and (H).

PAR Is Deposited in the ECM of VSMCs and Osteoblasts in Response to Calcification Stimuli

Primary bovine VSMCs (bVSMCs) and MC3T3 E1 osteoblasts *in vitro* were used to model how induction of ECM calcification relates to PAR synthesis, ECM calcification being induced with β-glycerol phosphate (Mody et al., 2001; Kapustin et al., 2011; Robaszkiewicz et al., 2012; Addison et al., 2015).

Transmission electron microscopy (TEM) showed that MC3T3 osteoblasts lay down well-structured, highly ordered collagen fibrils (Figures 2A and S2D), and mineralization occurs exclusively as organized apatitic mineral platelets around these fibrils (Figures 2A, 2B, and S2A–S2D). Using IF early mineralization is characterized by diffuse mineral staining in cellular regions with an abundance of nuclei (yellow arrows in Figure 2B), while at later stages, there are few cell nuclei and heavily calcified collagen fibrils (Figures 2B and 2C).

PAR was barely detectable in cells prior to calcification (Figure 2D) but was strongly detectable in regions of early calcification (Figures 2B–2D). Plot profiles of confocal images show PAR co-localizing with mineral (Figure 2B) and, to a lesser extent, with nuclear DAPI, and quantification revealed 52% ± 12% of PAR was nuclear and 48% ± 12% non-nuclear (Figure 2D), as compared with (98% ± 1%) nuclear PAR in positive controls where PAR biosynthesis was induced with hydrogen peroxide (Figure S2E).

Mineral deposits scatter incident laser light in confocal imaging; therefore, to determine whether PAR was also present in or around heavily calcified collagen fibrils, we used multiphoton and second harmonic generation (SHG) imaging (Figure 2E). This showed the expected parallel arrays of collagen fibrils and considerable fluorescence intensity from PAR between the calcified collagen fibrils.

Under control conditions, bVSMCs produce well-structured collagen fibrils and elastin (Figure S2I) with no detectable mineral patches ("unmineralized" in Figure 2I) and essentially no observable PAR. In contrast, under calcifying conditions, no collagen was visible by TEM, SHG spectroscopy, or NMR spectroscopy (Figures 2F, S2G, and S2J), and regions containing numerous ovoid or circular patches of extracellular mineral (Figure 2F and left-hand side of Figure 2G; enlarged in Figure S2K), typically 1–5 μm in diameter, were observed. Regions adjacent to these calcified areas typically exhibited both nuclear and non-nuclear

OPEN ACCESS
CellPress

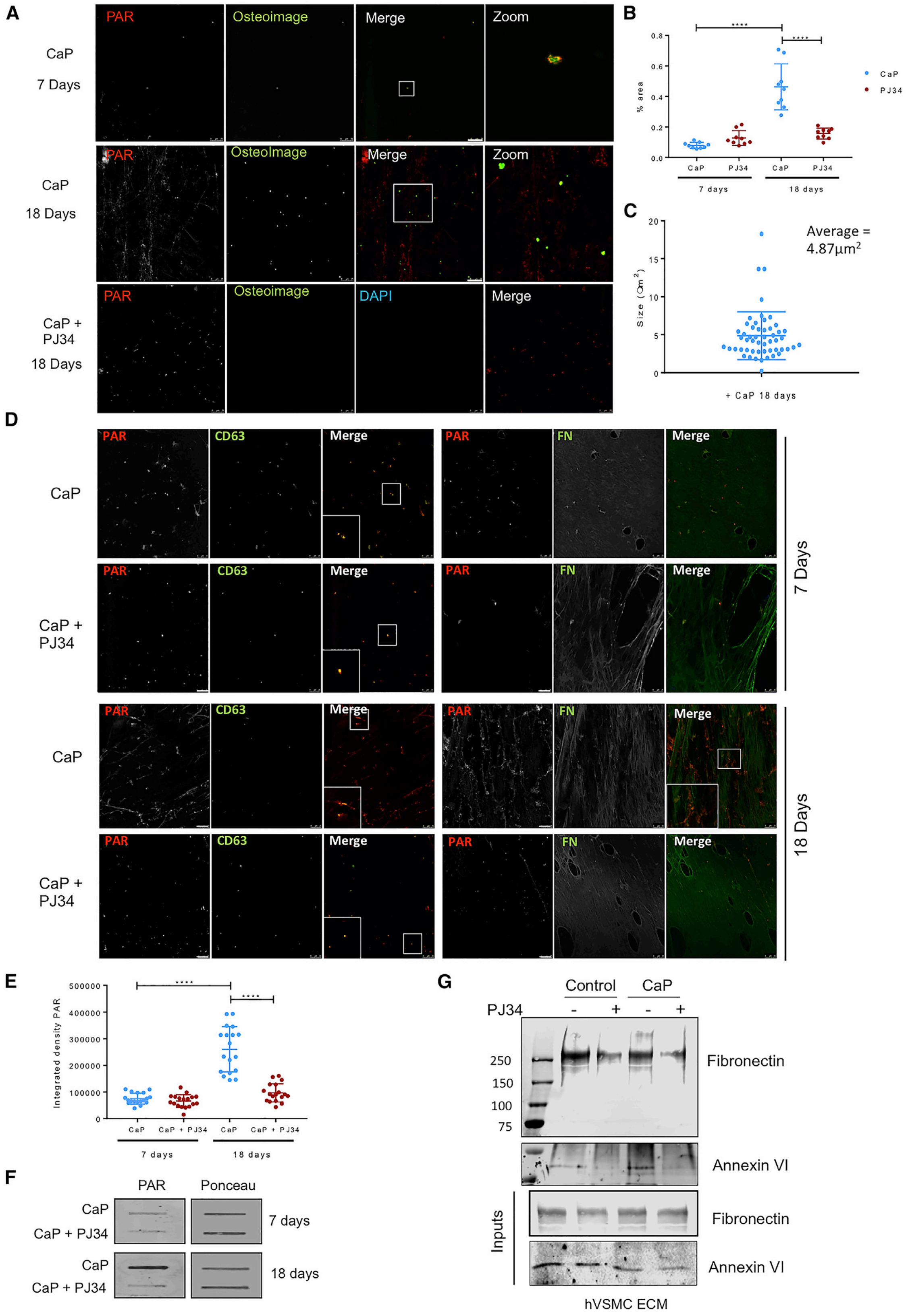

(legend on next page)

PAR (Figures 2G and 2I), mainly in small spherical structures less than a micron in diameter (Figures 2I and S2K), with some larger PAR-containing circular regions 2–6 μm in diameter (Figure S2K). Plot profiles of confocal signal intensities through calcified and adjacent regions showed (Figure 2H) PAR and calcium co-localization with widespread non-nuclear PAR in calcified regions. Quantifying the nuclear:non-nuclear PAR in calcified areas (Figure 2I) gave 78% ± 5% non-nuclear PAR compared to 5% ± 3% non-nuclear PAR in hydrogen peroxide-treated bVSMCs (Figure S2L).

PAR Binds to the ECM

As PAR is normally considered to be an intracellular moiety, we were interested to discover whether PAR can bind to the ECM. The bVSMCs that formed ECM were subjected to cell lysis, and the extracted ECM (Mody et al., 2001; Peiró et al., 2001; Johnson et al., 2006) was examined using 2D ^{13}C-^{13}C correlation NMR spectroscopy (Chow et al., 2014) (Figure S3A), which revealed significant quantities of PAR suggesting it can be "captured" by ECM components. The same spectra did not contain the distinctive deoxyribose C1′–C2′ correlation signal from DNA (Figure S3B), suggesting that all nuclear material had been removed from the ECM (Chow et al., 2014).

To visualize its deposition in the ECM, primary human VSMCs (hVSMCs) were induced to form a calcified matrix by high Ca^{2+} and inorganic phosphate concentrations modeling the dysregulated mineral metabolism observed in CKD. After 7 (pre-calcification) and 18 days (calcification), the cells were lysed and the remaining ECM washed extensively. DAPI staining confirmed the absence of DNA and/or extraneous nuclear material (Figures 3A and S3C). Fluorescence imaging confirmed that at day 7, matrix calcification was minimal and was in the form of a small number of spherical deposits (Figure 3A). At this time, PAR was deposited in the ECM in a punctate pattern co-localizing with the EV marker CD63 (Figure 3D), and the mineral spheres invariably co-localized with PAR puncta (Figure 3A). After 18 days, calcified spheres averaging 5 microns in size were spread throughout the ECM, and PAR deposition was more extensive (Figures 3A–3C). The punctate pattern of PAR co-localizing with CD63 remained visible, but in addition, there was now more extensive deposition in a fibrillar pattern that co-localized to a large extent with fibronectin (Figure 3D). Calcified spheres were interspersed on this fibrillar pattern of PAR staining (Figure 3A). Treatment with the PARP1/2 enzyme inhibitor PJ34 blocked calcification at day 18 and dramatically reduced PAR deposition in the ECM, although there was some residual PAR co-localizing with CD63 (Figure 3A). The use of a control immunoglobulin G (IgG) confirmed the specificity of the PAR antibody (Figure S3C). Calculation of the integrated density of PAR using ImageJ showed a significant increase at day 18 and its inhibition with PJ34 (Figure 3E). Protein slot blot analysis confirmed the presence of PAR in both the ECM and isolated EVs, the increase in ECM PAR deposition at 18 days, and its inhibition by PJ34 (Figures 3F and S3D).

To determine what components of the ECM might bind PAR, we analyzed publicly available databases of known PARylated proteins and identified fibronectin and annexins as proteins relevant in calcification and present in the ECM and VSMC-derived EVs (Shanahan et al., 2011; Kapustin et al., 2015). Using boronate beads that selectively bind PAR to isolate PARylated proteins from lysates of ECM and EVs and western blot, we found that fibronectin and annexin 6 were retained by the beads. Treatment of the VSMCs with the PARP inhibitor PJ34 reduced bead binding to fibronectin and annexin 6 in ECM and EV lysates (Figures 3G and S3E). Western blot of the input lysates showed equal levels of fibronectin and annexin 6, confirming PJ34 treatment had no effect on protein synthesis or secretion (Figure 3G).

PAR Forms Amorphous, Calcium-Rich Spheres with an Affinity for Collagen Fibril Hole Zones

We next explored the physicochemical properties of PAR to understand its role in the process of ECM calcification. We hypothesized that PAR acts by collecting calcium ions; thus, the first step was to determine the calcium-binding affinity of PAR compared with other biologically relevant divalent metal ions. Dynamic light scattering (DLS) was used to examine the calcium-binding properties of PAR in aqueous solution (Figure 4A), and bright-field (BF)-TEM, energy-dispersive X-ray spectroscopy (EDX), and single-area electron diffraction (SAED) were used to examine PAR morphology in the presence of calcium ions ($CaCl_2$) (Figures 4B and 4C).

DLS indicated that the PAR hydrodynamic radius increased dramatically with calcium chloride addition (Figures 4A and S4A). TEM images (Figures 4B and 4C) from solutions of PAR and calcium chloride deposited on grids showed electron-dense spheres. EDX (Figures 4B and S4) showed that the spheres contained calcium, as well as phosphorus consistent with PAR pyrophosphate groups, suggesting the spheres consisted of PAR-bound calcium ions. SAED revealed no diffraction spots or rings indicating that the material in these spheres was amorphous

Figure 3. PAR Is Released into the ECM via Cell Lysis and EVs and Binds ECM Proteins

(A) Immunofluorescence showing deposition of PAR and co-localization with OsteoImage in the ECM from hVSMCs treated in calcifying conditions for 7 and 18 days in the presence or absence of PJ34. Scale bar, 25 μm. See also Figures S3A–S3C.

(B) Quantification of mineralization in the ECM of hVSMCs induced to calcify in the presence or absence of PJ34 (n = 3). Mean ± SD, 2-way ANOVA, ****$p < 0.0001$. See also Figures S3D and S3E.

(C) Quantification of calcified particle size (OsteoImage spheres) at day 18 after induction of calcification (n = 3). Mean ± SD.

(D) Immunofluorescence showing deposition of PAR and co-localization with CD63 and fibronectin in the ECM from hVSMCs treated in calcifying conditions for 7 and 18 days in the presence or absence of PJ34.

(E) Quantification of PAR deposition in the ECM in calcifying conditions in the presence and absence of PJ34 (n = 3). Mean ± SD, 2-way ANOVA, ****$p < 0.0001$.

(F) Protein slot blot showing increased PAR production in calcifying hVSMCs (CaP) and its inhibition by PJ34 (representative of n = 2 experiments).

(G) Boronate bead binding assay and western blot for fibronectin and annexin 6 showing reduced binding to PAR in the ECM of hVSMCs treated with PJ34 (representative of n = 2 experiments). Input shows equal loading onto beads.

OPEN ACCESS
CellPress

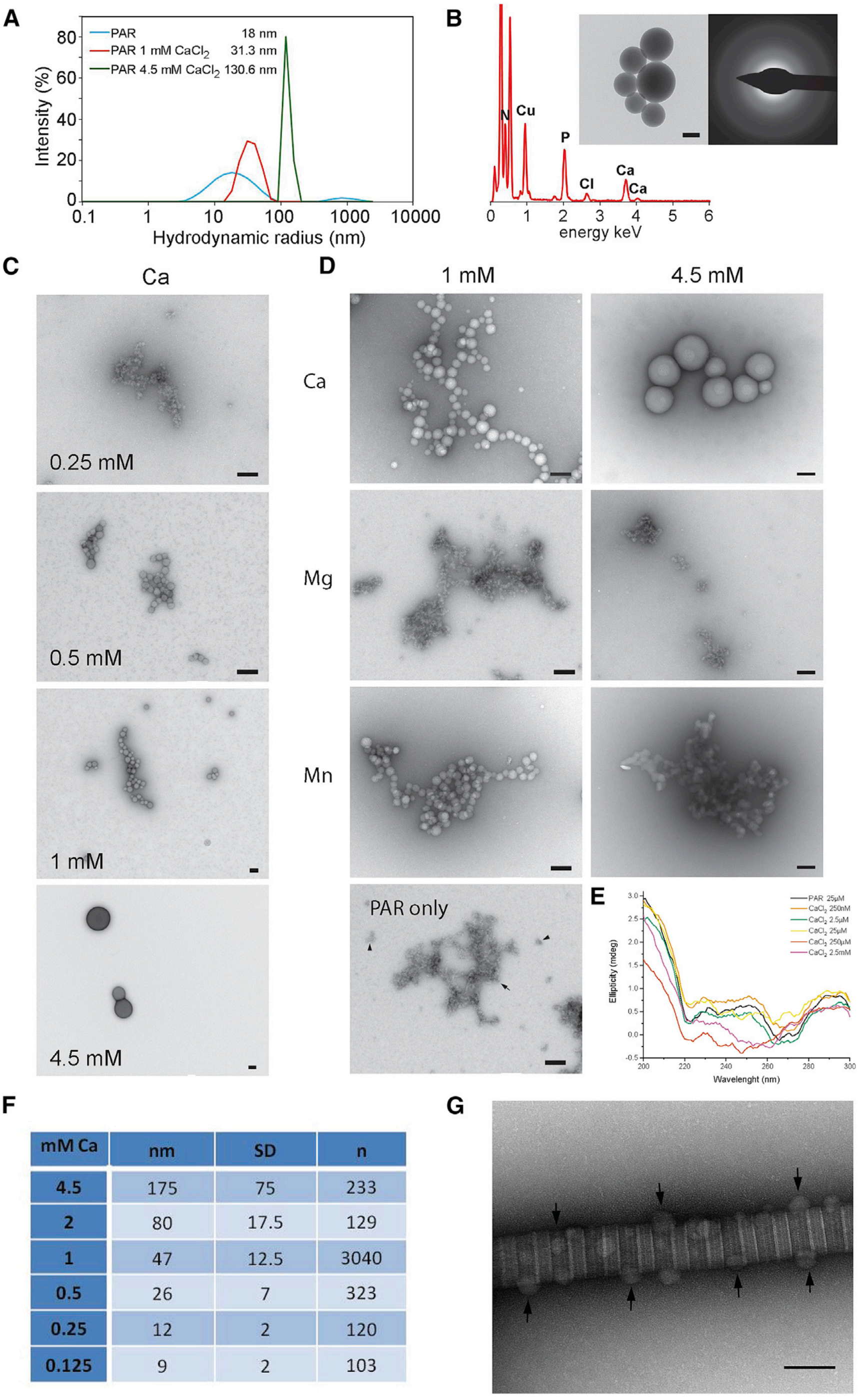

mM Ca	nm	SD	n
4.5	175	75	233
2	80	17.5	129
1	47	12.5	3040
0.5	26	7	323
0.25	12	2	120
0.125	9	2	103

Figure 4. PAR Has Considerable Ca^{2+} Binding Capacity

(A) Hydrodynamic radius of PAR in the presence of Ca^{2+} measured by DLS. See also Figure S4A.

(B) EDX and SAED (inset, right) of PAR-Ca spheres observed by TEM (inset, left). See Figure S4B for details of analysis.

(C) PAR-Ca spheres increase in diameter with Ca^{2+} concentration.

(D) Comparison of the morphology of PAR in the presence of Ca^{2+} compared with other divalent metal ions and PAR alone. Scale bars, 100 nm for (B)–(D). See also Figure S4C.

(E) CD spectra of PAR + Ca^{2+} as a function of Ca^{2+} concentration.

(F) PAR-Ca sphere diameter as a function of Ca^{2+} concentration. See Figure S4B for details of analysis.

(G) Bright-field (BF)-TEM of collagen fibrils incubated with PAR in the presence of calcium ions (0.5 mM $CaCl_2$) showed that calcium-rich PAR spheres were found almost exclusively bound to the surface of the collagen fibrils preferentially at the hole zones (arrows). See Figure S4D for details of analysis.

(Figure 4B, right inset). The diameter of the spheres depended on Ca^{2+} concentration as assessed from TEM images (Figures 4E and S4B), and PAR molecular conformation as assessed by circular dichroism (CD) spectra (Figure 4E) also varied with Ca^{2+} concentration, suggesting that PAR has a large Ca^{2+} binding capacity.

In contrast to the case for Ca^{2+} ions, PAR showed almost no affinity for other divalent metal ions (Mg^{2+} and Zn^{2+}), except for Mn^{2+} (Figures 4D and S4C), which also produced electron-dense spheres (Figure S4). However, the radius of the Mn-PAR spheres did not increase in size when the Mn^{2+} concentration was increased from 1 mM to 4.5 mM. This suggests that at 1 mM Mn^{2+}, the PAR Mn-binding capacity is already saturated, and PAR has lower affinity for Mn^{2+} than for Ca^{2+}. As a control, we examined Ca^{2+} binding properties of DNA, as a polynucleotide analogous to PAR (Figure S4C), but TEM imaging showed no spheroidal structure formation with Ca^{2+}.

We then used TEM to examine whether PAR-Ca^{2+} spheres can interact with collagen fibrils when introduced to the PAR/$CaCl_2$ buffered solution. The TEM images show that PAR-Ca spheres avidly bound to collagen fibril surfaces (Figure 4F). The PAR-Ca^{2+} spheres showed a significant preference (86% ± 6%; Figure S4D) for binding to the collagen fibril hole zones (Figure 4F, arrows), the region hypothesized to be the site of initial mineral nucleation (Glimcher, 1984; Weiner and Traub, 1986; Landis et al., 1993; Mahamid et al., 2010).

PAR Generates Bone-like Calcification of Collagen Fibrils

We next asked whether the PAR-Ca^{2+} spheres could, in the presence of inorganic phosphate, induce bone-like calcification of collagen fibrils (Reznikov et al., 2018). Addition of PAR to

OPEN ACCESS
CellPress

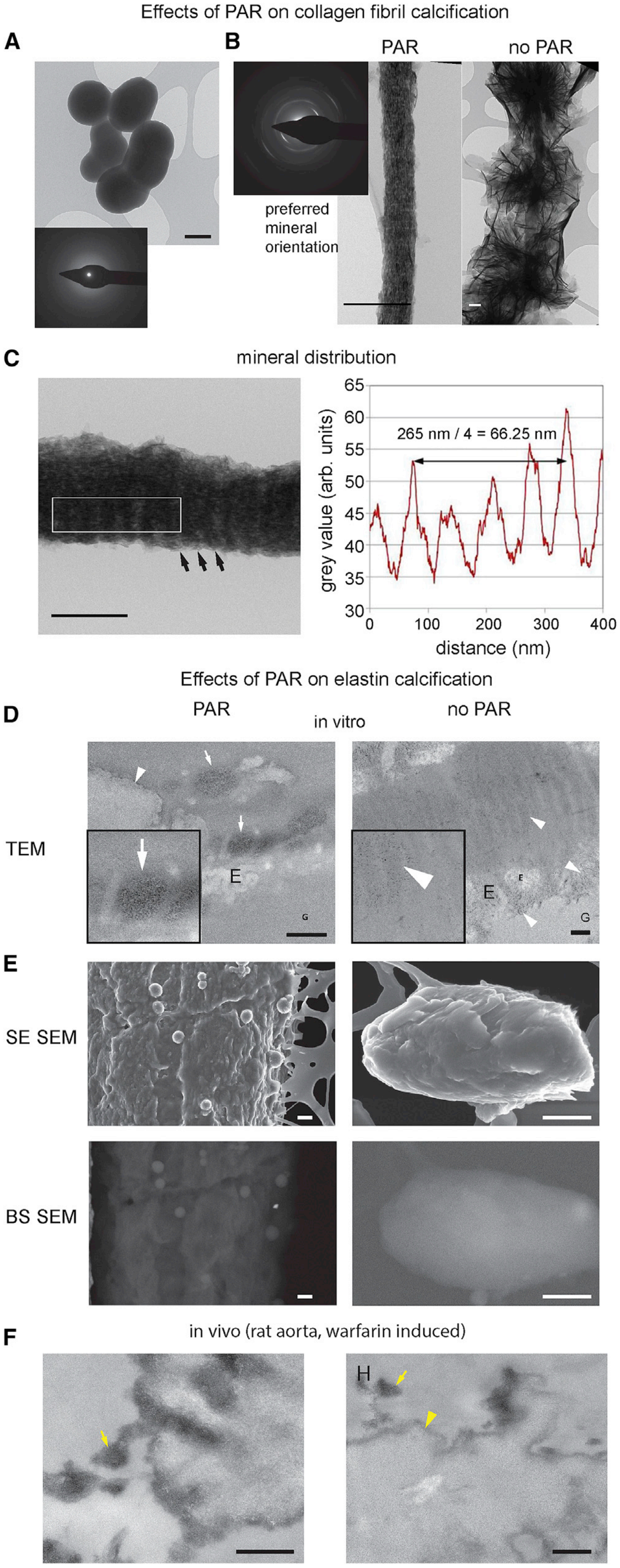

Figure 5. PAR Can Induce Calcification of Extracellular Matrix Components

(A) TEM image of PAR with 4.5 mM Ca^{2+} ($CaCl_2$), 2.1 mM PO_4^{3-} (K_2HPO_4) TRIS buffered solution showing spheres, similar to the calcium-rich PAR spheres seen after incubation with $CaCl_2$ alone. Scale bar, 100 nm.

(B) TEM images of the mineral resulting from co-incubation of collagen fibrils in the presence (left) or absence (right) of PAR *in vitro* with a buffered solution containing 4.5 mM Ca^{2+} and 2.1 mM phosphate for 14 days. Scale bars, 250 nm. See also Figure S5A.

(C) Higher TEM magnification (left) showing the periodicity (arrows) of the mineral deposition in the PAR-calcified collagen fibrils (unstained). Density plot (right) taken from the boxed area shows the distance between maxima was ~66 nm.

(D and E) TEM (D) and SEM (E) of resin thin section of elastin + PAR and elastin alone incubated in Ca^{2+} and PO_4^{3-} solutions for 14 days. White arrowheads in TEM images indicate fine, electron-dense material; white arrows denote larger areas of electron-dense material. G, gelatine; E, elastin. Secondary electron (SE) SEM images of elastin + PAR samples to assess the surface topography of elastin after incubation show numerous spheres on elastin surfaces, and elastin alone does not. Backscattered (BS) images show electron-dense areas as lighter, and they show the elastin + PAR sample spheres are electron dense and that the surface of elastin-alone samples is covered with diffuse, electron-dense material. See Figure S5B for controls and Figure S5C for more images.

(F) Block-face SEM images of rat medial aorta calcification induced by warfarin diet. Yellow arrows indicate areas of electron-dense material.

Scale bars, 500 nm for (D)–(F).

buffered calcium phosphate solution (4.5 mM $CaCl_2$, 2.1 mM K_2HPO_4) alone, without collagen fibrils, resulted in formation of amorphous, calcium-rich, electron-dense spheres (Figure 5A), highly similar in appearance to those formed with $CaCl_2$ and PAR, again with diameters dependent on Ca^{2+} concentration. Of note, the diameters of these PAR-Ca phosphate spheres were in the range of the spheroidal mineral deposits found in our *in vitro* models, which are also described in calcified vascular tissues *in vivo* (100 nm – few microns) (Shimamura, 1970; Bertazzo et al., 2013; Hutcheson et al., 2016). By SAED (Figure 5A, inset), these PAR-induced spheres were still amorphous even after incubation for 14 days in calcium-phosphate rich buffer, suggesting that they are stable in the absence of collagen fibrils or other perturbing influence for some considerable period.

The introduction of collagen fibrils to PAR in the same buffered calcium phosphate solution induced heavy mineralization of the fibrils after 14 days, with numerous nanoscopic mineral platelets aligned along the fibrils (Figure 5B, left) in an ordered manner reminiscent of bone mineral (Shimamura, 1970; McNally et al., 2012; Nudelman et al., 2012). This ordered platelet alignment was confirmed by SAED, which showed distinct arcs consistent with crystals with preferred orientation (Figure 5B). At a higher magnification, distinct zonation of mineral deposition with respect to the collagen fibril structure was visible (Figure 5C). A density plot revealed the distance between mineral density maxima to be ~66 nm, in close agreement with the length of the D-period of collagen fibrils, suggesting that mineral nucleation and growth depend on the underlying collagen fibril structure. At intermediate time points, Ca-PAR spheres interacting with partially calcified collagen fibrils were observed (Figure S5A), suggesting these spheres could be directly involved in collagen fibril calcification. Consistent with this notion,

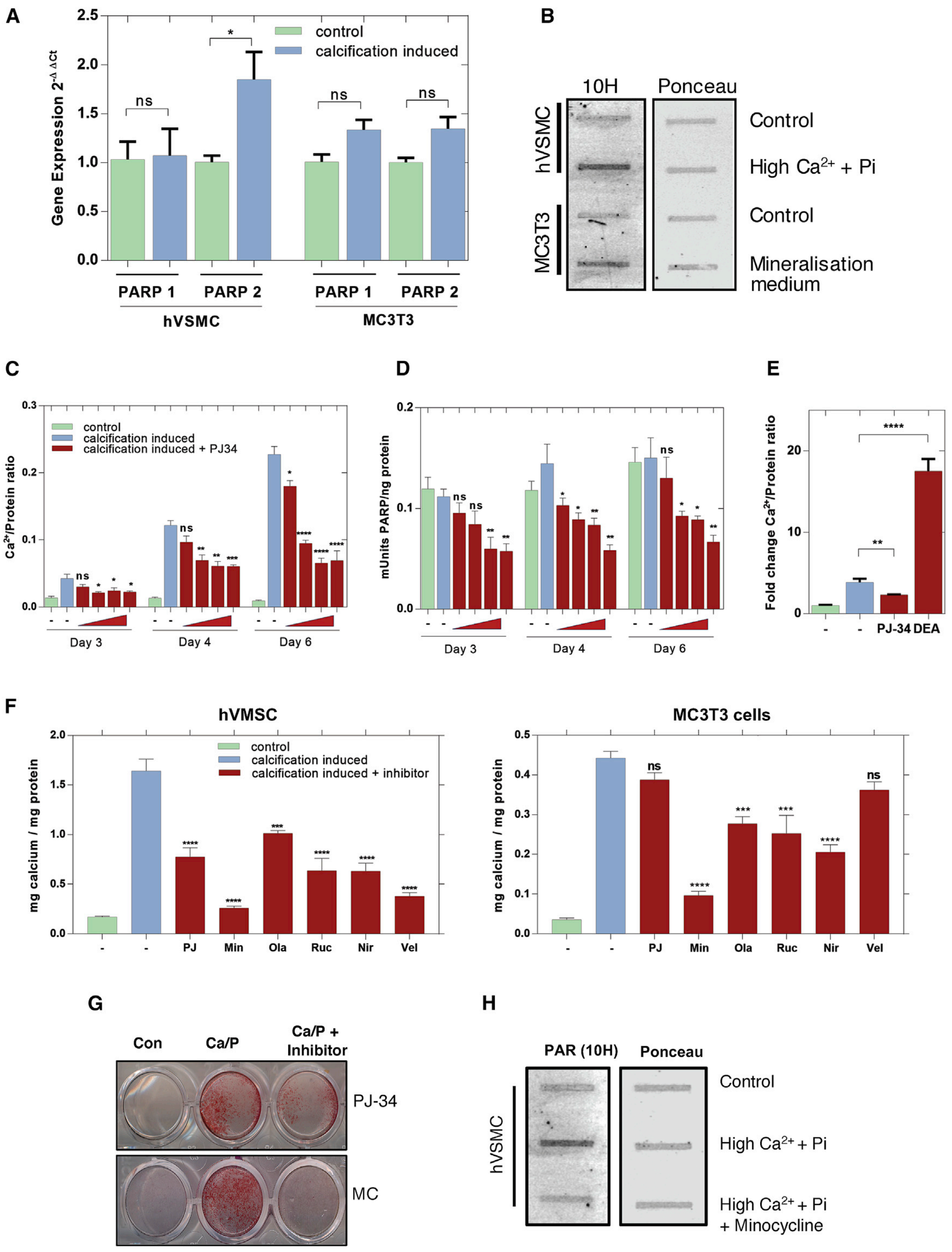

Figure 6. PARP Inhibitors Decrease Calcification of hVSMCs *In Vitro*

(A) qRT-PCR analysis of hVSMCs and MC3T3 cells *in vitro* shows expression of PARP1 and PARP2 enzymes in control cultures. hVSMC cultures under calcifying conditions show increased expression of PARP2 (n = 3).

(B) Slot blot of hVSMC and MC3T3 cells under calcifying conditions showing increased PAR.

(C) The o-cresolphthalein assay (n = 3) showed a time-dependent increase in mineralization, which was inhibited in a dose-dependent manner by the PARP inhibitor PJ-34 (0.5, 1.5, and 10 uM).

(legend continued on next page)

collagen fibrils in buffered calcium phosphate without PAR (Figure 5B, right) showed calcification more typical of a "wet precipitate," consistent with previous observations (Nudelman et al., 2010). The mineral crystals that did form in the PAR-free solution were randomly nucleated along the collagen fibril and not aligned with the fibril.

We also tested the affinity and calcification potential for PAR-Ca^{2+} droplets on elastin (Shimamura, 1970). Elastin was calcified *in vitro* in the buffered calcium phosphate solution as before. When PAR was present, mineral formed on elastin fragments in irregularly shaped patches (Figure 5D), highly similar to the mineral morphology found *in vitro* in bVSMC calcified ECM (Figures 2F and 2G) and *in vivo* around elastin fibers at the earliest stages of mineralization in a rat model of aortic calcification (Figure 5F) (Price et al., 1998; Gourgas et al., 2018). We also frequently observed what appeared to be Ca-PAR spheres bound to elastin surfaces (Figure 5E). In the absence of PAR, like collagen, the elastin surface was calcified with wet precipitate only (Figures 5C and S5C).

PARP Inhibitors Inhibit ECM Mineralization *In Vitro*

To examine the biochemistry of PAR synthesis, qRT-PCR was used to show that hVSMCs and MC3T3 cells *in vitro* expressed PARP1 and PARP2 under control conditions and increased expression under calcification conditions, with VSMCs selectively increasing PARP2 (Figure 6A). Increased PAR synthesis under calcifying conditions was confirmed by protein slot blot analysis (Figure 6B).

The non-selective PARP1/2 inhibitor PJ34 reduced PARP activity and inhibited calcification in both human and bVSMCs (Figures 6C, 6D, and S6) (Boström et al., 1993). Conversely, the PAR glycohydrolase (PARG) inhibitor 6,9-diamino-2-ethoxyacridine-DL-lactate (DEA), which acts to inhibit PAR cleavage and breakdown, led to an increase in mineral deposition (Figure 6E).

Next, we carried out PARP1 and PARP2 enzyme activity inhibition assays for a larger number of PARP inhibitors to establish their PARP1/2 selectivity (Table S2) and tested these in the *in vitro* calcification models. This demonstrated that all inhibitors were able to reduce calcification of hVSMCs and MC3T3 osteoblasts (Figure 6F). Of note, minocycline was the most effective PARP inhibitor for reducing calcification of hVSMCs, and protein slot blot analysis confirmed that minocycline could reduce PAR production (Figures 6G and 6H). Minocycline has previously been demonstrated to inhibit PARP1 (Alano et al., 2006), but its selectivity was not tested. The enzyme activity assays we performed showed that minocycline (Figure S6C; Table S2) inhibited PARP2 at much lower concentrations than those that provide effective PARP1 inhibition, suggesting that minocycline is acting as a selective PARP2 inhibitor.

A PARP Inhibitor Can Inhibit Biomineralization *In Vivo* in a Rat CKD Model

The efficacy of minocycline to reduce VSMC calcification *in vitro* led us to test this inhibitor *in vivo*. Rats fed a high-adenine and low-protein diet developed CKD, hyperphosphatemia, and medial vascular calcification (Diwan et al., 2018). Treatment with doses of minocycline from 5 to 50 mg/kg had no effect on parameters of renal failure including serum P, Ca, and creatinine clearance, confirming that any effects on calcification are likely to be tissue specific (Figure S7). Calcium quantification showed that the highest minocycline dose of 50 mg/kg significantly inhibited calcification in the aorta, femoral, and carotid arteries (Figure 7A). This was consistent with von Kossa staining of aortic sections showing a significant reduction in the calcified aortic area in the minocycline-treated group (Figure 7C), with some animals completely devoid of calcification (Figure 7B, boxes c1–c4). CKD was associated with the induction of DNA damage (Shanahan et al., 2011; Liu et al., 2013) (Figures 7B and 7C), shown by a significant increase in γH2AX staining of VSMCs in the aorta (Figure 7D); DNA damage likely preceded calcification as it was also elevated in minocycline-treated CKD non-calcified aorta (Figure 7E). Minocycline reduced the levels of γH2AX staining (Figures 7B and 7D) and maintained vascular integrity and VSMC number when compared with CKD alone (Figures 7B and 7F). PAR was detectable in VSMC nuclei in control and CKD animals; however, deposition in the ECM was only visible in the calcified arteries of CKD rats. PAR ECM deposition was less evident in rats treated with minocycline, consistent with the reduced cell death and ECM calcification observed (Figure 7B, boxes d1–d4).

The high-adenine and low-protein rat model also causes increased rates of bone turnover (Diwan et al., 2018); therefore, we examined the effects of minocycline on bone mineralization. SEM images of cross sections of long limb bone showed a larger fraction of calcified tissue in the CKD rats compared to controls. Minocycline-treated CKD rats showed reduced cortical thickness and areas of reduced mineral density, consistent with inhibition of calcification during bone remodeling (Figures 7G and 7H). High-resolution TEM imaging showed that the mineral deposition around collagen fibrils in control, CKD, and minocycline-treated CKD rats is similar in morphology and extent. This suggests that CKD does not interfere with the mechanism by which extracellular mineral is deposited, only the rate of bone remodeling with PARP inhibition reducing the amount of mineral deposited.

(D) PARP activity was reduced by PJ34 treatment in a dose-dependent manner (n = 4) in hVSMC cultures. See Figures S6A and S6B for data on bVSMC model.

(E) The PARG inhibitor DEA (0.1 mM) increased calcification of hVSMCs treated with high Ca/P media quantified by o-cresolphthalein. (n = 6). Mean ± SEM, Student's t test, **p < 0.01, ****p < 0.0001.

(F) PARP inhibitors can block calcification of hVSMCs (n = 3) and MC3T3 (n = 6) cells. PJ = PJ-34, Min, minocycline; Ola, Olaparib; Ruc, Rucaparib; Nir, Niraparib; Vel, Veliparib; all are at 3 μM. See Table S2 for enzyme inhibitor assay details and Figure S6C for details on minocycline as a PARP1/2 inhibitor.

Graphs in (A), (C), (D), and (F) show mean ± SEM. Statistical significance was tested with one-way ANOVA with Dunnett's post hoc tests. *p between p < 0.05, **p < 0.01, ****p < 0.0001.

(G) Alizarin Red S staining of mineral in hVSMC cultures in the absence and presence of PARP inhibitors PJ-34 (3 μM) and minocycline (3 μM).

(H) Slot blot showing increased PAR under calcifying conditions and its inhibition by minocycline (3 μM).

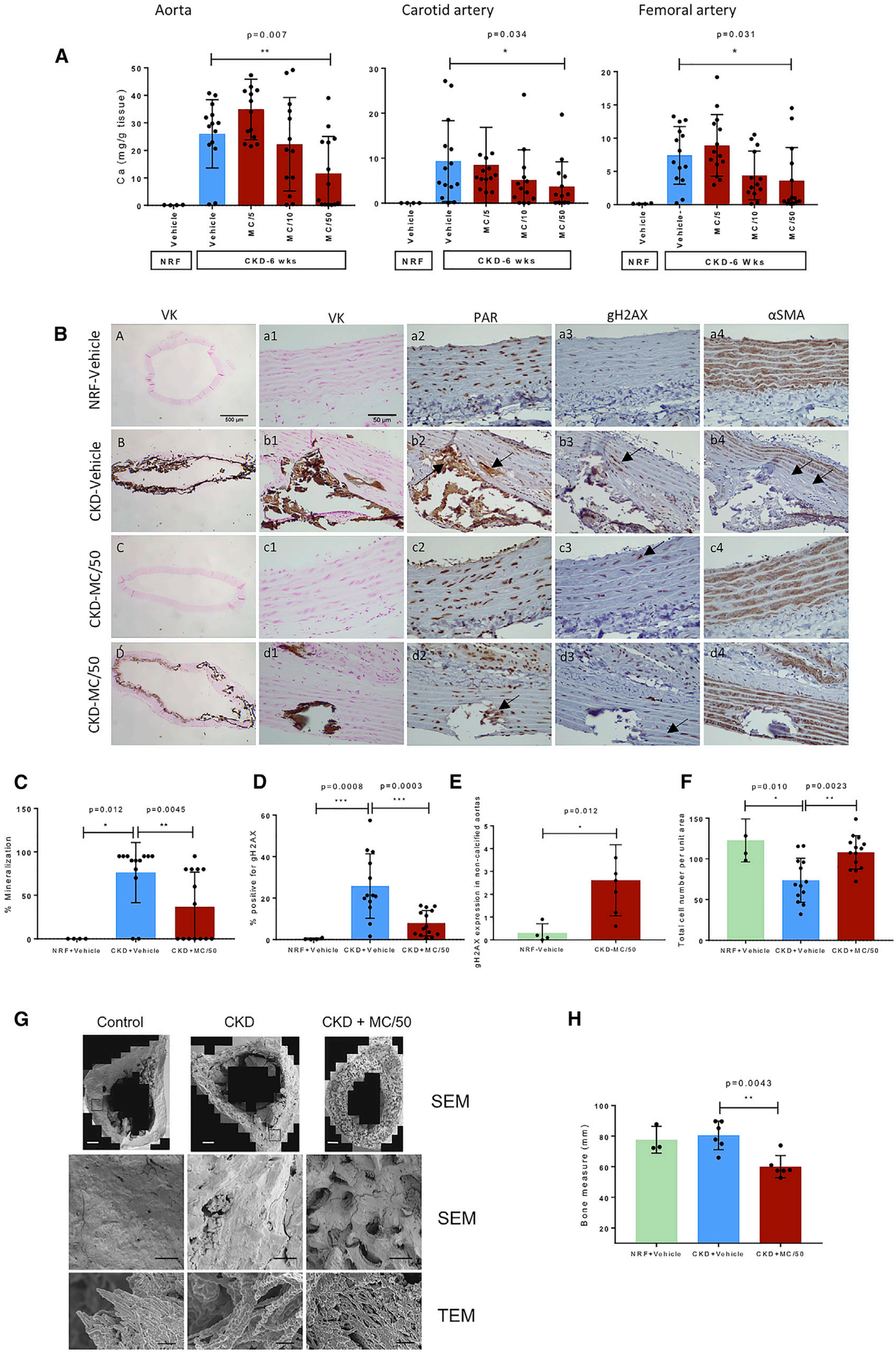

(legend on next page)

DISCUSSION

In this study, we show that PAR is present in the ECM of both physiological developing bone and pathological VSMC calcification *in vitro* and *in vivo* and that inhibitors of PARP enzymatic activity reduce mineralization. Biologically, PAR synthesis correlated with oxidative stress and DNA damage signaling—key pathways implicated in both bone and vascular calcification. PAR export from the cell, via cell death and EVs, is also consistent with initiating events at sites of mineralization, particularly in the vasculature.

We also show that PAR has the chemical functionality to concentrate calcium ions selectively over other divalent metal ions and can biomimetically mineralize both collagen and elastin, and it has particular avidity for collagen fibrils. Therefore, in bone where the initial organic matrix consists almost entirely of collagen fibrils, the presence of PAR ensures that the mineral forms in a highly organized arrangement on collagen fibrils and not in the disordered, random manner that occurs when collagen fibrils are exposed to calcium and inorganic phosphate alone. In the vasculature, where there are both degraded collagen fibrils (Hutcheson et al., 2016) and elastin fibrils and PAR-containing spheres and/or vesicles in the ECM, PAR can explain the heterogeneity in calcification sites. Taken together, these data provide a unifying mechanism for the initiation of both physiological and pathological mineralization, both explaining the physicochemical mechanism of matrix calcification and intriguingly suggesting that the DDR plays a key role in controlling ECM calcification.

PAR Chemistry Explains the Affinity and Periodicity of Collagen Mineralization

In developing zebrafish bones, calcium phosphate is delivered to the mineralization front as amorphous globules that then mineralize the bone collagen matrix, though what drives the formation of these calcium phosphate-rich globules is not known (Mahamid et al., 2010). Others have demonstrated that collagen fibrils calcify biomimetically in cell-free experiments in the presence of the polyanion poly(Asp) through a mechanism where the polyanion sequesters calcium and phosphate ions into amorphous liquid droplets (a polymer-induced liquid precursor [PILP] phase) (Olszta et al., 2007) and complexes of pre-nucleation calcium phosphate clusters. Importantly, the diameter of these structures (70–90 nm) is similar in size to those we find with PAR (Nudelman et al., 2010). By analogy, the PAR-Ca^{2+} spheres we observed would most likely form through spatial constraint of Ca^{2+} ions by interaction with PAR pyrophosphate groups, sustaining locally high calcium ion concentrations. In the absence of collagen or other ECM solid nanostructures, these PAR-induced PILP droplets could lead to formation of spherical mineral particles, consistent with the observation of such particle morphologies in collagen-depleted regions of vessels and between collagen fibers (Bertazzo et al., 2013; Hutcheson et al., 2016). In contrast, where there are collagen fibrils, the droplets are directed to interact preferentially with fibril hole zones initiating extensive fibril calcification. We speculate that the preferential calcification of collagen fibrils is due to the high extent of interaction possible between collagen fibrils and PAR. We do not yet know the nature of the PAR-collagen interaction at the molecular level. It may be due to electrostatic interaction between the negatively charged PAR polyanion with the collagen positively charged C-terminal, which is located in the collagen fibril hole zone (Nudelman et al., 2010), or more sequence-specific interactions between PAR functional groups and those on collagen fibrils; the stacked Tyr residues of the collagen C-terminal is a potential PAR-binding site (Eustermann et al., 2010).

PAR has also previously been shown to form liquid droplets with intrinsically disordered proteins (Altmeyer et al., 2015; Boskey and Villarreal-Ramirez, 2016), and many of the non-collagenous acidic osteogenic proteins essential for both bone and vascular calcification are intrinsically disordered. Addition of a representative acidic osteogenic protein to the poly(Asp)-collagen calcifying system resulted in more rapid biomimetic collagen fibril calcification, suggesting the rate of mineralization was dependent on the osteogenic protein (Nudelman et al., 2012). We speculate that complexes or the interplay between PAR and osteogenic proteins serve to control the rate and density of calcification, and this requires further exploration.

PAR Delivery to the ECM may be Mediated by Cell Death and Microvesicle Release

We have demonstrated that PAR can bind to the ECM in *in vitro* models of VSMC and osteoblast calcification with cell lysis likely mimicking cell necrosis, suggesting this may be one route for PAR delivery to the ECM. We also found a spatial correlation

Figure 7. The PARP2 Inhibitor Minocycline Inhibits Biomineralization *In Vivo*

(A) Quantification of calcium content in rat arteries under control (n = 4), CKD (n = 14), and CKD minocycline-treated rats (n = 14). Calcium is significantly reduced in the 50 mg/kg treated group. See Figures S7A and S7B for overview and background data for the *in vivo* experiments.

(B) Immunohistochemistry of rat aorta from control, CKD, and CKD minocycline-treated rats showing mineral (VK), PAR, DNA damage (γH2AX), and VSMCs (alpha smooth muscle [aSM] actin). Arrows in row b indicate areas of extracellular PAR, and arrows in row c indicate cells positive for γH2AX. Scale bar, 500 μm.

(C) Quantification of the mineralized area of the aorta in control (n = 4), CKD (n = 14), and CKD minocycline-treated (n = 14) rats.

(D) Quantification of γH2AX staining in the rat aorta from control (n = 4), CKD (n = 14), and CKD minocycline-treated rats (n = 14).

(E) Quantification of DNA damage in control (n = 4) and uncalcified arteries from the CKD minocycline-treated group (n = 6).

(F) Quantification of the total number of smooth muscle cells per unit area in the aorta control (n = 4), CKD (n = 14), and CKD minocycline-treated (n = 14) rats.

(G) Electron microscopy assessment of bones from the treated animals. (top) SEM images (scale bars, 500 μm), below expansions of the indicated areas of the SEM images (scale bars, 100 μm), and (bottom) TEM images showing details of the collagen fibril calcification (scale bars, 500 nm).

(H) Effect of minocycline on bone remodeling. The area fraction (in %) of "solid bone" in the cortical area of the bone cross section is decreased in minocycline-treated CKD animals.

All data were tested for normality using Shapiro-Wilk test. (A, C, D, F, and H) Mean ± SEM. Statistical significance was determined by Kurskall-Wallis test followed by Mann-Whitney test. *$p < 0.05$, **$p < 0.01$, ***$p < 0.001$. (E) Mean ± SEM. Statistical significance was determined by unpaired Student's t test, *$p < 0.05$. See Figure S7C for details of the quantification.

between CD63, a marker of VSMC EVs, and PAR in the ECM, suggesting PAR could also potentially deposit in the ECM via exosome or apoptotic body release. Both fibronectin and annexin 6 are well documented to be present in the ECM and VSMC-derived EVs (Kapustin et al., 2011, 2015). Annexin 6 was previously found to be PARylated or associated with PAR in oxidatively stressed cells (Jungmichel et al., 2013), and we confirmed this in VSMC ECM and EV lysates. Mechanisms of annexin-mediated mineralization remain controversial, but it is plausible that PAR binding may be involved in nucleating the mineral around EVs to generate spherical calcified particles (Shimamura, 1970; Becker et al., 2004; Bertazzo et al., 2013; Hutcheson et al., 2016).

PARP Activity Mediated by Oxidative and DNA Damage Induces the Production of PAR at Sites of Calcification

PARylation of proteins by PARP1/2 is a canonical pathway in the DDR, activated in particular by oxidative stress (Luo and Kraus, 2012). Consistent with this, we showed that γH2A.X was present in cells surrounding sites of mineralization in developing bone and calcified vessels. The notion of the DDR or cell damage signaling being essential in matrix calcification is further supported by previous studies. For example, mice deficient in key DDR signaling molecules including ataxia telangiectasia mutated (ATM) and p38 (Wang and Li, 2007) have defects in bone differentiation and mineralization, and similarly, ATM inhibition can block mineralization of aged VSMCs *in vitro* (Liu et al., 2013). Previous observations that PARP inhibition has additional effects in blocking cell necrosis and osteogenic differentiation suggests there may be additional roles for the DDR signaling cascade in mineralization (Robaszkiewicz et al., 2012). The notion that PARP signaling activated during the DDR, in response to either programmed (in bone) or pathological (in the vasculature) oxidative stress, can regulate osteoblast and VSMC osteogenic differentiation and cell death, as well as generate a molecule, PAR, that is essential to the physicochemical process of ECM calcification, is ingenious (Chen et al., 2015; Li et al., 2009). That the mechanism uses components that are considered some of the most ancient biological molecules—ribose phosphate and adenine (as well as calcium and inorganic phosphate)—suggests that the mechanism itself may also have ancient roots.

Most importantly, our data *in vivo* using a rat CKD model of vascular calcification suggest that PARP inhibitors may be developed as therapeutics for the widespread condition of vascular calcification, for which there is currently no treatment. Development of new PARP inhibitors is currently a growth area in cancer treatment (Alano et al., 2006; Lee et al., 2007; Kim et al., 2013), and understanding which PARP enzymes are responsible for bone and vascular calcification would potentially allow treatment developments for the specific inhibition of vascular calcification, mitigating any adverse effects on bone mineralization.

STAR★METHODS

Detailed methods are provided in the online version of this paper and include the following:

- KEY RESOURCE TABLE
- CONTACT FOR REAGENT AND RESOURCE SHARING
- EXPERIMENTAL MODEL AND SUBJECT DETAILS
  - Human vessels samples
  - Fetal sheep bone and osteoblasts
  - *In vitro* MC3T3 osteoblast cell line model
  - *In vitro* human vascular smooth muscle cell (hVSMC) model of calcification
  - *In vitro* bovine VSMC (bVSMC) *in vitro* model of calcification
  - Warfarin-induced vascular calcification in rats
  - Rat CKD Model
  - PARP inhibitor treatment
- METHOD DETAILS
  - *Ex vivo* sample imaging (Figures 1 and S1)
  - *Ex vivo* vascular sample mineral, PAR and γH2A.X imaging
  - *In vitro* cell model imaging and NMR spectroscopy (Figures 2 and S2)
  - Extracellular matrix synthesis for PAR binding assessment (Figures 3 and S3)
  - *In vitro*, cell-free nidus calcification models (Figures 4, 5, S4, and S5)
  - Effect of PARP inhibitors on *in vitro* human VSMC calcification (Figures 6 and S6)
  - Rat CKD model
- QUANTIFICATION AND STATISTICAL ANALYSIS
  - Rat CKD model (Figures 7 and S7)

SUPPLEMENTAL INFORMATION

Supplemental Information can be found online at https://doi.org/10.1016/j.celrep.2019.05.038.

ACKNOWLEDGMENTS

This study was funded by the British Heart Foundation (project grant PG/15/38/31466 to C.M.S. [principal investigator], M.J.D., and K.H.M.; and programme grant RG/17/2/32808 to C.M.S.). The NMR data in Figure S3 was recorded through funding from the EU iNEXT (Infrastructure for NMR, EM, and X-rays for Translational Research, GA 653706) scheme (PID 1489), from which W.Y.C. also received support. R.L. acknowledges funding from the Cambridge Trust and China Scholarship Council. A.G. was supported by the EPSRC (UK) Cambridge NanoDTC (EP/G037221/1). I.G. was funded by an EPSRC doctoral training studentship. A.M.P. was funded by the Raymond and Beverly Sackler Fund for Physics of Medicine, University of Cambridge. O.A.S. acknowledges support from EPSRC (UK) for Programme Grant (NOtCH, EP/L027151/1). The *in vivo* rat model work (Figures 7 and S7) and analysis of PARP inhibitors (Figure S6; Table S2) was funded by Cycle Pharmaceuticals Ltd. (for A.V., P.C.D., E.N., and U.B.).

AUTHOR CONTRIBUTIONS

K.H.M. recorded all the electron microscopy data and confocal imaging for the *in vivo* models (Figures 1, 7G, and S1), electron microscopy on all *in vitro* models (Figures 4, 5, S2D, S2I, S2J, S4, and S5), and quantification in Figures 4, 5, S4, S5, and S7C and helped to write the text. S.F.L. and L.-M.N. performed the extranuclear PAR analysis in Figures 1A and S1C. S.A. did immunohistochemistry for human and rat aortic sections and quantifications in Figures 1D, 7B–7F, and S1F. R.R. and I.G., advised by R.A.B., carried out the *in vitro* cell culture work in Figures 2 and S2. R.R. and U.B. carried out the confocal and multiphoton imaging in Figures 2 and S2. A.M.P. and

M.J.D. carried out the quantification of extranuclear PAR in Figures 2 and S2. R.H. carried out the experiments in Figure 3, along with M.W., and Figure 6. A.M.C. performed the slot blot analysis and bead pull-downs for PAR in Figures 3 and 6. R.L., W.Y.C., and H.O. performed and analyzed the 2D NMR experiments in Figures S3A and S3B. D.G.R. advised on NMR work in Figure S3 and experimental design throughout. I.G. carried out CD spectroscopy in Figure 4E. A.G., K.H.M., and A.M.P., advised by O.A.S., carried out and analyzed the DLS work in Figures 4 and S4. J.N.S. carried out the TEM work in Figure 5F and advised on electron microscopy work throughout. M.S. performed the calcification experiment with PJ34 on bVSMCs in Figure 6 and R.R. on MC3T3s. U.B. carried out inhibitor testing in PARP activity assay in Figure S6 and Table S2. J.A.H. was involved in the planning of these experiments and the PARP inhibitor assays in Figure S6 and Table S2. A.V., P.C.D., and E.N. carried out the *in vivo* work in Figures 7A, S7A, and S7B. J.B. induced the rat warfarin model of calcification (Figures 7 and S7) and collected samples for EM. C.M.S. and M.J.D. designed experiments and wrote the manuscript.

DECLARATION OF INTERESTS

J.A.H. is the Chairman of Cycle Pharmaceuticals, Ltd. M.J.D.'s and C.M.S.'s research groups receive licensing fees from Cycle Pharmaceuticals Ltd. for US patent no. US10159685B2 on the use of PARP inhibitors in vascular calcification.

Received: May 23, 2018
Revised: April 3, 2019
Accepted: May 9, 2019
Published: June 11, 2019

REFERENCES

Abedin, M., Tintut, Y., and Demer, L.L. (2004). Vascular calcification: mechanisms and clinical ramifications. Arterioscler. Thromb. Vasc. Biol. *24*, 1161–1170.

Addison, W.N., Nelea, V., Chicatun, F., Chien, Y.C., Tran-Khanh, N., Buschmann, M.D., Nazhat, S.N., Kaartinen, M.T., Vali, H., Tecklenburg, M.M., et al. (2015). Extracellular matrix mineralization in murine MC3T3-E1 osteoblast cultures: an ultrastructural, compositional and comparative analysis with mouse bone. Bone *71*, 244–256.

Al-Qtaitat, A.I. (2014). A Review of Non-Collagenous Proteins; their Role in Bone. Am. J. Life Sci. *2*, 351–355.

Alano, C.C., Kauppinen, T.M., Valls, A.V., and Swanson, R.A. (2006). Minocycline inhibits poly(ADP-ribose) polymerase-1 at nanomolar concentrations. Proc. Natl. Acad. Sci. USA *103*, 9685–9690.

Altmeyer, M., Neelsen, K.J., Teloni, F., Pozdnyakova, I., Pellegrino, S., Grøfte, M., Rask, M.D., Streicher, W., Jungmichel, S., Nielsen, M.L., and Lukas, J. (2015). Liquid demixing of intrinsically disordered proteins is seeded by poly(ADP-ribose). Nat. Commun. *6*, 8088.

Ba, X., and Garg, N.J. (2011). Signaling mechanism of poly(ADP-ribose) polymerase-1 (PARP-1) in inflammatory diseases. Am. J. Pathol. *178*, 946–955.

Bai, P., Nagy, L., Fodor, T., Liaudet, L., and Pacher, P. (2015). Poly(ADP-ribose) polymerases as modulators of mitochondrial activity. Trends Endocrinol. Metab. *26*, 75–83.

Becker, A., Epple, M., Müller, K.M., and Schmitz, I. (2004). A comparative study of clinically well-characterized human atherosclerotic plaques with histological, chemical, and ultrastructural methods. J. Inorg. Biochem. *98*, 2032–2038.

Bertazzo, S., Gentleman, E., Cloyd, K.L., Chester, A.H., Yacoub, M.H., and Stevens, M.M. (2013). Nano-analytical electron microscopy reveals fundamental insights into human cardiovascular tissue calcification. Nat. Mater. *12*, 576–583.

Bini, A., Mann, K.G., Kudryk, B.J., and Schoen, F.J. (1999). Noncollagenous bone matrix proteins, calcification, and thrombosis in carotid artery atherosclerosis. Arterioscler. Thromb. Vasc. Biol. *19*, 1852–1861.

Boskey, A.L. (1989). Noncollagenous matrix proteins and their role in mineralization. Bone Miner. *6*, 111–123.

Boskey, A.L. (2007). Mineralization of Bones and Teeth. Elements *3*, 387–393.

Boskey, A.L., and Villarreal-Ramirez, E. (2016). Intrinsically disordered proteins and biomineralization. Matrix Biol. *52-54*, 43–59.

Boström, K., Watson, K.E., Horn, S., Wortham, C., Herman, I.M., and Demer, L.L. (1993). Bone morphogenetic protein expression in human atherosclerotic lesions. J. Clin. Invest. *91*, 1800–1809.

Brunyanszki, A., Szczesny, B., Virág, L., and Szabo, C. (2016). Mitochondrial poly(ADP-ribose) polymerase: The Wizard of Oz at work. Free Radic. Biol. Med. *100*, 257–270.

Chen, J.R., Lazarenko, O.P., Blackburn, M.L., Mercer, K.E., Badger, T.M., and Ronis, M.J.J. (2015). P47^{phox}-Nox2-dependent ROS signaling inhibits early bone development in mice but protects against skeletal aging. J. Biol. Chem. *290*, 14692–14704.

Chow, W.Y., Rajan, R., Muller, K.H., Reid, D.G., Skepper, J.N., Wong, W.C., Brooks, R.A., Green, M., Bihan, D., Farndale, R.W., et al. (2014). NMR spectroscopy of native and in vitro tissues implicates polyADP ribose in biomineralization. Science *344*, 742–746.

Combes, C., Cazalbou, S., and Rey, C. (2016). Apatite Biominerals. Minerals (Basel) *6*, 34.

Demer, L.L., and Tintut, Y. (2008). Vascular calcification: pathobiology of a multifaceted disease. Circulation *117*, 2938–2948.

Diwan, V., Brown, L., and Gobe, G.C. (2018). Adenine-induced chronic kidney disease in rats. Nephrology (Carlton) *23*, 5–11.

Donley, G.E., and Fitzpatrick, L.A. (1998). Noncollagenous matrix proteins controlling mineralization; possible role in pathologic calcification of vascular tissue. Trends Cardiovasc. Med. *8*, 199–206.

Eustermann, S., Brockmann, C., Mehrotra, P.V., Yang, J.-C., Loakes, D., West, S.C., Ahel, I., and Neuhaus, D. (2010). Solution structures of the two PBZ domains from human APLF and their interaction with poly(ADP-ribose). Nat. Struct. Mol. Biol. *17*, 241–243.

Glimcher, M.J. (1984). Recent studies of the mineral phase in bone and its possible linkage to the organic matrix by protein-bound phosphate bonds. Philos. Trans. R. Soc. Lond. B Biol. Sci. *304*, 479–508.

Gourgas, O., Marulanda, J., Zhang, P., Murshed, M., and Cerruti, M. (2018). Multidisciplinary Approach to Understand Medial Arterial Calcification. Arterioscler. Thromb. Vasc. Biol. *38*, 363–372.

Hutcheson, J.D., Goettsch, C., Bertazzo, S., Maldonado, N., Ruiz, J.L., Goh, W., Yabusaki, K., Faits, T., Bouten, C., Franck, G., et al. (2016). Genesis and growth of extracellular-vesicle-derived microcalcification in atherosclerotic plaques. Nat. Mater. *15*, 335–343.

Iyemere, V.P., Proudfoot, D., Weissberg, P.L., and Shanahan, C.M. (2006). Vascular smooth muscle cell phenotypic plasticity and the regulation of vascular calcification. J. Intern. Med. *260*, 192–210.

Jahnen-Dechent, W., Schäfer, C., Ketteler, M., and McKee, M.D. (2008). Mineral chaperones: a role for fetuin-A and osteopontin in the inhibition and regression of pathologic calcification. J. Mol. Med. (Berl.) *86*, 379–389.

Johnson, R.C., Leopold, J.A., and Loscalzo, J. (2006). Vascular calcification: pathobiological mechanisms and clinical implications. Circ. Res *99*, 1044–1059.

Jungmichel, S., Rosenthal, F., Altmeyer, M., Lukas, J., Hottiger, M.O., and Nielsen, M.L. (2013). Proteome-wide identification of poly(ADP-Ribosyl)ation targets in different genotoxic stress responses. Mol. Cell *52*, 272–285.

Kapustin, A., and Shanahan, C.M. (2009). Targeting vascular calcification: softening-up a hard target. Curr. Opin. Pharmacol. *9*, 84–89.

Kapustin, A.N., Davies, J.D., Reynolds, J.L., McNair, R., Jones, G.T., Sidibe, A., Schurgers, L.J., Skepper, J.N., Proudfoot, D., Mayr, M., and Shanahan, C.M. (2011). Calcium regulates key components of vascular smooth muscle cell-derived matrix vesicles to enhance mineralization. Circ. Res. *109*, e1–e12.

Kapustin, A.N., Chatrou, M.L.L., Drozdov, I., Zheng, Y., Davidson, S.M., Soong, D., Furmanik, M., Sanchis, P., De Rosales, R.T.M., Alvarez-Hernandez,

D., et al. (2015). Vascular smooth muscle cell calcification is mediated by regulated exosome secretion. Circ. Res. *116*, 1312–1323.

Kim, N.H., Kim, H.Y., An, H., Seo, J.A., Kim, N.H., Choi, K.M., Baik, S.H., Choi, D.S., and Kim, S.G. (2013). Effect of cilostazol on arterial stiffness and vascular adhesion molecules in type 2 diabetic patients with metabolic syndrome: a randomised, double-blind, placebo-controlled, crossover trial. Diabetol. Metab. Syndr. *5*, 41.

Landis, W.J., Song, M.J., Leith, A., McEwen, L., and McEwen, B.F. (1993). Mineral and organic matrix interaction in normally calcifying tendon visualized in three dimensions by high-voltage electron microscopic tomography and graphic image reconstruction. J. Struct. Biol. *110*, 39–54.

Lee, J.H., Park, S.Y., Shin, H.K., Kim, C.D., Lee, W.S., and Hong, K.W. (2007). Poly(ADP-ribose) polymerase inhibition by cilostazol is implicated in the neuroprotective effect against focal cerebral ischemic infarct in rat. Brain Res. *1152*, 182–190.

Li, M., Zhao, L., Liu, J., Liu, A.L., Zeng, W.S., Luo, S.Q., and Bai, X.C. (2009). Hydrogen peroxide induces G2 cell cycle arrest and inhibits cell proliferation in osteoblasts. Anat. Rec. (Hoboken) *292*, 1107–1113.

Liu, Y., Drozdov, I., Shroff, R., Beltran, L.E., and Shanahan, C.M. (2013). Prelamin A accelerates vascular calcification via activation of the DNA damage response and senescence-associated secretory phenotype in vascular smooth muscle cells. Circ. Res. *112*, e99–e109.

Luo, X., and Kraus, W.L. (2012). On PAR with PARP: cellular stress signaling through poly(ADP-ribose) and PARP-1. Genes Dev. *26*, 417–432.

Mahamid, J., Aichmayer, B., Shimoni, E., Ziblat, R., Li, C., Siegel, S., Paris, O., Fratzl, P., Weiner, S., and Addadi, L. (2010). Mapping amorphous calcium phosphate transformation into crystalline mineral from the cell to the bone in zebrafish fin rays. Proc. Natl. Acad. Sci. USA *107*, 6316–6321.

McNally, E.A., Schwarcz, H.P., Botton, G.A., and Arsenault, A.L. (2012). A model for the ultrastructure of bone based on electron microscopy of ion-milled sections. PLoS ONE *7*, e29258.

Mody, N., Parhami, F., Sarafian, T.A., and Demer, L.L. (2001). Oxidative stress modulates osteoblastic differentiation of vascular and bone cells. Free Radic. Biol. Med. *31*, 509–519.

Moe, S.M., O'Neill, K.D., Duan, D., Ahmed, S., Chen, N.X., Leapman, S.B., Fineberg, N., and Kopecky, K. (2002). Medial artery calcification in ESRD patients is associated with deposition of bone matrix proteins. Kidney Int. *61*, 638–647.

Nudelman, F., Pieterse, K., George, A., Bomans, P.H.H., Friedrich, H., Brylka, L.J., Hilbers, P.A.J., de With, G., and Sommerdijk, N.A. (2010). The role of collagen in bone apatite formation in the presence of hydroxyapatite nucleation inhibitors. Nat. Mater. *9*, 1004–1009.

Nudelman, F., Bomans, P.H.H., George, A., de With, G., and Sommerdijk, N.A.J.M. (2012). The role of the amorphous phase on the biomimetic mineralization of collagen. Faraday Discuss. *159*, 357–370.

Olszta, M., Cheng, X., Jee, S., Kumar, R., Kim, Y., Kaufman, M., Douglas, E., and Gower, L. (2007). Bone structure and formation: A new perspective. Mater. Sci. Eng. Rep. *58*, 77–116.

Peiró, C., Lafuente, N., Matesanz, N., Cercas, E., Llergo, J.L., Vallejo, S., Rodríguez-Mañas, L., and Sánchez-Ferrer, C.F. (2001). High glucose induces cell death of cultured human aortic smooth muscle cells through the formation of hydrogen peroxide. Br. J. Pharmacol. *133*, 967–974.

Price, P.A., Faus, S.A., and Williamson, M.K. (1998). Warfarin causes rapid calcification of the elastic lamellae in rat arteries and heart valves. Arterioscler. Thromb. Vasc. Biol. *18*, 1400–1407.

Proudfoot, D., and Shanahan, C.M. (2001). Biology of Calcification in Vascular Cells: Intima versus Media. Herz *4*, 245–251.

Proudfoot, D., Davies, J.D., Skepper, J.N., Weissberg, P.L., and Shanahan, C.M. (2002). Acetylated low-density lipoprotein stimulates human vascular smooth muscle cell calcification by promoting osteoblastic differentiation and inhibiting phagocytosis. Circulation *106*, 3044–3050.

Rey, C., Combes, C., Drouet, C., and Glimcher, M.J. (2009). Bone mineral: update on chemical composition and structure. Osteoporosis Int *20*, 1013–1021.

Reynolds, J.L., Joannides, A.J., Skepper, J.N., McNair, R., Schurgers, L.J., Proudfoot, D., Jahnen-Dechent, W., Weissberg, P.L., and Shanahan, C.M. (2004). Human vascular smooth muscle cells undergo vesicle-mediated calcification in response to changes in extracellular calcium and phosphate concentrations: a potential mechanism for accelerated vascular calcification in ESRD. J. Am. Soc. Nephrol. *15*, 2857–2867.

Reznikov, N., Bilton, M., Lari, L., Stevens, M.M., and Kröger, R. (2018). Fractal-like hierarchical organization of bone begins at the nanoscale. Science *360*, eaao2189.

Robaszkiewicz, A., Erdélyi, K., Kovács, K., Kovács, I., Bai, P., Rajnavölgyi, E., and Virág, L. (2012). Hydrogen peroxide-induced poly(ADP-ribosyl)ation regulates osteogenic differentiation-associated cell death. Free Radic. Biol. Med. *53*, 1552–1564.

Schreiber, V., Dantzer, F., Ame, J.-C., and de Murcia, G. (2006). Poly(ADP-ribose): novel functions for an old molecule. Nat. Rev. Mol. Cell Biol. *7*, 517–528.

Severson, A.R., Ingram, R.T., and Fitzpatrick, L.A. (1995). Matrix proteins associated with bone calcification are present in human vascular smooth muscle cells grown in vitro. In Vitro Cell. Dev. Biol. Anim. *31*, 853–857.

Shanahan, C.M., Crouthamel, M.H., Kapustin, A., and Giachelli, C.M. (2011). Arterial calcification in chronic kidney disease: key roles for calcium and phosphate. Circ. Res. *109*, 697–711.

Shao, J.-S., Cai, J., and Towler, D.A. (2006). Molecular mechanisms of vascular calcification: lessons learned from the aorta. Arterioscler. Thromb. Vasc. Biol. *26*, 1423–1430.

Shimamura, T. (1970). Ultrastructural studies of the medial aortic sclerosis in rats with experimentally induced chronic renal disease. Exp. Mol. Pathol. *13*, 79–88.

Vattikuti, R., and Towler, D.A. (2004). Osteogenic regulation of vascular calcification: an early perspective. Am. J. Physiol. Endocrinol. Metab. *286*, E686–E696.

Veis, A., and Dorvee, J.R. (2013). Biomineralization mechanisms: a new paradigm for crystal nucleation in organic matrices. Calcif. Tissue Int. *93*, 307–315.

Wada, T., McKee, M.D., Steitz, S., and Giachelli, C.M. (1999). Calcification of vascular smooth muscle cell cultures: inhibition by osteopontin. Circ. Res. *84*, 166–178.

Wang, X., and Li, B. (2007). Genetic studies of bone diseases: evidence for involvement of DNA damage response proteins in bone remodeling. Int. J. Biomed. Sci. *3*, 217–228.

Wang, Y., Azaïs, T., Robin, M., Vallée, A., Catania, C., Legriel, P., Pehau-Arnaudet, G., Babonneau, F., Giraud-Guille, M.M., and Nassif, N. (2012). The predominant role of collagen in the nucleation, growth, structure and orientation of bone apatite. Nat. Mater. *11*, 724–733.

Wang, Y., Von Euw, S., Laurent, G., Crevant, C., Bonhomme-Coury, L., Giraud-Guille, M.-M., Babonneau, F., Nassif, N., and Azaïs, T. (2014). Impact of collagen confinement vs. ionic substitutions on the local disorder in bone and biomimetic apatites. Mater. Horiz. *1*, 224–231.

Weiner, S., and Traub, W. (1986). Organization of hydroxyapatite crystals within collagen fibrils. FEBS Lett. *206*, 262–266.

You, A.Y.F., Bergholt, M.S., St-Pierre, J.P., Kit-Anan, W., Pence, I.J., Chester, A.H., Yacoub, M.H., Bertazzo, S., and Stevens, M.M. (2017). Raman spectroscopy imaging reveals interplay between atherosclerosis and medial calcification in the human aorta. Sci. Adv. *3*, e1701156.

STAR★METHODS

KEY RESOURCE TABLE

REAGENT or RESOURCE	SOURCE	IDENTIFIER
Antibodies		
Mouse monoclonal anti-PAR (clone 10H)	Enzo Life Sciences	Cat#ALX-804-220; RRID:AB_2272987
Mouse monoclonal anti-PAR (Clone 10H)	Lifespan Biosciences	Cat#LS-C146823; RRID:AB_11142728
Mouse monoclonal anti-PAR (clone 10H)	Abcam	Cat#ab14459; RRID:AB_301239
Mouse IgG isotype control	Abcam	Cat#ab18443; RRID:AB_2736846
Rabbit polyclonal anti-Annexin VI	Abcam	Cat#ab19416, RRID:AB_444903
Rabbit polyclonal anti-fibronectin	Abcam	Cat#ab2413; RRID:AB_2262874
Rabbit monoclonal anti-γH2AX (Clone 20E3)	Cell Signaling Technology	Cat#9718; RRID:AB_2118009
Rabbit polyclonal anti- γH2AX(phosphor S139)	Abcam	Cat#ab11174; RRID:AB_297813
Rabbit polyclonal anti-CD63 (clone H-193)	Santa Cruz Biotechnology	Cat#sc-15363; RRID:AB_648179
Rabbit polyclonal anti-αSMA	Abcam	Cat#ab5694; RRID:AB_2223021
Mouse anti-CD63	BD PharMingen	Cat# 556019; RRID:AB_396297
Goat anti-Mouse AlexaFluor 546	Invitrogen	Cat#A11003; RRID:AB_141370
Goat anti-Rabbit AlexaFluor 488	Invitrogen	Cat#A11010; RRID:AB_143156
Goat anti-mouse IgG-Alexa594	Abcam	Cat#ab150120; RRID:AB_2631447
Goat anti-mouse IgG-Alexa488	Abcam	Cat#ab150117; RRID:AB_2688012
Donkey anti-rabbit IgG-Alexa 647	Abcam	Cat#ab150075; RRID:AB_2752244
Goat anti-mouse IgG-Alexa488	Molecular Probes/Invitrogen	Cat#A11001; RRID:AB_2534069
Goat anti-rabbit IgG-Alexa568	Molecular Probes/Invitrogen	Cat#A11011; RRID:AB_143157
Goat anti-mouse IgG-15 nm gold	BBI solutions	Cat#EM.GMHL15; RRID:AB_2715551
IRDye 800CW Donkey anti-Mouse	LI-COR	Cat#926-32212 RRID:AB_621847
IRDye 800CW Donkey anti-Rabbit	LI-COR	Cat#926-68073 RRID:AB_10954442
IRDye 680RD Donkey anti-Mouse	LI-COR	Cat#926-68072 RRID:AB_10953628
IRDye 680RD Donkey anti-Rabbit	LI-COR	Cat#926-68073 RRID:AB_10954442
Biological Samples		
Foetal sheep growth plate	Provided by Roger Brookes	N/A
Chemicals, Peptides, and Recombinant Proteins		
(+)-Sodium L-ascorbate	Sigma Aldrich	Cat#A4034
Gelatin	Sigma Aldrich	Cat#G9391
Ethanolamine	Sigma Aldrich	Cat#E0135
Glutaraldehyde	Sigma Aldrich	Cat#G6257
m-aminophenylboronic acid agarose	Sigma Aldrich	Cat#A8312
Ponceau S solution	Sigma Aldrich	Cat#P7170
Anti-pan-ADP-ribose binding reagent	Merck Millipore	MABE1016
Hydrogen Peroxide solution	Sigma Aldrich	Cat#H1009
Normal Goat Serum	Vector Laboratories	Cat#S-1000
Normal Horse Serum	Vector Laboratories	Cat#S-2000
Antigen Unmasking Solution; citric acid based	Vector Laboratories	Cat#H-3300
Active PARP1	Abcam	Cat#ab79663
Active PARP2	Abcam	Cat#ab198766
EDTA-free PAR	BioTechne/Trevigen	Custom-made
BlockAid Blocking solution	ThermoFisher	Cat#B10710
DEA (6,9-diamino-2-ethoxyacridine-DL-lactate)	Sigma	CAS 6402-23-9
Niraparib	LGM Pharma	CAS 1038915-60-4

(*Continued on next page*)

Continued

REAGENT or RESOURCE	SOURCE	IDENTIFIER
Rucaparib	LGM Pharma	CAS 28173-50-2
Veliparib	LGM Pharma	CAS 912444-00-9
Olaparib	LGM Pharma	CAS 763113-22-0
PJ34	AdipoGen	CAS 344458-15-7
Minocycline	Cycle Pharmaceuticals	CAS 10118-90-8
Calcium Chloride Dihydrate	Sigma	C27902
Sodium Phosphate monobasic	Sigma	S-5011
O-cresolphthalein	Sigma	P5631
Ammonium Hydroxide Solution	Sigma	338818
Critical Commercial Assays		
OsteoImage Mineralization Assay	Lonza	Cat#PA-1503
Vectastain Elite ABC HRP kit (peroxidase, Mouse IgG)	Vector Laboratories	Cat#PK-6102
Vectastain Elite ABC HRP kit (peroxidase, Rabbit IgG)	Vector Laboratories	Cat#PK-6101
DAB peroxidase substrate kit	Vector Laboratories	Cat#SK-4100
PARP1 enzyme activity assay	Merck Millipore	Cat#17-10149
RNeasy mini kit	QIAGEN LTD	Cat#74104
RT2 First Strand Kit	QIAGEN LTD	Cat#330404
QuantiFast SYBR Green PCR Kit	QIAGEN LTD	Cat#204054
HT colorimetric PARP/apoptosis assay kit	Trevigen	Cat#4684-096-K
DC Protein Assay Kit	Bio-Rad Laboratories	Cat#5000116
Experimental Models: Cell Lines		
MC3T3-E1 murine calvarial osteoblasts (subclone 14)	ATCC	Cat# CRL-2594
Bovine VSMC	obtained from aortic segments from an abattoir	N/A
Primary human VSMC isolates	Explant cultures from aorta.	N/A
Oligonucleotides		
Osterix (Osx): QT00293181 1 Mm_Sp7_1_SG QuantiTect Primer Assay	QIAGEN LTD	Cat#QT00293181
Type 1 collagen alpha chain: QT00162204 1 Mm_Col1a1_1_SG QuantiTect Primer Assay	QIAGEN LTD	Cat#QT00162204
Osteopontin: QT00157724 1 Mm_Spp1_1_SG QuantiTect Primer Assay	QIAGEN LTD	Cat#QT00157724
Osteocalcin: QT00259406 1 Mm_Bglap_1_SG QuantiTect Primer Assay	QIAGEN LTD	Cat#QT00259406
Osteonectin: QT00161721 1 Mm_Sparc_1_SG QuantiTect Primer Assay	QIAGEN LTD	Cat#QT00161721
PARP2: QT00162281 1 Mm_Parp2_1_SG QuantiTect Primer Assay	QIAGEN LTD	Cat#QT00162281
PARP1: QT00157584 1 Mm_Parp1_1_SG QuantiTect Primer Assay	QIAGEN LTD	Cat#QT00157584
GAPDH: QT01658692 1 Mm_Gapdh_3_SG QuantiTect Primer Assay	QIAGEN LTD	Cat#QT01658692
Alkaline phosphatase: QT00157717 1 Mm_Alpl_1_SG QuantiTect Primer Assay	QIAGEN LTD	Cat#QT00157717
RT^2 qPCR Primer Assay for Human PARP2	QIAGEN LTD	Cat#PPH02684F
RT^2 qPCR Primer Assay for Human PARP1	QIAGEN LTD	Cat#PPH00686B
Hs_BMP2_1_SG QuantiTect Primer Assay	QIAGEN LTD	Cat#QT00012544
OCN F:GGCAGCGAGGTAGTGAAGAG	Integrated DNA Technologies	N/A
OCN R: CGATAGGCCTCCTGAAAGC	Integrated DNA Technologies	N/A
MSX2: F: AAATTCAGAAGATGGAGCGGCGTG	Integrated DNA Technologies	N/A

(*Continued on next page*)

Continued		
REAGENT or RESOURCE	SOURCE	IDENTIFIER
MSX2: R: CGGCTTCCGATTGGTCTTGTGTTT	Integrated DNA Technologies	N/A
SMA: F:TTGAAGGCAAAGACATGGCAGCAG	Integrated DNA Technologies	N/A
SMA: R:TCCACGGTAGTGCCCATCATTCTT	Integrated DNA Technologies	N/A
SM22: F:TTGAAGGCAAAGACATGGCAGCAG	Integrated DNA Technologies	N/A
SM22: R:TCCACGGTAGTGCCCATCATTCTT	Integrated DNA Technologies	N/A
Software and Algorithms		
ImageJ	NIH	https://imagej.nih.gov/ij/download.html
GraphPad Prism	GraphPad Software	https://www.graphpad.com
Leica Application Suite	Leica	http://www.leica-microsystems.com/products/microscope-software/
Bio-Rad CFX Maestro (Real-time PCR analysis)	Bio-Rad	N/A
Other		
Bio-Dot SF Microfiltration Apparatus	Bio-Rad	#1706542
Casein diet / 3.0 mg/g of warfarin and 1.5 mg/g of vitamin K_1	AB diets	Code 4165.00

CONTACT FOR REAGENT AND RESOURCE SHARING

Further information and requests for resources and reagents should be directed to and will be fulfilled by the Lead Contact, Melinda Duer (mjd13@cam.ac.uk).

EXPERIMENTAL MODEL AND SUBJECT DETAILS

Human vessels samples

Normal vessels and atherosclerotic plaques (aorta and carotid) were obtained from transplant donors and patients undergoing carotid endarterectomy respectively, with informed consent and approval from the Cambridge Local Research Ethics Committee LREC 97/084. Age and gender of patients are reported in Table S1. All human materials were handled in compliance with the Human Tissue Act (2004, UK).

Fetal sheep bone and osteoblasts

Growth plate

Foetal sheep bones (humerus and femur) were dissected out immediately after the sacrifice of the sheep one week before the fetus would be full-term. The bones were put in a plastic bag and transported to the lab on wet ice. The head of each bone was cut longitudinally using a microtome knife. The bones were cleaved again horizontally into two smaller pieces. The smaller pieces were placed in white plastic embedding pots on TissueTek OCT compound and were then covered with OCT The plastic containers were placed on dry ice for about 15-20 min for the OCT to solidify. Then the samples were wrapped up tightly in tin foil and cooled to -20°C. Cryosections were cut between 8 and 18 μm thick.

Isolation of osteoblasts

Fetal sheep osteoblasts were isolated from a fetus removed from an 18 weeks pregnant sheep sacrificed for an unrelated study. Femurs were removed from the fetus. After washing several times with 1% trigene (Medichem International), the femur was stripped of muscle and non-osseous tissue to expose the bone which was sectioned into small longitudinal pieces and washed with 70% ethanol followed by repeated washings with Minimum Essential Medium (MEM; Invitrogen) to remove all traces of ethanol. Bone strips were then transferred to Dulbecco's Modified Eagle Medium (DMEM; Invitrogen) containing bacterial collagenase A (0.5 mg/mL) and dispase II (3 mg/mL) both from Roche Diagnostics. A total of 100 mL of enzyme-media mixture was used for bone sections taken from 3 limbs. Bone strips were incubated at 37°C in a shaking water bath for 3 hours to release osteoblasts into the medium. After incubation the cell suspension was transferred to a fresh tube and the bone sections were rinsed in DMEM with 20% fetal calf serum (FCS; Invitrogen) to stop the enzymatic digestion. Rinse medium and cell suspension were pooled and passed through a 40 μm mesh filter (Appleton Woods). The cell suspension was then centrifuged at 1000 g for 5 min at room temperature to pellet the cells. The pellet was re-suspended in DMEM complete medium and transferred to two T-175 cm^3 culture flasks (Nunc) and placed in a 37°C CO_2 incubator. When the cultures were almost confluent, cells were detached with 10 mL of 0.25% trypsin containing 1 mM EDTA (SigmaAldrich) and incubatied for 5 min at room temperature. The flasks were tapped at the end of incubation period to completely dislodge the cells from the flask. Trypsin was neutralized by adding 15 mL of DMEM complete

media to the culture flask. The cell suspension was centrifuged in a 50 mL tube (Greiner) at 1200 rpm for 5 min and resuspended in 10 mL of DMEM. The cells were transferred into T-175 cm^3 culture flasks and were expanded to passage 3 for subsequent experiments.

Basal Medium Eagle (BME) complete medium was prepared by adding 10% FCS, 30 μg/mL L-ascorbic acid 2-phosphate (Sigma), 10mL/L L-glutamine-penicillin- streptomycin (200 mM L-glutamine, 10,000 units/ml penicillin, and 10 mg/ml streptomycin in 0.9% sodium chloride; Sigma). DMEM complete medium was prepared by adding 10% FCS, 30 μg/mL L-ascorbic acid 2-phosphate, and 10mL/L L-glutamine-penicillin-streptomycin. All supplements were filter sterilized (0.22 μm filter, Appleton Woods) before addition.

In vitro MC3T3 osteoblast cell line model

MC3T3-E1 murine calvarial osteoblasts (subclone 14) were purchased from ATCC (gender unknown). MC3T3- E1 cells were cultured in α-MEM (Life Technologies) growth medium supplemented with 10% FBS (Life Technologies), L-Glutamine–Penicillin–Streptomycin solution (200 mM L-glutamine, 10,000 U penicillin and 10mg steptomycin/ml, Sigma), at humidified 37°C, 5% CO_2 incubator. Cell differentiation and matrix mineralization were initiated when the cell density reached approximately 80% confluence with mineralization medium. Mineralization medium is growth medium supplemented with 50 μg/ml ascorbic acid, 4 mM β-glycerophosphate (Sigma) and 10nM dexamethasone (Sigma). The medium was changed every three days.

For calcium estimation assays and qRT-PCR experiments, MC3T3 E1 cells were seeded in 24-well plates for each time points. 1×10^4 cells were plated per well and were grouped into three sets each for control, mineralized and mineralized with PJ34. The control group were cultured in α-MEM growth media, mineralized group in mineralizing media and mineralized with PARP inhibitor group in mineralizing media with PARP inhibitor PJ34 (5 μM in DMSO). The control group for the PARP inhibitor studies additionally had 5 μL DMSO added per well.

In vitro human vascular smooth muscle cell (hVSMC) model of calcification

Primary human VSMCs were obtained from a medial aortic explant from a healthy 35 year old female transplant donor with approval from the Cambridge Local Research Ethics Committee LREC 97/084. The cells were cultured in M199 media (Sigma-Aldrich) supplemented with 20% fetal bovine serum (FBS) and 100 U/ml penicillin, 100U/ml streptomycin and 0.29 mg/ml glutamine (1% PSG), and incubated at 37°C with 5% CO_2.

To induce mineralization, human VSMCs were seeded and then cultured for 1 day in M199 media supplemented with 5% FBS and 1% PSG. Then the cells were incubated in high Ca/P media (M199 media supplemented with 5% FBS and 1% PSG, with a final concentration of 2.7 mM Ca^{2+} and 2.5 mM phosphate) or with control media M199 with no additional Ca^{2+} or P (M199 media supplemented with 5% FBS and 1% PSG, with a final concentration of 1.8 mM Ca^{2+}/1.0 mM P). The media was refreshed every 2-3 days.

In vitro bovine VSMC (bVSMC) *in vitro* model of calcification

bVSMCs were obtained from aortic segments from an abattoir. bVSMC cells were seeded on sterile 8-well chamber slide at a cell density of $1x10^4$ cells per well in DMEM (Life Technologies) growth medium supplemented with 10% FBS (Life Technologies) and L-Glutamine–Penicillin–Streptomycin solution (200 mM L-glutamine, 10,000 U penicillin and 10mg steptomycin/ml, Sigma), at humidified 37°C, 5% CO_2 incubator. After three days, growth medium was replaced by mineralization medium (growth medium+4mM β-glycerophosphate, 50 μg/ml ascorbic acid + 10nM dexamethasone) and was changed every three days till day twenty when the mineralized nodules are wide spread in culture.

Warfarin-induced vascular calcification in rats

All procedures were performed in accordance with licenses and guidelines approved by the UK Home Office and were approved by a King's College ethics committee. Sprague Dawley rats (n = 6; male; 21-27 days old) were purchased from Charles River Laboratories. After an acclimatisation period of 7 days, the rats were fed with a custom made rodent diet containing 3.0 mg/g of warfarin and 1.5 mg/g of vitamin K_1 (AB diets, Netherlands) After 7 days on warfarin diet, the rats were euthanised and aortas harvested for TEM.

Rat CKD Model

Experimental procedures were conducted according to the National Institutes of Health Guide for the Care and Use of Laboratory Animals 85-23 (1996) and approved by the University of Antwerp Ethics Committee (ethical approval reference number: 2018-19).

Seven-week old male Wistar Han rats (225-250 g, Charles River, Lille, France) (RRID: RGD_2308816) were housed two per cage and maintained in a controlled environment with a 12:12 light-dark cycle, room temperature of 22°C and free access to tap water and their allotted diet. A number of 84 rats were randomly assigned to the following 5 study groups: (i) rats with normal renal function treated with vehicle (i.e., tap water) (NRF + vehicle) (n = 4); (ii) CKD rats daily treated with vehicle (i.e., tap water) for 6 weeks (CKD + vehicle 6wks) (n = 14); (iii) CKD rats daily treated with 5 mg/kg minocycline for 6 weeks (CKD + MC/05 6wks) (n = 14); (iv) CKD rats daily treated with 10 mg/kg minocycline for 6 weeks (CKD + MC/10 6wks) (n = 14); (v) CKD rats daily treated with 50 mg/kg minocycline for 6 weeks (CKD + MC/50 6wks) (n = 14).

Upon arrival in the animal facility all groups were conditioned to a high phosphorus diet (1.03% P and 1.06% Ca) (SSNIFF Specialdiäten, Soest, Germany) for 2 weeks after which CKD and vascular calcification was induced in all study groups by feeding the

animals a diet containing 0.75% adenine (Acros Organics, Geel, Belgium) (with a 0.92% P and 1.0% Ca content in combination with a low protein content (2.5% instead of 19.0%)) (SSNIFF Specialdiäten) for 4 weeks followed by the high phosphorus diet (1.03% P and 1.06% Ca) until the end of the study. Control animals with normal renal function were fed a standard rodent maintenance diet (0.7% P and 1.0% Ca) (SSNIFF Specialdiäten) for the entire study period.

PARP inhibitor treatment

Minocycline treatment was initiated one week after the start of adenine dosing at three different doses (5, 10 or 50 mg/kg/day). Minocycline was dissolved in tap water and a constant dose volume of 10 ml/kg was used. Tap water was used as vehicle in the respective groups. As the half-life of minocycline in the rat is about 3 to 3.5 hours, minocycline was dosed twice a day half of the indicated dose by gavage with a time interval of 7 hours (Monday till Friday). During the weekends, rats were gavaged the indicated dose once a day. Animals were subjected to daily treatment for 6 weeks (week 1 until 7) or 4 weeks (week 1 until 5).

At the end of the study, all animals were sacrificed by exsanguination through the retro-orbital plexus after anesthesia with 60 mg/kg ketamine (Pfizer, Puurs, Belgium) and 7.5 mg/kg xylazine (Bayer SA NV, Diegem, Belgium) via intraperitoneal injection.

At baseline (week 0), before start of treatment (week 1), after 3 (week 4), 4 (week 5) and 6 (week 7) weeks of treatment, animals were individually housed in a metabolic cage for 24 hours to collect urine samples followed by blood sampling. The urinary volume was recorded and samples were used for measurement of creatinine. Blood was drawn from the tail vein in restrained, conscious animals. Blood samples were allowed to clot on ice and centrifuged at high speed. The harvested serum was used to determine creatinine, calcium and phosphorus. To follow renal function serum and urinary creatinine were measured according to the Jaffé method. Creatinine clearance (ml/min) was calculated by the following formula: (urinary creatinine concentration (mg/dl) x urinary volume (ml)) / (serum creatinine concentration (mg/dl) x 1440 minutes). Total serum phosphorus levels were analyzed with the Ecoline S Phosphate kit (Diasys, Holzheim, Germany) and serum calcium levels were determined with flame atomic absorption spectrometry (FAAS) (Perkin-Elmer Model AAnalyst 800, Wellesley, MA, USA) after appropriate dilution in 0.1% $La(NO_3)_3$ to eliminate chemical interference.

METHOD DETAILS

Ex vivo sample imaging (Figures 1 and S1)

Ex vivo *fetal sheep growth plate mineral, PAR and γH2A.X imaging*

Frozen sections of fetal sheep growth plate were thawed for 10 min, soaked for 5 min in PBS to remove OCT and fixed in 100% cold methanol (−20°C) for 10 min. Then, the sections were permeabilized at RT for 15 min with 0.2% TX100/PBS, then blocked for 30 min with 3% BSA/0.2% TX100/PBS.

Then, sections were incubated with the primary antibodies (mouse-anti-PAR clone 10H, 1:300 in blocking solution, 600 μl/slide, LSBio, rabbit-anti-H2A.X, 1:400 in blocking buffer (600 μl/slide), New England Biolabs) at RT for 1h. After rinsing 2x, then washing 3-4x for 5 minutes each in 0.2% Tween 20/TBS, sections were incubated with the secondary antibodies for 45 min at RT (goat-anti-mouse IgG-Alexa488, 1:300 in blocking solution, 500 μl/slide, Molecular Probes; goat-anti—rabbitIgG (H+L)-Alexa568, 1:300 in blocking buffer, Invitrogen). After washing the slides 3x for 5 min each in 0.2% Tween 20/TBS and twice in PBS, cell nuclei were stained using Hoechst dye (10 min at RT, 5 μg/ml Hoechst in PBS). After rinsing twice in PBS, slides were covered with coverslips using ProLong Antifade (Molecular Probes). Sections were viewed in a Leica SP2 confocal laser fluorescence microscope using the 405 nm, 488 nm and 561 nm laser lines to excite Hoechst, Alexa488 (PAR) and Alexa568 (γH2A.X) dyes, respectively. Mineral was visible in phase-contrast mode as dark deposits/structures.

Ex vivo vascular sample mineral, PAR and γH2A.X imaging

Low resolution imaging. Human aortic and carotid samples were dissected and fixed into 10% neutral buffered formalin. These samples were then embedded in paraffin wax blocks and cut in 7 μm thick sections. Immunohistochemistry staining for PAR expression was performed using human aortic (n = 13) and carotid (n = 10) samples from normal and diseased patients. Parallel sections of the same samples were processed for von Kossa staining to visualize calcification and counterstained with 0.1% of nuclear fast red solution. The % of calcified area was measured by the threshold method using ImageJ software. For the co-localization of PAR, mineral and elastin in medial calcification by fluorescence microscopy the same histological sections were used. Elastin was visualized by its autofluorescence (405 nm laser line), mineral was imaged in reflectance mode (488 nm laser line) and PAR was visualized in transmitted mode. Samples were viewed in a Leica SP2 laser confocal microscope using using a 63x/NA 0.9 water-immersion objective. In all experiments, images were acquired in sequential mode.

High resolution imaging. Frozen sections of human carotid and coronary artery lesions were thawed, rehydrated for 5 min in 0.9% saline and then fixed for 10 min in 100% methanol cooled to −20°C. Residual methanol was removed by rinsing 2x in PBS. All following staining steps were performed at RT. In the calcein experiment, lesion mineral deposits were stained with the Ca^{2+}-binding dye calcein (4 μM in PBS, Sigma C-0875) for 1h; Hoechst 33258 (Sigma B-2883, at 10 μg/ml) was included in the staining solution to

counter-stain nuclei. Unbound dyes were removed by washing 3x for 5 min each in PBS and sections were then mounted in ProLong Antifade Gold (Invitrogen).

For the double immunostaining of PAR and the DNA damage marker γH2A.X, sections were fixed with cold methanol (as above) and subsequently permeabilized with 0.2% Triton X-100/ PBS for 15 min. After blocking non-specific binding sites with blocking buffer (3% BSA/ 0.2% TX100/PBS) for 30 min, sections were incubated with primary antibodies (mouse-anti-PAR antibody, clone 10H, Abcam, 1:300 in blocking buffer and rabbit-anti-H2A.X antibody, 20E3, New England Biolabs, 1:400 in blocking buffer) for 1h. After washing sections 4x for 5 min each in washing buffer (0.2% TX100/PBS), sections were incubated with secondary antibodies (goat-anti-mouseIgG-Alexa488 and goat-anti-rabbitIgG-Alexa568, Invitrogen, both at 1:300 in blocking buffer) for 45 min. Unbound antibodies were removed by washing 3x for 5 min each in washing buffer and 2x in PBS, then nuclei were counter-stained with 10 μg/ml Hoechst for 10 min. Sections were rinsed 2x with PBS and then mounted as above. Samples were viewed using a Leica SP2 confocal fluorescence microscope.

In the calcein experiment, lesion mineral deposition was visualized in both transmission and fluorescence mode. Calcein and Hoechst were excited using the 488 nm and 405 nm laser lines respectively and images were acquired using a 20x/NA 0.5 water-immersion objective. In the double immunostaining experiment, images were acquired using a 63x/NA 1.2 water-immersion objective. The 405 nm, 488 nm and 561 nm laser lines were used to excite Hoechst, Alexa488 (PAR) and Alexa568 (H2A.X) dyes, respectively.

In vitro cell model imaging and NMR spectroscopy (Figures 2 and S2)

All *in vitro* experiments using cultured VSMCs or MC3T3 –E1 cells were performed with 3-5 experimental replicates. At least 3 independent experiments were performed for each procedure.

Imaging of MC3T3 and bVSMC cell cultures

Confocal Imaging was performed on Leica TCS SP8 confocal microscope (Leica Microsystems). To avoid in-between channel crosstalk all images were taken in frame sequential mode. Schedule for the dyes was as follows: DAPI excitation 405/ emission 410-540 nm, calcein ex496/em500-520 nm and Alexa-594 ex594/ em600-700 nm. Multiphoton imaging was performed on LaVision BioTec TriM Scope II (LaVision BioTec GmbH, Germany) equipped with the Inside Deepsee laser light source (Spectra Physics, CA, USA). Excitation of 1140 nm allowed tri-photon excitation of DAPI (emission collected below 495 nm), generation of second harmonic (SHG) of collagen (collected 495-560 nm) and two-photon excitation of Alexa-594 (collected above 560 nm).

The fraction of PAR staining which was non-nuclear, was assessed by thresholding the images of PAR and DAPI staining in ImageJ and calculating the fraction of PAR-positive pixels that did not overlap with DAPI-positive pixels.

PAR detection in MC3T3 and bVSMC cultures

MC3T3-E1 cells were seeded on sterile 8-well chamber slide (LabTech) at a cell density of $1x10^4$ cells per well in α-MEM (Life Technologies) growth medium. After three days, growth medium was replaced by mineralization medium and was changed every three days till day thirty when the mineralized nodules are wide spread in culture. bVSMC culture mineralized at day twenty.

The mineralized cultures were washed with PBS and then Calcein AM (1 μM) in PBS was added to the cultures and incubated for 25 minutes in humidified incubator. PBS was added to negative controls instead of calcein. After incubation the cultures were washed three time with PBS. For positive controls the cultures were treated with 2mM H_2O_2 in PBS for 5 min. The cultures were washed in PBS and fixed with −20°C methanol for 10 minutes. Then cells were washed three times with PBS and permeabilized with 0.4% Tween 20 in PBS for 15 min at room temperature. The cells were washed twice in PBS and blocked with BlockAid blocking solution (Thermo) for 30 minutes at room temperature. After blocking, cells were incubated with primary antibody, mouse monoclonal anti-PAR (clone 10H) antibody (Abcam) / 300 μl/well) diluted (1:300) for overnight at 4°C. Then, cells were washed three times with 0.2% Tween 20 in PBS and fluorescent dye-labeled secondary antibody (goat-anti mouse IgG (H+L)-Alexa594 antibody (Abcam), (1:500 diluted)) in BlockAid was applied for 1 hour at room temperature. The cells were washed three times in 0.2% Tween20 in PBS then cells were rinsed twice in PBS. Afterward, PBS was removed glass coverslips were carefully mounted in Fluoroshield mounting media with DAPI (4',6-diamidino-2-phenylindole, Abcam).

Solid-state NMR of MC3T3 and bVSMC calcified matrix

A Bruker 400MHz Avance spectroscopy II spectrometer was used for solid-state ^{13}C (REDOR) and ^{31}P NMR measurements, at frequencies of 100.6MHz and 162.1MHz respectively, with standard Bruker double (for ^{31}P) and triple (for $^{13}\{^{31}P\}$ REDOR) resonance, magic-angle spinning (MAS) probes. Samples were packed into disposable high-resolution (HR)-MAS inserts, and loaded into 4 mm zirconia rotors. The rotors were rotated at magic angle at a rate of 10 kHz. Samples were characterized using direct-polarization (DP) ^{31}P NMR and $^{13}C\{^{31}P\}$ rotational-echo double resonance (REDOR) techniques (^{1}H 90° pulse length 2.5 μs, ^{31}P 90° pulse length 2.57 μs, ^{1}H-^{31}P CP contact time10ms). Recycle times of 600 s were used for ^{31}P DP experiments, and 2 s for REDOR experiments. REDOR experiments used REDOR dephasing times of 10 ms. Broadband TPPM decoupling during signal acquisition for all experiments.) ^{13}C spectra were referenced to the glycine C_α signal at 43.1 ppm relative to TMS at 0 ppm. ^{31}P spectra were referenced to the hydroxyapatite ^{31}P signal at 2.8 ppm relative to 85 wt% H_3PO_4 at 0 ppm.

Extracellular matrix synthesis for PAR binding assessment (Figures 3 and S3)

Extracellular matrix (ECM) was synthesized by growing hVSMCs to confluency on gelatin coated plates and coverslips. The cells were then extracted and the ECM used for western blotting or immunofluorescent staining.

6-well plates and coverslips were prepared by incubating with 0.2% gelatin solution in PBS at 37°C for 1 hour. The solution was removed and the gelatin was crosslinked by adding 1% glutaraldehyde in PBS and incubating at room temperature for 30 minutes. The wells and coverslips were washed with PBS and the non-crosslinked glutaraldehyde was quenched by incubating with 1 mol/l ethanolamine for 30 minutes. Cells were seeded onto the plates (50000 cells for a 6-well plate and 20000 cells for a coverslip) and after 24 hours the media was changed to complete media supplemented with 50 μg/ml l-ascorbic acid. The media was changed every 48 hours and after 7 or 18 days the cells were extracted from the ECM.

Extraction buffer (0.1% Triton X-100, 20 mmol/l NH4OH in PBS) was added to the cells and incubated for 5 minutes at 37°C. The lysed cells were removed and PBS was added to the ECM overnight at 4°C to remove remaining cell debris. The ECM was rinsed with PBS and either fixed with 3.7% PFA for immunofluorescent staining or scraped in lysis buffer (0.1% Triton X-100, 150mM NaCl in 0.1M Tris-HCl) for IP and slot blot analysis.

Immunofluorescent staining

Immunofluorescent staining of the ECM was performed to visualize the localization of PAR. Localization was studied by counterstaining with fibronectin (FN), a late endosomal marker (CD63) and calcification (Osteoimage).

Following fixing with 3.7% PFA, blocking was done for 1 hour at RT with 3% BSA (in PBS). The primary antibodies were diluted in blocking solution and incubated with the coverslips for 1 hour at RT. After washing, the secondary antibody, diluted in blocking solution, was added and incubated for 1 hour at RT in the dark. 4',6-diamidino-2-phenylindole (DAPI) was diluted in PBS and incubated with the coverslips for 5 minutes, to stain nuclei. Following washing, coverslips were mounted with Mowiol mounting medium and left to dry in the dark at RT overnight. Slides were then stored at 4°C. Images were taken using a Leica TCS SP5 confocal microscope. Quantification was performed on n > 3 images per condition.

PAR area/integrated density was analyzed using ImageJ. The images were converted to RGB stacks and measured using the auto threshold method. The default threshold method was selected, and percentage area and integrated density were measured. The percentage area is calculated by dividing the number of pixels that have been highlighted by the threshold by the total number of pixels. The integrated density is the product of the area and mean gray value (the sum of the gray values of the pixels highlighted by the threshold, divided by the total number of pixels).

Slot-blot analysis of PAR and PARylated proteins

Cell, ECM, apoptotic body or vesicle lysates were harvested and diluted in TBS. Samples were applied to a Bio-Dot SF Microfiltration Apparatus (Bio-Rad) and blotted onto PVDF membrane that had been pre-soaked in TBS using a Welch Vacuum system (Model 2515) at 5 inHg pressure. Membranes were washed in TBS and then stained with Ponceau S solution and imaged prior to blocking with 5% milk in TBST for and immunodetection of PAR using either 10H anti-PAR antibody or Anti-pan-ADP-ribose binding reagent.

Boronate-IP of PARylated proteins

Lysates were mixed with m-aminophenylboronic acid agarose for 1 hour at room temperature. Following this incubation, beads were washed twice in SDS wash buffer (1% SDS, 100mM HEPES (pH 8.5), 150mM NaCl) and twice in non-SDS wash buffer (100mM HEPES (pH 8.5), 150mM NaCl). Proteins were eluted from beads by boiling in 1 x sample buffer for 10 minutes and analyzed by western blot.

In vitro *VSMC extracellular matrix with ^{13}C, ^{15}N-labeling (Figure S3A)*

Bovine VSMCs were cultured in a T-175 flask containing 25 mL complete Dulbecco's Modified Eagle Medium (DMEM; Invitrogen) with addition of 10% fetal calf serum (First Link), 30 μg/ml L-ascorbic acid 2-phosphate (Sigma) and 10 ml/l L-glutamine-penicillin-streptomycin (200 mM L-glutamine, 10,000 units/ml penicillin, and 10 mg/ml streptomycin in 0.9% sodium chloride; Sigma). All supplements were filter sterilized (0.22 μm filter, Appleton Woods) before addition. After cells were confluent, labeled (U-^{13}C, ^{15}N) glycine (Cambridge Isotope Laboratories), (U-^{13}C, ^{15}N) lysine (Cambridge Isotope Laboratories) and (U-^{13}C) glucose (Cambridge Isotope Laboratories) were added after filter sterilization (0.22 μm) to a final concentration of 60 mg/L, 292 mg/L and 4.5 g/L respectively in the complete medium. The culture was incubated at 37°C in a humidified atmosphere of 95% air and 5% CO_2. The culture medium with isotope-labeled supplements was renewed every 2 days.

When cells produced a dense matrix which started to peel off the surface of the tissue culture flask, the medium was removed and the cells were washed twice with 10 mL phosphate buffered saline (1x PBS, Invitrogen).

Recovery of extracellular matrix

Flasks containing matrix plus cells were placed in a freezer at −80°C for 24 hours and the cells were lysed by thawing the flasks at room temperature for 30 minutes. The debris produced by cell lysis was removed by repeated washes with PBS. The decellularized ECM was dislodged by gently swirling the flask in the presence of 20 mL PBS. The matrix collected in PBS was transferred to a fresh 50 mL centrifuge tube. The ECM was lyophilized overnight. Any adventitious DNA, RNA and PAR was removed by daily incubation with 40 mM aqueous $MgCl_2$ followed by thorough washing, for two days. The samples were stored at −20°C until NMR analysis.

In vitro *fetal sheep osteoblast extracellular matrix with ^{13}C, ^{15}N-labeling (Figure S3B)*

Osteoblasts were cultured to confluence in T-175 flasks containing 25 mL BME complete medium. Labeled (U-$^{13}C5$, ^{15}N) proline (Cambridge Isotope Laboratories) and (U-$^{13}C2$, ^{15}N) glycine (Cambridge Isotope Laboratories) were added after filter sterilization (0.22 μm filter) to a final concentration of 46 mg/L and 30 mg/L respectively and 1g/L of U-^{13}C-glucose (Cambridge Isotope Laboratories). The cultures were incubated at 37°C in a humidified atmosphere of 95% air and 5% CO_2. The culture medium with isotope labeled supplements was renewed every 2 days until the cells and matrix began to detach from the culture flask, by which time enough ECM had formed for SSNMR. Samples from more than 20 batches using the final optimized protocol were prepared using

isotope-enriched amino acids and similarly for samples incorporating U-^{13}C-glucose and all characterized by SSNMR to ensure reproducibility of results.

Recovery of ECM

The matrix was harvested after 9 days of culture, when the cells produce a dense matrix which started to peel off the surface of the tissue culture flask. The medium was removed and the cells were washed with 20 mL 1 x phosphate buffered saline (1X PBS). The flask was placed in a freezer at −80°C for 24 hours and the cells were lysed by thawing the flasks at room temperature for 30 minutes. The debris produced by cell lysis was removed by repeated washes with PBS. The decellularized ECM was dislodged by gently swirling the flask in the presence of 20 mL PBS. The matrix collected in PBS was transferred to a fresh 50 mL tube and centrifuged at 1200 rpm for 5 min at room temperature. The supernatant was poured off and the ECM dehydrated in an oven at 37°C overnight. The samples were stored at −20°C until NMR analysis.

ECM of mouse tissue was used directly in SSNMR experiments without extraction, purification, or excessive processing where possible. Tissues used in each experiment are described below in the SSNMR section.

Solid-state NMR spectroscopy of VSMC and fetal sheep osteoblast extracellular matrix

To prepare the matrix for DNP NMR, the bovine VSMC matrix sample was mixed with D2O/H2O (v/v = 3/1) and a final AMUPol concentration of 10 mM, and packed into 3.2 mm zirconia rotors with a Vespel drive cap. The 2D 13C-13C dipolar-assisted rotational resonance (DARR) correlation spectrum was acquired on a Bruker Avance III NMR spectrometer with a 9.4 T superconducting magnet, equipped with a 3.2 mm triple resonance low temperature DNP-MAS probe and Larmor frequencies of 400 MHz 1H, 100 MHz 13C. The magnet was coupled with a first harmonic gyrotron giving constant microwave irradiation at 263.6 GHz. The sample was cooled in the spectrometer to 100 K, and the NMR experiment conducted at the same temperature with 8889 Hz magic-angle spinning (MAS), using $\nu\leftarrow 1 = \nu R1H$ continuous wave irradiation (DARR) during the mixing period. Parameters used in the NMR experiments were 2 s recycle delay, 4.3 μs 1H 90° pulse length, 1ms cross polarization contact time, 8.5 μs 13C 90° pulse length and the DARR mixing time was 100 ms. 100 kHz SPINAL64 decoupling is applied during both incremental delay and acquisition. Cross polarization used a ramped contact pulse on 13C. Chemical shifts are measured with respect to glycine Cα at 43.1 ppm, which corresponds to the TMS 13C signal being at 0 ppm.

***In vitro*, cell-free nidus calcification models (Figures 4, 5, S4, and S5)**

The *in vitro* calcification experiments with niduses of collagen fibrils, elastin, and DNA in the presence and absence of PAR were carried out at RT in 1.5 mL Eppendorf tubes in a total volume of 100 μl.

Collagen (C-9879; from bovine Achilles tendon), elastin (E-1625; from bovine neck ligament) and DNA (D-1501; from calf thymus) were purchased from Sigma-Aldrich as dry powders and stock solutions were prepared in DIW. Collagen purity was confirmed by amino acid analysis and liquid chromatography (LC)/ mass spectroscopy (MS). Typical analysis:

Asp	4.64
Thr	1.72
Ser	3.24
Glu	7.49
**Gly**	**34.97**
Ala	10.56
Val	2.39
Met	0.07
Ile	1.27
Leu	2.74
Tyr	0.44
Phe	1.45
His	0.51
Lys	2.43
Arg	5.08
**Pro**	**11.42**
**HO-Pro**	**8.96**
**HO-Lys**	**0.63**

Collagen stock solutions were sonicated on ice for at least 30 min prior to use.

Custom-made, EDTA-free PAR was purchased from BioTechne/Trevigen and had a concentration of 100 μM (56 μg/ml; supplied in 10 mM TRIS buffer pH 8.0) and was stored in 50 μL aliquots under liquid nitrogen. Collagen (C-9879; from bovine Achilles tendon),

elastin (E-1625; from bovine neck ligament) and DNA (D-1501; from calf thymus) were purchased from Sigma-Aldrich as dry powders. Collagen, elastin and DNA stock solutions were prepared at 1 mg/ml in DIW, PAR stock was used as provided and microvesicle (MV) preparations (see below) were in 10 mM TRIS buffer pH 8. Collagen stock solutions were sonicated on ice for 30 min prior to use.

Separate Ca^{2+} (11.25 mM $CaCl_2$ x 2 H_2O) and PO_4^{3-} (5.25 mM K_2HPO_4) stock solutions were prepared in 50 mM TBS (7.93 mM TRIS-BASE, 41.9 mM TRIS-hydrochloride, 150 mM NaCl; pH 7.4).

Stock solutions were filter sterilized if possible or contained 0.02% sodium azide in order to avoid bacterial growth. All plastic wares were sterile, and samples were prepared under a sterile work bench. The various nidus additions (see below) were pipetted into Eppendorf tubes in a total volume of 20 μL (negative control - no nidus addition, very little calcium phosphate precipitation even after 14 days; and positive control - addition of pre-formed calcium phosphate leading to extensive calcium phosphate precipitation after 1h - were included in every experiment). Then, 40 μL of Ca^{2+}-stock solution was added, followed by 40 μL of PO_4^{3-} - stock solution; therefore, the Ca^{2+} and PO_4^{3-} concentrations in the assay were 4.5 mM and 2.1 mM, respectively. The tubes were mixed and incubated at RT for the times indicated. Then, samples were centrifuged at 8,000 g for 4 min in a swing-out rotor in a Heraeus table centrifuge temperature-regulated to 20°C. 80 μL of supernatant was removed without disturbing the pellet and the sediments were subsequently washed twice with 200 μL each of DIW (brought to a pH of ~8 by the addition of dilute NaOH in order to avoid dissolution of any formed CaP). After the last centrifugation step, supernatants were removed as much as possible, leaving the sediment in about 20 μL of DIW. Then, samples were re-suspended and applied to TEM sample grids and viewed by BF-TEM as described below. The nidus additions (per 100 μL total sample volume) were as follows:

Sample	Addition 1	Addition 2
negative control	20 μl DIW	-
positive control	1 μg pre-formed CaP	-
collagen	10 μg collagen	-
elastin	10 μg	-
DNA	10 μg DNA	-
PAR	0.56 μg PAR (10 μl stock solution)	
collagen + DNA	10 μg collagen	10 μg DNA
collagen + PAR	10 μg collagen	0.56 μg PAR
elastin + DNA	10 μg elastin	10 μg DNA

Dynamic light scattering

PAR (56 μg/ml PAR stock solution) was mixed with an equal volume of ion solutions (i.e., 2 mM stock solutions of $CaCl_2$, $MgCl_2$, $ZnCl_2$ or $MnCl_2$ in 10 mM TRIS pH 8 to give in-assay concentrations of 1 mM; or 9 mM $CaCl_2$ to give an in-assay concentration of 4.5 mM Ca^{2+}) at RT for about 30 min prior to measurement. DNA was treated with chelex 100 resin for 1h at RT to remove any potential divalent cations present in the commercial preparation and was then used as a control in the same manner (at 40 μg/ml in assay). The hydrodynamic radii of PAR and DNA ± divalent cations were derived from their diffusion coefficients as measured by DLS in optically transparent 96-well plates using a DynaPro Plate Reader (Wyatt Technology), and processed with the associated Dynamics (version 7.1.9) software.

Bright-field transmission electron microscopy (BF-TEM)

For the visualization of PAR in the absence/presence of various divalent cations, PAR (56 μg/ml stock solution) was mixed in equal volumes with 10 mM TRIS buffer pH 8 or with various ions (i.e., with 2 mM stock solutions of $CaCl_2$, $MgCl_2$, $ZnCl_2$ or $MnCl_2$ in 10 mM TRIS pH 8 to give in-assay concentrations of 1 mM ions; or with 9 mM ion stock solution to give an in-assay concentration of 4.5 mM) at RT for about 30 min. DNA was treated with chelex 100 resin for 1h at RT to remove any potential divalent cations present in the commercial preparation prior to incubation with $CaCl_2$ solutions. Then, 5 μL of solution was adsorbed onto glow-discharged 400 mesh copper/carbon-film grids (EM Resolutions) for about 2 min. Grids were rinsed on two drops of DIW and negative staining was performed using a 2% aqueous uranyl acetate solution. Samples destined for energy-dispersive X-ray spectroscopy (EDX) were left unstained.

For the co-incubation of PAR (±ions) with collagen, PAR was incubated first with TRIS buffer or with the indicated concentration of Ca^{2+} or Mn^{2+} ions as described above. Subsequently, an equal volume of collagen suspension (~1 mg/ml in DIW) was added and the mixture was again incubated at RT for the indicated length of time. TEM sample grids were then prepared and left unstained (for EDX) or stained with uranyl acetate as described above. The samples obtained in the in-vitro nidus experiments were left unstained and were viewed by their inherent electron density alone. Five μl of nidus suspensions were applied to 400 mesh copper-holey carbon film grids (EM Resolutions) and allowed to air dry prior to BF-TEM. Grids were viewed in an FEI Tecnai G^2 electron microscope run at 200 kV using a 10 μm objective aperture to enhance contrast. Images were acquired using Deben software. The size of PAR-ion droplets was measured using ImageJ software and size-frequency distributions were prepared using Analyze-it software embedded

in Excel. EDX (energy-dispersive X-ray spectroscopy) was performed on unstained samples using a Peltier-cooled EDAX Ametek window-less octane silicone drift detector and EDAX Genesis software. SAED (selected-area electron diffraction) was performed at 200 kV in diffraction mode at spot size 7 and a camera length of 0.52 m.

In order to assess whether there was preferential localization of PAR-Ca droplets to collagen fibril hole zones, PAR was incubated with 0.5 mM Ca^{2+} for 30 min prior to the addition of collagen for another 30 min. This concentration of Ca^{2+} was chosen as previous experiments had shown that the diameter of the resulting PAR-Ca droplets was about 26 nm, small enough to potentially bind to a single collagen hole zone without overlap. Then, TEM grids were prepared and stained with uranyl acetate as described above. Images of 27 individual collagen fibers incubated with PAR-Ca droplets were acquired.

For each fiber, the total number of bound PAR-Ca droplets was counted, as well as the number of droplets bound to the hole zones. In addition, the total length of the collagen fiber so assessed was measured as well as the length of all the component, individual hole zones. Then, the percentage of PAR-Ca droplets bound to the hole zone was calculated for each image. In total, 992 PAR-Ca droplets were counted over a total length of 39 μm of collagen fibers and the calculation of the cumulative mean gap zone (in %) stabilized after assessing about 20 individual images, making the measurement statistically meaningful.

Elastin/nidus sample embedding for BF-TEM

All TEM processing steps before dehydration of the samples were carried out at pH 8 to ensure that any CaP mineral of the samples would not be dissolved during the procedure. Sediment samples from the *in vitro* nidus model were mixed with an equal volume of 20% gelatine (Sigma G-2500; in 0.05 M sodium cacodylate buffer pH 8) warmed to about 50°C, quickly transferred onto glass slides and allowed to set at 4°C. The set gelatin was cut into fine pieces using a razor blade and transferred into fixative (2% glutaraldehyde/ 2% formaldehyde in NaCAC) and fixed overnight at 4°C. Fixative was removed by washing 5x in 0.05% sodium cacodylate buffer pH 8 (= 'NaCAC') and the samples were osmicated (1% osmium tetroxide/1.5% potassium ferrocyanide in NaCAC) for 3 days at 4°C. After several washes (NaCAC), samples were treated for 20 min at RT with 1% thiocarbohydrazide/DIW and then washed again using NaCAC. Subsequently, samples were osmicated a second time for 1h (2% osmium tetroxide in NaCAC). Samples were then washed 4x in DIW (brought to pH of about 8 by addition of dilute NaOH) before being dehydrated in 50/70/95/100% ethanol (at least 3x for 5 min in each). Then, samples were bulk stained overnight using 2% uranyl acetate/100% methanol. Samples were washed several times in dried, 100% ethanol and then further dehydrated using dry acetone and dry acetonitrile (at least 3 exchanges in each solvent). Samples were then incubated in 50% acetonitrile 50% Quetol 651 epoxy resin overnight. This operation was followed by four daily changes of 100% resin. The resin mixture contained: 12 g Quetol 651 (Agar), 15.7 g Nonenyl Succinic Anhydride (NSA) hardener, 5.7 g methyl-5-norbornene-2,3-dicarboxylic anhydride (MNA) hardener and 0.5 g benzyldimethylamine (BDMA) catalyst. They were then cured at 60°C for 48 h. Elastin - as supplied by Sigma - was embedded using the above method except that the bulk stain was carried after the second osmication step using 2% uranyl acetate in 0.5 M maleate buffer pH 5.5 for 3 days at 4°C; instead of the bulk stain in methanol as described above - and washing steps were performed using DIW).

Thin sections (60-90 nm) were cut using a Leica Ultracut UCT ultramicrotome and collected on bare 300 mesh copper grids and were not post-stained. Grids were viewed using an FEI Tecnai G2 in bright field mode operated at 200 kV and using a 10 μm objective aperture to improve contrast.

Block-face SEM imaging of warfarin-induced vascular calcification in rats

Aorta were dissected after 7 days and fixed by immersion in 10% acrolein in anhydrous methanol for 12 hours. They were rinsed x10 in anhydrous methanol, and twice in acetonitrile before embedding in Quetol 651 as described above. Cross sections of embedded aortae were glued to Leica cryo-pins with conducting epoxy resin. The aortae were sectioned with glass knives in a Huxley mark II ultramicrotome until a complete transverse profile was revealed. The pins were removed and the blocks and pins were coated with 70 nm of gold in an Emitech sputter coater. They were returned to the ultramicrotome and a mirror surface was produced by sectioning dry with a Dupont diamond knife. The blocks were then coated with 30nm of carbon in a Quorum K150 evaporative carbon coater. They were viewed in an FEI Verios 460L SEM using all six segments of a concentric backscattered detector, operated in full immersion mode, at an accelerating voltage of 20 kV and a probe current of 400 pA. EDX spectra were collected for 100 s live time using a 60 mm^2 Silicon Drift Detector in an EDAX spectrometer running Genesis software.

Effect of PARP inhibitors on *in vitro* human VSMC calcification (Figures 6 and S6)

RNA isolation, reverse transcription, and real-time PCR for hVSMCs

Cells were lysed in RNA STAT-60 (Amsbio) according to manufacturer's protocol. NanoDrop ND-1000 spectrophotometer was then used to measure the concentration of isolated RNA. Reverse transcription of RNA was carried out with Random primers (Promega), Oligo dT primers (Promega), dNTP mix (Eurogentec), RNAsin RNase inhibitor (Promega), 5x Mu-MLV buffer (Eurogentec), Mu-MLV reverse transcriptase (Eurogentec) and DEPC H_2O. The RNA and primers were incubated at 65°C for 5 minutes prior to the reaction to denature the RNA. The reverse transcription reaction was carried out at 25°C for 10 minutes for primers to anneal and then 37°C for 50 minutes for the reverse transcription to produce cDNA. A final incubation at 95°C for 5 minutes terminated the reaction and inactivated reverse transcriptase. The reaction was then cooled to 4°C in an Applied Biosystems 2720 Thermal Cycler. The resulting cDNA was diluted to 2 μg/100 μL in DEPC H_2O.

Real-time qRT-PCR was carried out in 20 μL reactions with 2 × MESA GREEN qPCR MasterMix at a final concentration of 0.125 μM of each primer in a Corbette Rotor Gene 3000. 40 cycles of 95°C for 15 s and 60°C for 60 s were carried out, with melt curve analysis step at the end. The expression of all genes was quantified using $2^{-\Delta\Delta}$Ct Method, with GAPDH used as an internal control.

The validated primers used for this method is as follows; PARP1: PP00686B (QIAGEN); PARP2: PPH02684F (QIAGEN); BMP2: QT00012544 (QIAGEN); OCN: F:GGCAGCGAGGTAGTGAAGAG R: CGATAGGCCTCCTGAAAGC (Integrated DNA Technologies); MSX2: F: AAATTCAGAAGATGGAGCGGCGTG R: CGGCTTCCGATTGGTCTTGTGTTT (Integrated DNA Technologies); SMA: F:TTG AAGGCAAAGACATGGCAGCAG R:TCCACGGTAGTGCCCATCATTCTT (Integrated DNA Technologies); SM22: F:TTGAAGGCAA AGACATGGCAGCAG R:TCCACGGTAGTGCCCATCATTCTT (Integrated DNA Technologies); GAPDH: F:CGACCACTTTGTCA AGCTC R:CAAGGGGTCTACATGGCAAC (Integrated DNA Technologies).

RNA isolation, reverse transcription, and real-time PCR: MC3T3-E1 cultures

The cells were harvested at day 12 and total RNAs were isolated from cultures by RNeasy mini kit according to the manufacturer's instructions (QIAGEN) and quantified using NanoDrop (Thermo). First-strand cDNA was synthesized from 1 μg of total RNA using RT^2 First Strand Synthesis kit (QIAGEN). Typical reaction mix consists of SYBR green reaction master mix (12.5 μl), gene specific primer (1 μM), diluted cDNA (15 ng) and nuclease-free water in a total reaction volume of 25 μl.

The temperature cycling conditions for RT PCR consists of an initial denaturation step at 95°C for 30 s followed by 40 cycles of 95°C, 10 s and 60°C, 30sec. RT-PCR was carried out using a Bio-Rad CFX96 Touch Real-Time PCR Detection System (Bio-Rad). GAPDH was used as the reference gene. Samples were run in triplicate and data was analyzed using CFX Maestro Software (Bio-Rad).

Validated gene (mouse) specific primer sets for use in SYBR Green-based real-time RT-PCR were procured from QIAGEN (QuantiTect Primer Assay) and the primer code for the genes are as follows; Osterix (Osx): QT00293181 1 Mm_Sp7_1_SG QuantiTect Primer Assay; Type 1 collagen alpha chain: QT00162204 1 Mm_Col1a1_1_SG QuantiTect Primer Assay; Osteopontin: QT00157724 1 Mm_Spp1_1_SG QuantiTect Primer Assay; Osteocalcin: QT00259406 1 Mm_Bglap_1_SG QuantiTect Primer Assay; Osteonectin: QT00161721 1 Mm_Sparc_1_SG QuantiTect Primer Assay; Alkaline phosphatase: QT00157717 1 Mm_Alpl_1_SG QuantiTect Primer Assay; GAPDH: QT01658692 1 Mm_Gapdh_3_SG QuantiTect Primer Assay; PARP1: QT00157584 1 Mm_Parp1_1_SG QuantiTect Primer Assay and PARP2: QT00162281 1 Mm_Parp2_1_SG QuantiTect Primer Assay.

Alizarin red staining of calcification

The cells were washed twice with phosphate-buffered saline (PBS) and then fixed with 4% paraformaldehyde (PFA) for 10 minutes at room temperature. Following this, the cells were washed twice with dH_2O and stained with 2% Alizarin red for 5 minutes and rinsed with water.

Cresolphthalein assay of calcification

Human VSMCs were plated with duplicates for each condition, so that one well would serve to provide protein lysates for normalization. The cells were washed twice with Hank's Balanced Salt Solution (HBSS, Trevigen), followed by overnight incubation at 4°C in 100 μL 0.1M HCl to dissolve the mineral deposits, or in 100 μL 0.1M NaOH/1% SDS to lyse the cells for protein normalization. On the following day, 5 μL of each HCl decalcified sample were transferred to a 96-well plate and mixed with 75 μL of dH_2O before the addition of 10 μL of o-cresolphthalein solution (5 mg o-cresolphthalein complexone in 3.6 mL dH_2O + 1.4 mL ammonia buffer) and 200 μL ammonia buffer (5 mL ammonium hydroxide, 0.24 g ammonium chloride in 100 mL dH_2O pH 10.5). Serial dilutions of $CaCl_2$ were made to form a standard curve and the absorbance was measured at 540 nm using TECAN Genios Pro multifunction microplate reader. Calcium concentrations were calculated and normalized to protein concentrations determined using DC Protein Assay (Bio-Rad) with absorbance measured at 710nm using Tecan Genios Pro multifunction microplate reader.

PARP activity assay in cells

The PARP activity of hVSMCs was tested with an HT colorimetric PARP/apoptosis assay kit (4684-096-K; Trevigen), according to the manufacturer's protocol. After calcification treatments of hVSMCs, the cells were lysed in cell extraction buffer prepared from the kit. Equal amounts of protein extracts were then incubated with PARP substrate cocktail together with histone-coated strip wells. The poly (ADP-ribose) on the strip wells was then detected by monoclonal anti-poly (ADP-ribose) antibody and HRP-conjugated goat anti-mouse IgG antibody, with washing for each step. TACS-Sapphire colorimetric substrate from the kit was then added. Absorbance was read at 450 nm using BioTek ELx800 multifunction microplate reader.

Cell vitality assay

The cell vitality of hVSMC was measured by Solution 5 VB-48-PI-AO (910-3005; Chemometec) using the NucleoCounter® NC-3000 system, according to the manufacturer's protocol. After calcification treatments of VSMCs, the cells were trypsinized and resuspended with the appropriate amount of medium. Following this step, cell sample were added into microcentrifuge tube (one volume of solution 5 into 19 volumes of cell suspension). Then mixed sample was added on a 2-chamber slide or an 8-chamber slide depending on the number of cells. Lastly, the assay was immediately performed using NucleoCounter® NC-3000.

PARP inhibitors

Inhibitor stock solutions were made in DMSO (Sigma) at 10 mM. For the inhibitor experiments, the VSMCs were treated with either DMSO (vehicle), the PARP inhibitors PJ34 (AdipoGen), Minocycline (Cycle Pharm), Olaparib, Rucaparib, Niraparib, Veliparib (LGM Pharma), or with the PARG inhibitor DEA (6,9-diamino-2-ethoxyacridine-DL-lactate) (Sigma) at indicated concentrations diluted in the same medium used in the high Ca/P media, GAD-medium, control media or serum-free DMEM media.

PARP enzymatic activity assay with inhibitors

PARP activity and inhibition was measured for recombinant human full length active PARP1 and PARP2 enzymes (ab79663 and ab198766 respectively, Abcam) according to the kit description (Merck Millipore, 10149) with some alterations. Inhibitor compounds were added to the reaction buffer, prepared as a 1:1 mixture of Merck kit buffer with 50mM Tris-HCl, 100 mM NaCl, 5mM $MgCl_2$,

0.05% Tween-20, pH 8.0 (Sigma), and pre-incubated with PARP1 (2.5 ng/μL final) or PARP2 (12ng/μL final) at room temperature for 30 min. Further steps were performed following the kit instruction and fluorescence measurement of a product derived from nicotinamide (itself product of PARP reaction) was carried out at 420 nm excitation and 460 nm emission in FLUOstar Omega platereader (BMG Labtech). Reaction sample without inhibitor was considered as 0%, and without NAD+ was considered as 100% inhibitory value.

Rat CKD model

After isolation of the proximal part of the thoracic aorta, the tissue was fixed in neutral buffered formalin for 90 minutes and cut into sections of 2-3 mm. These 15-20 sections were embedded upright in a paraffin block and 4 μm sections were stained for calcification with Von Kossa's method and counterstained with hematoxylin and eosin.

The % of calcified area was calculated using Axiovision image analysis software (Release 4.5, Carl Zeiss, Oberkochen, Germany) in which two color separation thresholds measure the total tissue area and the Von Kossa positive area. After summing both absolute areas, the % of calcified area was calculated as the ratio of the Von Kossa positive area versus the total tissue area. The proximal part of the abdominal aorta and the left carotid and femoral artery were isolated and weighed on a precision balance. Subsequently, the samples were digested in 65% HNO_3 at 60°C for 6 hours. The calcium content of each artery was measured with FAAS and expressed as mg calcium/g wet weight tissue.

The study of PAR, γH2AX and αSMA expression was performed using immunohistochemistry of these rat thoracic aortas. After deparaffinization and rehydration, the sections were incubated in citric acid based antigen retrieval buffer for 25 min and then left to cool down. These slides were then immerged in 3% H2O2 prepared in methanol to avoid any endogenous peroxidase activity. After washing, the aortic sections were blocked in 10% blocking serum for 30min. The primary antibodies were diluted in blocking serum and slides were left overnight at 4oC. Following washing, these sections were incubated with biotinylated secondary antibodies diluted in blocking serum for 1h at RT. Subsequently, the slides were incubated with ABC reagent (kit) for 30 min. DAB peroxidase substrate kit was used to develop the appropriate color and sections were counterstained with hematoxylin. The slides were mounted using DPX following dehydration.

Images were taken using Leica ICC50 W microscope. The γH2AX expression was quantified using ImageJ software by counting γH2AX positive (brown) and negative (purple) number of cells. After calculating the total number of cells, the % of γH2AX cells was calculated as the ratio of γH2AX positive versus the total number of cells.

Scanning electron microscopy (SEM) and transmission electron microscopy (TEM)

Rats femurs were removed after animals were sacrificed. After de-fleshing, the bones were preserved in 100% ethanol until further analysis. In order to obtain cross-sections for SEM, the femurs were plunge-frozen and cleaved at liquid nitrogen temperatures at both ends, where the long-bone section starts to transition into the joint area. Samples were freeze-dried in a liquid nitrogen-cooled Quorum Emitech K775X turbo freeze-dryer and mounted on aluminum SEM stubs using double-sided sticky tape. Samples were made conductive by painting with Ag-DAG electropaint and were coated with 35 nm gold/15 nm iridium prior to viewing in a FEI Verios 460 SEM. SE images were taken at 2 keV accelerating voltage and 25 mA probe current using the EDT detector; high resolution images were obtained using the TLD detector in full immersion mode. Large area maps of the cross sections were obtained using the FEI MAPS automated image acquisition software. ImageJ software was used to evaluate the extent of bone remodelling in the rat femur samples (see image). First, the counting area of the cross-section was delineated by marking the outside (green line) and inside border (orange line) of the cortical bone area; then, a square lattice was superimposed and the number of grid intersections falling on areas of solid bone (black dots) and areas of bone remodelling (purple dots) were counted. The area fraction of solid bone in the cortical area was calculated as: Area fraction of solid bone (in %) = 100 * number of solid bone intersections/total number of area intersections.

QUANTIFICATION AND STATISTICAL ANALYSIS

Results are presented as mean ± SEM unless stated otherwise. Statistical analysis was performed with GraphPad software. All the data was tested for normalcy using the Shapiro-Wilk test. The comparisons were made using non-parametric Kruskal Wallis tests for all studied groups; if the results were significant, each independent group was compared by Mann-Whitney U test. The parametric t test was assessed in case of normal distribution between 2 independent groups or with 1-way ANOVA with Dunnett's post hoc test. For comparisons of multiple groups 2 say ANOVA with Turkey's test was used.

Rat CKD model (Figures 7 and S7)

Results are expressed as mean ± SEM unless otherwise indicated. Non-parametric statistical analyses were performed with SPSS 24.0 software. As many study groups have been included in this study, multiple statistical tests have been performed.

(i) To determine differences at each time point and for each parameter between the rats with normal renal function (NRF + vehicle) and the CKD groups, a Kruskal-Wallis (KW) test was assessed including all study groups. If the KW test showed significance, a Mann-Witney U test was performed to compare differences between 2 independent groups; to this end, all CKD groups were compared with the NRF + vehicle group in order to test whether differences between each CKD group and the control group

(NRF + vehicle) were present. Bonferroni correction was applied to correct for multiple comparisons. A value of $p < 0.05$ was considered significant.

(ii) To determine differences at each time point and for each parameter between the CKD groups treated with minocycline for 6 weeks versus the CKD group treated with vehicle for 6 weeks, a KW test was assessed including the CKD + vehicle 6 wks, CKD + MC/05 6 wks, CKD + MC/10 6 wks and CKD + MC/50 6 wks groups. If the KW test showed significance, a Mann-Witney U test was performed to compare differences between 2 independent groups; to this end, all CKD + MC study groups were compared with the CKD + vehicle group in order to test whether differences between each minocycline treated CKD group and the vehicle treated CKD group were present. Bonferroni correction was applied to correct for multiple comparisons. A value of $p < 0.05$ was considered significant.

Cell Reports, Volume *27*

Supplemental Information

Poly(ADP-Ribose) Links
the DNA Damage Response
and Biomineralization

Karin H. Müller, Robert Hayward, Rakesh Rajan, Meredith Whitehead, Andrew M. Cobb, Sadia Ahmad, Mengxi Sun, Ieva Goldberga, Rui Li, Uliana Bashtanova, Anna M. Puszkarska, David G. Reid, Roger A. Brooks, Jeremy N. Skepper, Jayanta Bordoloi, Wing Ying Chow, Hartmut Oschkinat, Alex Groombridge, Oren A. Scherman, James A. Harrison, Anja Verhulst, Patrick C. D'Haese, Ellen Neven, Lisa-Maria Needham, Steven F. Lee, Catherine M. Shanahan, and Melinda J. Duer

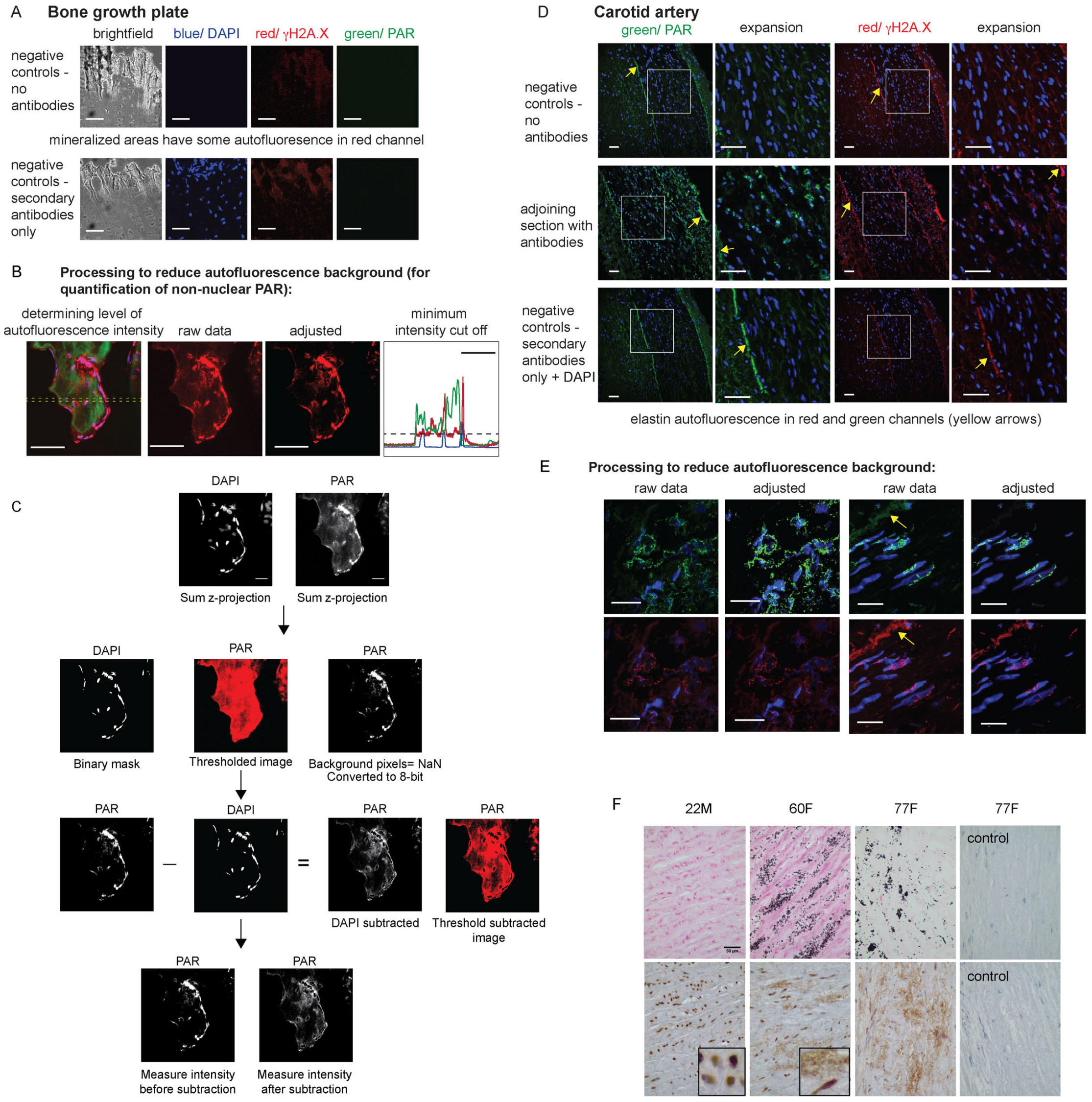

Figure S1: Controls and image processing for Figure 1

(A) Negative controls for confocal imaging of bone growth plate in Fig 1.

(B) Image processing for bone growth plate images in Fig 1A, B.

(C) Details of image processing and quantification method for determining fraction of extranuclear PAR from confocal images of mineralized and non-mineralized areas of bone growth plate for results shown in Fig 1A.

(D) Negative controls for carotid artery confocal imaging in Fig 1 (maximum intensity projections of z-stacks). Yellow arrows indicate autofluorescence from elastin.

(E) Image processing for vessel images in Fig 1C. Images in (A) – (E) are maximum intensity projections of z-stacks; scale bars, 50 μm.

(F) Examples of the immunohistochemistry images used to quantify ratio of extranuclear/ nuclear PAR and extent of vessel calcification in Fig 1D.

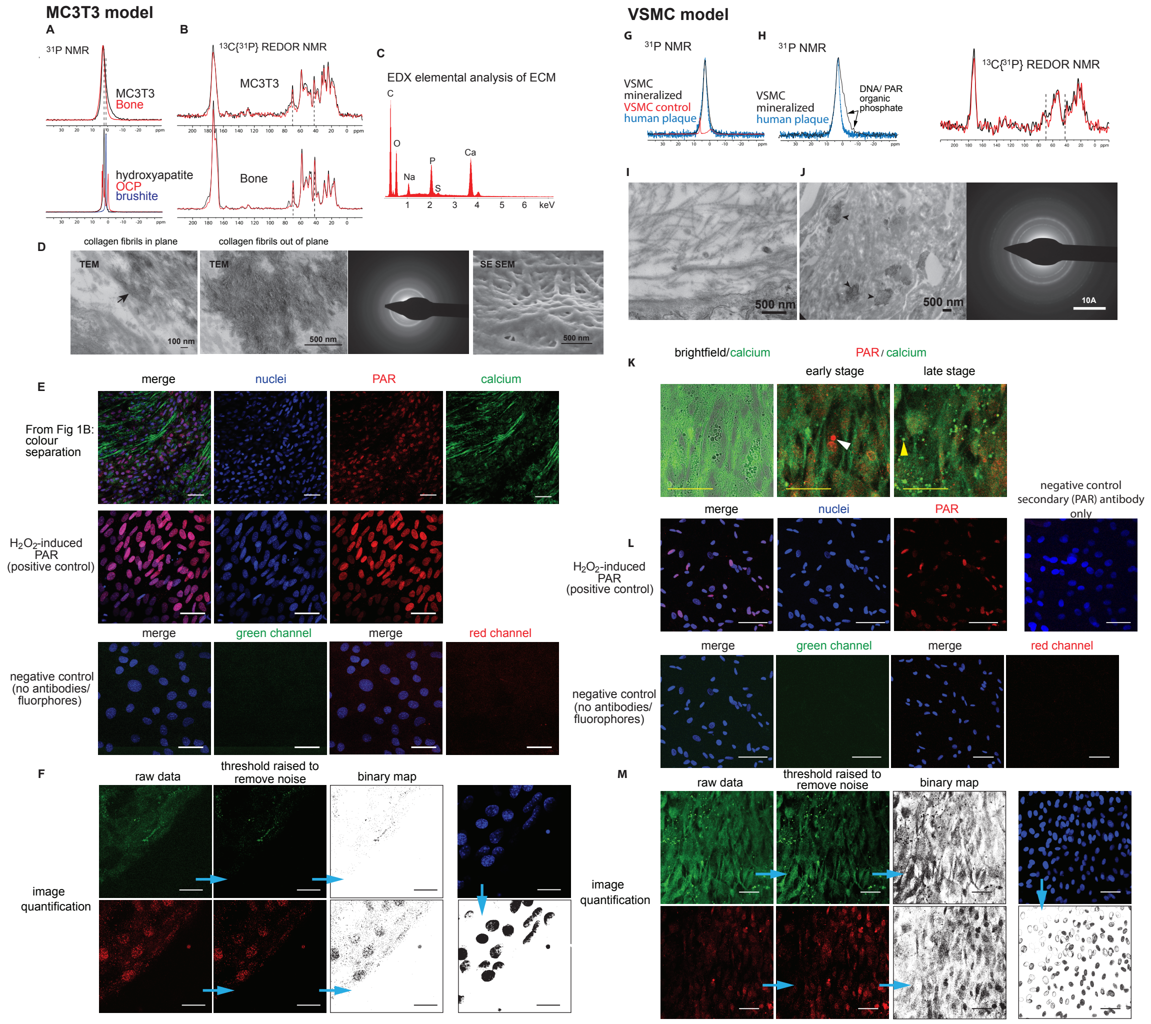

Figure S2: Supporting data for MC3T3 and b/h VSMC in vitro models of extracellular calcification used in Figure 2.

MC3T3 model

(A) ^{31}P direct polarization, magic-angle spinning (DP-MAS) NMR spectra of MC3T3 mineralized matrix compared with native mouse bone and pure calcium phosphate phases, showing that the mineral in the MC3T3 model is bone-like and apatitic in form.

(B) ^{13}C{^{31}P}Rotational Echo Double Resonance (REDOR) NMR spectra of MC3T3 mineralized matrix and native mouse bone. Black – reference spectrum, red = REDOR-dephased spectrum. Signals from collagen triple helix hydroxyproline (Cγ, 70 ppm) and glycine (Cα, 42.5 ppm) and indicated with dotted lines. Decreased intensity in the REDOR (red) spectrum compared to reference (black) are from ^{13}C sites within 1 nm of ^{31}P, i.e. close to phosphatic mineral. Similarities in dephasing pattern between MC3T3 and bone REDOR spectra indicate similarity in mineral-organic matrix interactions.

(C) EDX element composition analysis of MC3T3 mineralised matrix from TEM in (D).

(D) Electron microscopy assessment of mineral morphology and spatial distribution in MC3T3 mineralized matrix, showing mineral aligning with collagen fibrils (fibrils in plane) and curving around fibrils (fibrils out of plane) similarly to native bone (*47*).

(E) Controls for confocal imaging in Fig 2B – E. Scale bars, 50 μm.

(F) Details of image processing for quantification of fraction of extracellular PAR. Scale bar, 20 μm.

VSMC models.

(G), (H) ^{31}P direct polarization, magic-angle spinning (DP-MAS) NMR spectra of (G) bVSMC and (H) hVSMC mineralized matrix, compared with calcified human carotid calcification. (H) also shows a REDOR $^{13}C\{^{31}P\}$ NMR spectrum for hVSMC calcified matrix. Signals from collagen hydroxyproline and glycine (dotted lines, see (B) for details and comparison) are typically weak in these samples.

(I), **(J)** TEM images of bVSMC extracellular matrix under (I) non-calcifying and (J) calcifying conditions. Mineralized matrix shows patches of mineral (arrowheads) a few microns in dimensions. SAED of these patches (right) indicates crystalline material with multiple crystallite orientations.

(K) Expansions of areas in the confocal images in Fig 2G, H, showing PAR containing vesicles (red/ orange, white arrowhead) and mineralized patches (green, yellow arrowhead) typically a few microns in diameter. Controls for confocal imaging in Fig 2G – J.

(M) Details of image processing for quantification of fraction of extracellular PAR.

Scale bars, 50 μm for (K) – (M).

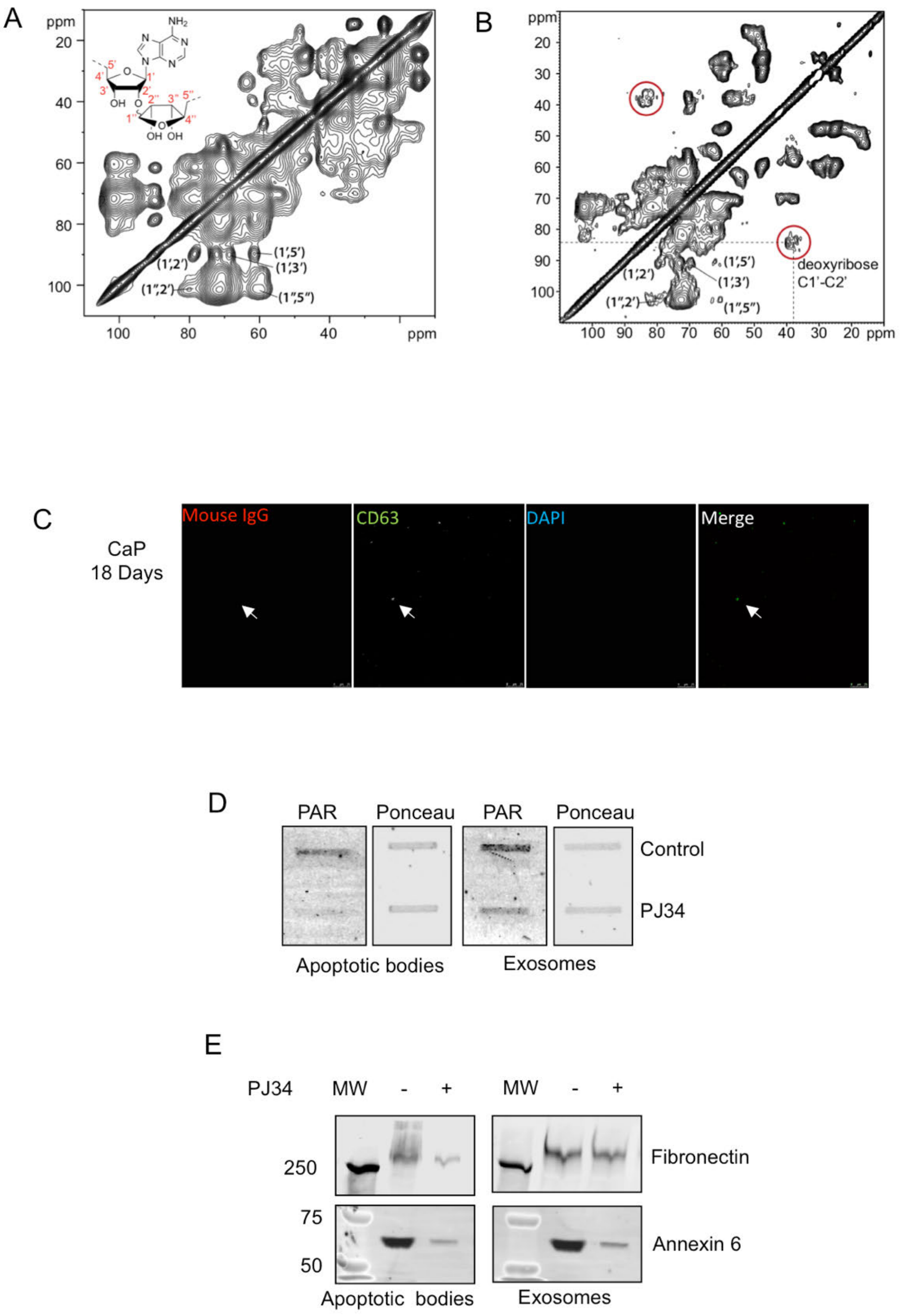

Figure S3: Supplemental data for Figure 3

(A-B) 2D ^{13}C-^{13}C proton-driven spin-diffusion correlation NMR spectra of in vitro ECM from cell cultures supplemented with U-^{13}C-glucose for **(A)** bVSMC and **(B)** foetal sheep osteoblasts. The spectra show signals only from parts of molecules that have been ^{13}C-labelled from the U-^{13}C-glucose added to the cell culture medium during growth of the ECM. Ribose rings and collagen native glycosylation are the main components labelled in these spectra. The signal frequencies (denoted as chemical shifts) depend on the molecule the ribose ring is in. Thus, signals from ribose rings in PAR or in DNA appear at distinctive and different frequencies. The signal frequencies for PAR and DNA are established from NMR spectra of the pure materials (Chow *et al.*, 2014).
(C) Absence of DAPI staining confirming complete cell lysis and removal of nuclear material. IgG antibody controls for PAR are negative.
(D) Protein slot blot analysis of PAR content in VSMC-derived EV populations isolated by differential ultracentrifugation (n = 3). Note inhibition with PJ34.
(E) Boronate bead enrichment for PAR and western blot of EVs shows decreased binding of fibronectin and annexin 6 to the beads after PJ34 treatment (n = 2).

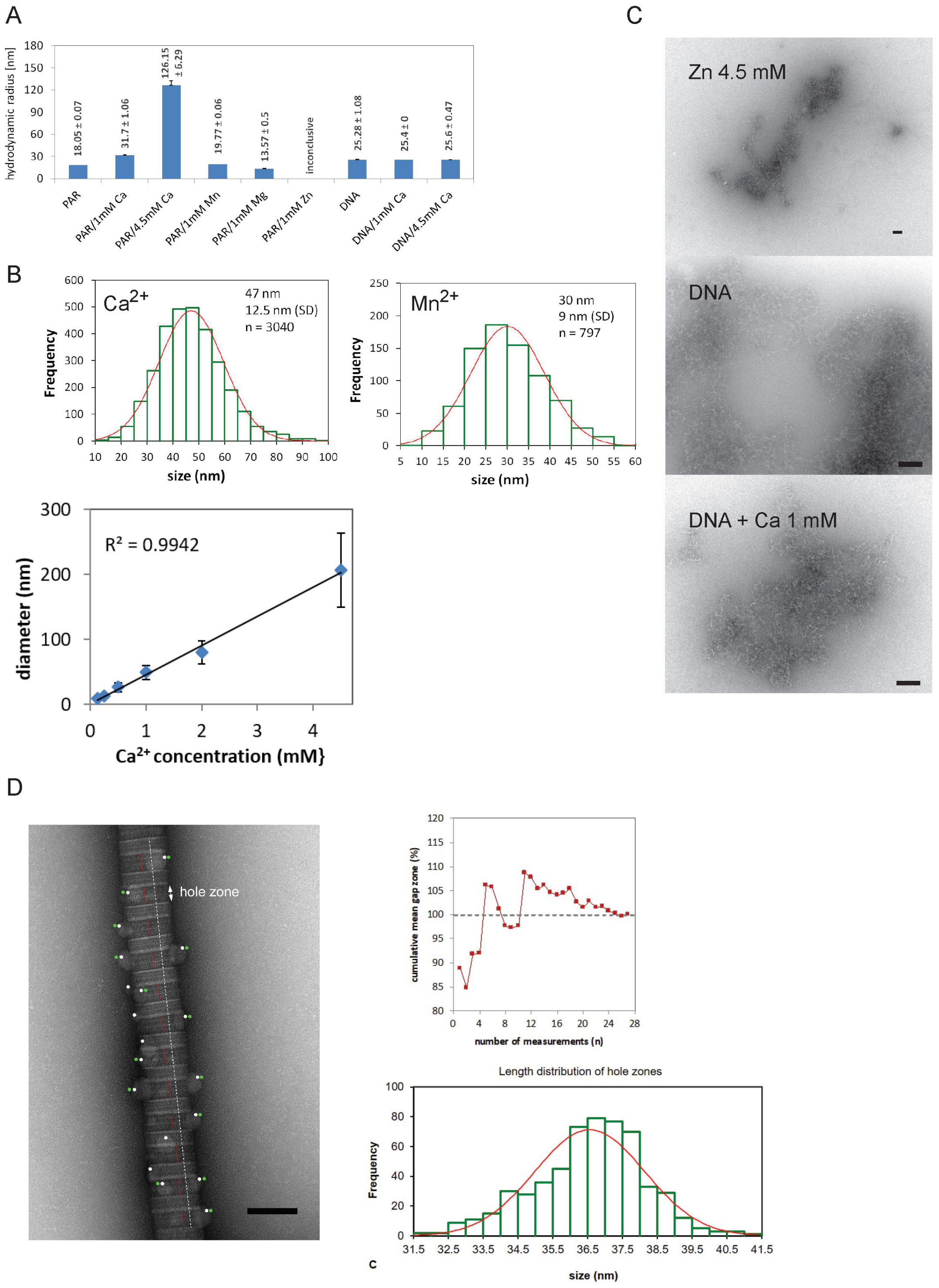

Figure S4: Data analysis and additional data/ controls for Figure 4.

(A) Hydrodynamic radii of PAR in the absence and presence of various divalent cations as measured by DLS; DNA was used as a control. Values given are the mean ± SD.

(B) Graphs showing the size-frequency distributions of PAR spheres after addition of 1 mM calcium and 1 mM manganese, respectively. The graph below indicates a linear trend in the tested concentration range with a highly significant R^2.

(C) TEM images of PAR + 4.5 mM Zn^{2+} (showing absence of spheres), and as controls, DNA alone and DNA + 1 mM Ca^{2+}.
(D) Details of how PAR-Ca sphere localization on hole/ overlap zones of collagen fibrils was determined. Left: Example TEM image: all PAR-Ca spheres bound to the collagen fibril were labeled with a white dot (irrespective of their location); the PAR-Ca spheres bound to the fibril hole zones were labeled with a green dot. The length of the fibril was measured along a certain number of D-periods (white line), as well as the length of the individual hole zones (red lines). Graph (right, top) plotting the cumulative mean gap zone in % with increasing number of measurements (n = 27 images). After assessing about 20 images, the cumulative mean gap zone stabilizes towards 100 %, indicating that a statistically sufficient number of measurements have been performed. Right, bottom: Length-frequency distribution of the measured hole zone lengths (mean ± SD). Scale bars 100 nm for (B) – (D).

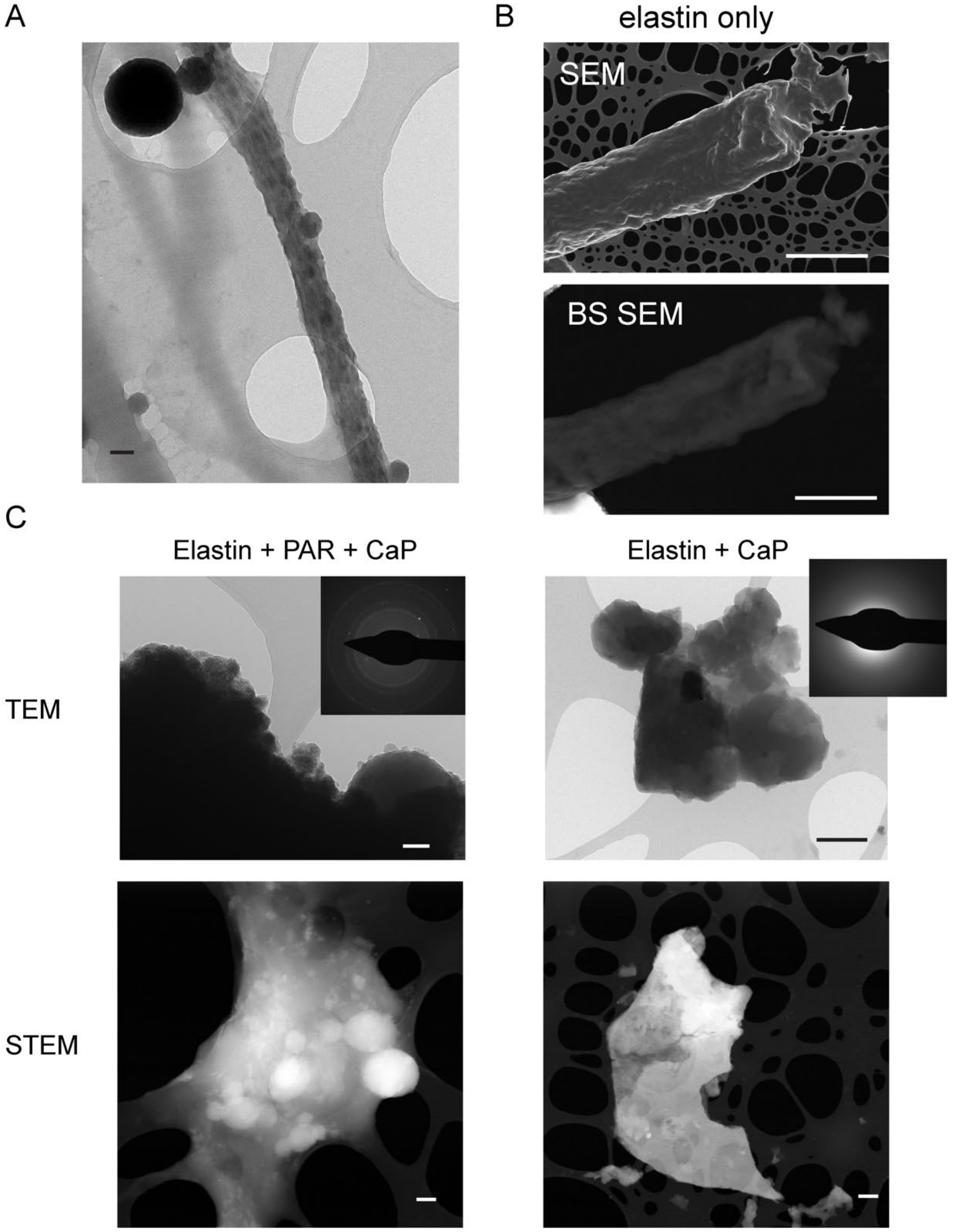

Figure S5: Additional data and controls for Figure 5

(A) TEM image of collagen fibril after 10 days of incubation with PAR and 4.5 mM Ca^{2+} ($CaCl_2$), 2.1 mM PO_4^{3-} (K_2HPO_4) TRIS buffered solution showing mineral formation on the fibril and spheres binding/ adjacent to the calcified fibril. Scale bar, 100 nm.

(B) SEM images of elastin only as controls for the SEM image in Fig 6C. Scale bars, 5 μm.

(C) Additional TEM and STEM images for elastin + PAR (left, scale bars 100 nm) and elastin alone (right, scale bars, 500 nm) after incubation with 4.5 mM Ca^{2+} ($CaCl_2$), 2.1 mM PO_4^{3-} (K_2HPO_4) TRIS buffered solution.

A

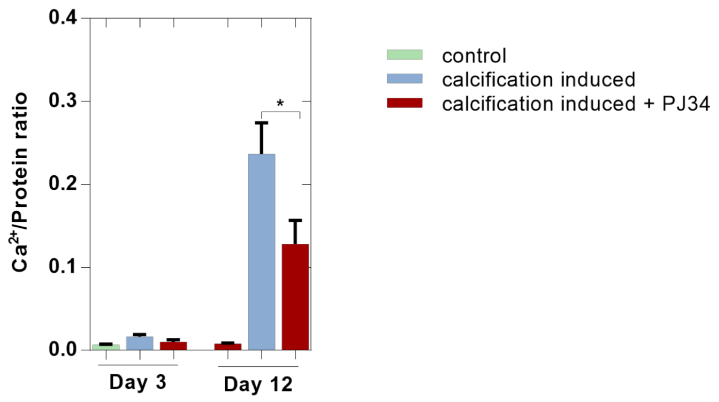
0.4
0.3
0.2
0.1
0.0
Ca^{2+}/Protein ratio
*
Day 3
Day 12
control
calcification induced
calcification induced + PJ34

B

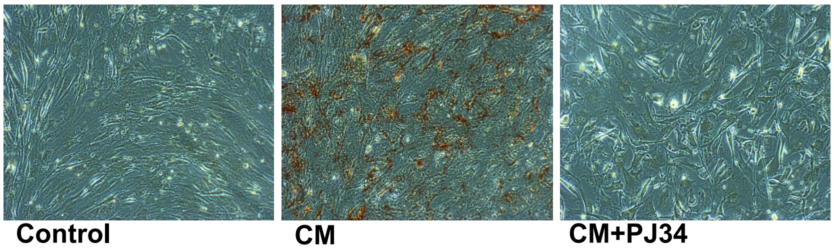
Control
CM
CM+PJ34

C

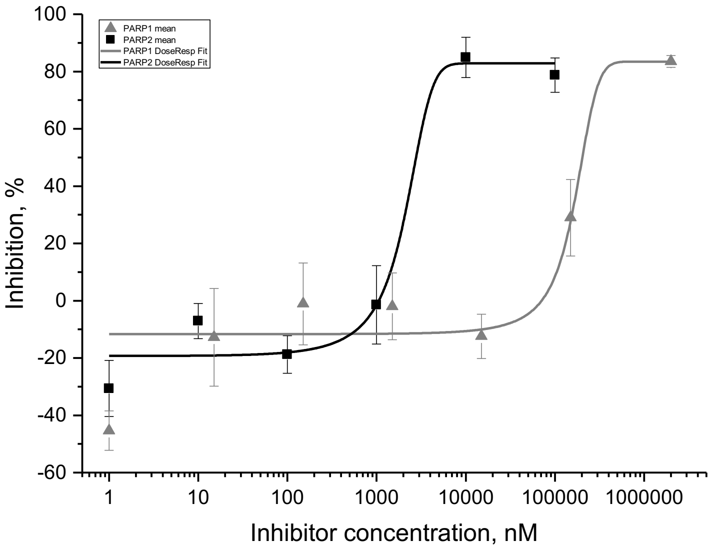
PARP1 mean
PARP2 mean
PARP1 DoseResp Fit
PARP2 DoseResp Fit
100
80
60
40
20
0
-20
-40
-60
Inhibition, %
1
10
100
1000
10000
100000
1000000
Inhibitor concentration, nM

Figure S6: The effect of PARP inhibitor PJ34 in the bovine VSMC (bVSMC) model as additional data for Figure 6 and the inhibitor effect of minocycline on PARP1/2 activity to support the data in Figures 6F – H.

(A) The PARP inhibitor PJ-34 (10 mM) inhibited calcification in bVSMCs induced to mineralize by treatment with 10 mM ß-glycerophosphate (ßGP) + 0.1 mM ascorbic acid 2-phosphate + 10 nM dexamethasone (CM) (n = 3).

(B) Mineralization in the bVSMC cultures was visualized with Alizarin Red S staining

(C) Dose-response curve for minocycline against human PARP1 and 2. Minocycline is a selective inhibitor of PARP2. In *in vitro* PARP activity assay minocycline inhibits PARP2 and PARP1 activity with an IC50 of 2.8 μM and 204.5 μM correspondingly.

A

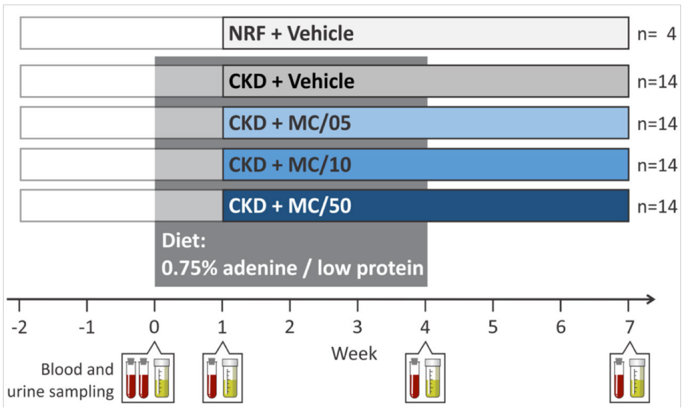

B

Biochemical Parameters		NRF + vehicle	CKD + vehicle	CKD + MC/50
**Creatinine Clearance** ml/min/100g body weight	**Baseline**	0.425 ± 0.054	0.523 ± 0.020	0.545 ± 0.021
	**Week 1**	0.301 ± 0.020	0.189 ± 0.017[a]	0.199 ± 0.019[a]
	**Week 4**	0.330 ± 0.005	0.039 ± 0.003[a]	0.034 ± 0.003[a]
	**Week 7**	0.330 ± 0.016	0.083 ± 0.008[a]	0.056 ± 0.005[a,b]
**Serum Phosphorus** mg/dl	**Baseline**	9.23 ± 0.80	9.83 ± 0.44	11.49 ± 0.29[a]
	**Week 1**	8.27 ± 0.73	10.07 ± 0.50	9.60 ± 0.84
	**Week 4**	10.61 ± 1.36	25.08 ± 0.86[a]	25.18 ± 0.91[a]
	**Week 7**	7.32 ± 0.64	11.05 ± 0.51[a]	12.35 ± 0.58[a]
**Serum Calcium** mg/dl	**Baseline**	11.61 ± 0.42	11.02 ± 0.22	9.07 ± 0.17[a,b]
	**Week 1**	8.46 ± 0.19	7.77 ± 0.11[a]	6.94 ± 0.07[a,b]
	**Week 4**	8.30 ± 0.17	6.82 ± 0.19[a]	6.28 ± 0.18[a]
	**Week 7**	7.02 ± 0.16	5.84 ± 0.35	4.84 ± 0.33[a]

Renal function and mineral metabolism. Creatinine clearance, serum phosphorus and calcium values (mean ± SEM) throughout the study course. [a]p<0.05 versus NRF + vehicle group. [b]p<0.05 versus CKD + vehicle. NRF: normal renal function. CKD: chronic kidney disease. MC/50: daily treatment with 50 mg/kg minocycline.

C

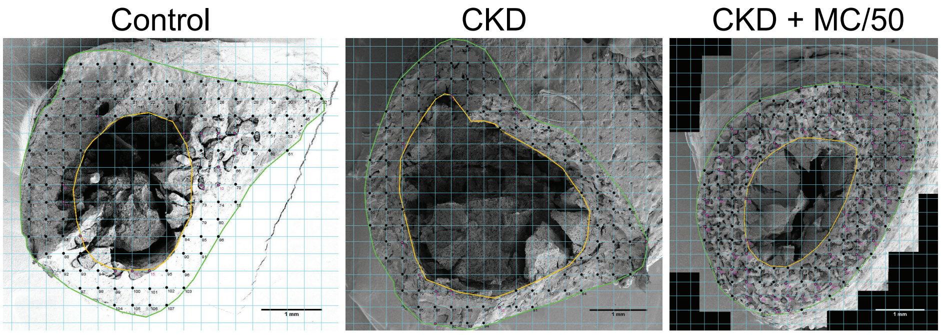

Figure S7: Further information on the CKD in vivo model used in Figure 7.

(A) Cartoon showing the study protocol for the rat CKD model.

(B) Table showing the biochemical parameters for renal function and mineral metabolism in the rat CKD model during the 6 week induction period.

(C) Details of the grids used to estimate the degree of calcification in each bone sample. Scale bars, 500 μm.

Table S1: Details of the blood vessel samples used in extranuclear PAR and calcification content analysis in Figure 1.

n	Age/Sex/Vessel	Calcification	PAR Localization		Ratio
		(%)	Nuclear	Cytoplasmic/Extracellular	ExtraC/Nuclear
1	16F,26A	0.2	100	0	0.0
2	18M,9A	0	100	0	0.0
3	22M,2A	0	100	0	0.0
4	52M,3A	0.4	75	25	0.3
5	53F,4A	1.8	75	25	0.3
6	53F,8A	0.8	100	0	0.0
7	55F,10A	1.1	75	25	0.3
8	59M,25C	3.3	75	25	0.3
9	60F,11A	10.1	25	75	3.0
10	63F,3A	2	75	25	0.3
11	64F,10A	3	70	30	0.4
12	65M,22C	2.4	50	50	1.0
13	66F,26C	0	75	5	0.1
14	66M,17A	12.1	25	75	3.0
15	67M,7A	5.6	75	25	0.3
16	71F,2A	8.4	50	50	1.0
17	71M,12C	8.9	25	75	3.0
18	72M,8C	4.6	75	25	0.3
19	74F,17C	9.6	50	50	1.0
20	74F,62C	3	25	75	3.0
21	75M,20C	3.1	75	25	0.3
22	77F,53C	9.2	25	75	3.0
23	78M,19C	8	25	75	3.0

Table S2: Human PARP1 and 2 enzyme inhibition, IC50 values determined for inhibitors used in Fig 6.

Inhibitor	IC50	
	PARP1	PARP2
PJ-34	86.5 nM (R^2 = 0.94)	282 nM (R^2 = 0.99)
Niraparib	205.3 nM (R^2 = 0.99)	>100 µM
Olaparib	9.5 nM (R^2 = 0.97)	>100 µM
Rucaparib	8.8 nM (R^2 = 0.99)	>10 µM
Veliparib	9.3 nM (R^2 = 1)	11.1 µM (R^2 = 0.84)
Minocycline	204.5 µM (R^2 = 0.69)	2.8 µM (R^2 = 0.82)
